# Is Motorized Treadmill Running Biomechanically Comparable to Overground Running? A Systematic Review and Meta-Analysis of Cross-Over Studies

**DOI:** 10.1007/s40279-019-01237-z

**Published:** 2019-12-04

**Authors:** Bas Van Hooren, Joel T. Fuller, Jonathan D. Buckley, Jayme R. Miller, Kerry Sewell, Guillaume Rao, Christian Barton, Chris Bishop, Richard W. Willy

**Affiliations:** 1 Department of Nutrition and Movement Sciences, NUTRIM School of Nutrition and Translational Research in Metabolism, Maastricht University Medical Centre+, Universiteitssingel 50, 6229 ER Maastricht, The Netherlands; 2 Institute of Sport Studies, Fontys University of Applied Sciences, Eindhoven, The Netherlands; 3 Department of Health Professions, Faculty of Medicine and Health Sciences, Macquarie University, 75 Talavera Rd, Macquarie Park, NSW 2109 Australia; 4 Alliance for Research in Exercise, Nutrition and Activity (ARENA), School of Health Sciences, University of South Australia, Adelaide, SA 5001 Australia; 5 East Carolina University, Greenville, NC USA; 6 Aix Marseille University, CNRS, ISM, Marseille, France; 7 La Trobe Sports and Exercise Medicine Research Centre, School of Allied Health, La Trobe University, Bundoora, VIC Australia; 8 The Biomechanics Lab, Adelaide, SA Australia; 9 School of Physical Therapy and Rehabilitation Sciences, University of Montana, Missoula, MT USA; 10 Department of Surgery, St Vincent’s Hospital, University of Melbourne, Melbourne, Australia

## Abstract

**Background:**

Treadmills are often used in research, clinical practice, and training. Biomechanical investigations comparing treadmill and overground running report inconsistent findings.

**Objective:**

This study aimed at comparing biomechanical outcomes between motorized treadmill and overground running.

**Methods:**

Four databases were searched until June 2019. Crossover design studies comparing lower limb biomechanics during non-inclined, non-cushioned, quasi-constant-velocity motorized treadmill running with overground running in healthy humans (18–65 years) and written in English were included. Meta-analyses and meta-regressions were performed where possible.

**Results:**

33 studies (*n *= 494 participants) were included. Most outcomes did not differ between running conditions. However, during treadmill running, sagittal foot–ground angle at footstrike (mean difference (MD) − 9.8° [95% confidence interval: − 13.1 to − 6.6]; low GRADE evidence), knee flexion range of motion from footstrike to peak during stance (MD 6.3° [4.5 to 8.2]; low), vertical displacement center of mass/pelvis (MD − 1.5 cm [− 2.7 to − 0.8]; low), and peak propulsive force (MD − 0.04 body weights [− 0.06 to − 0.02]; very low) were lower, while contact time (MD 5.0 ms [0.5 to 9.5]; low), knee flexion at footstrike (MD − 2.3° [− 3.6 to − 1.1]; low), and ankle sagittal plane internal joint moment (MD − 0.4 Nm/kg [− 0.7 to − 0.2]; low) were longer/higher, when pooled across overground surfaces. Conflicting findings were reported for amplitude of muscle activity.

**Conclusions:**

Spatiotemporal, kinematic, kinetic, muscle activity, and muscle–tendon outcome measures are largely comparable between motorized treadmill and overground running. Considerations should, however, particularly be given to sagittal plane kinematic differences at footstrike when extrapolating treadmill running biomechanics to overground running. Protocol registration CRD42018083906 (PROSPERO International Prospective Register of Systematic Reviews).

**Electronic supplementary material:**

The online version of this article (10.1007/s40279-019-01237-z) contains supplementary material, which is available to authorized users.

## Key Points

**Table:** 

Spatiotemporal parameters, kinematic, kinetic, muscle activity, and muscle-tendon outcome measures are largely comparable between motorized treadmill and overground running.
Motorized treadmill running differs from overground on a number of sagittal plane outcome measures, including sagittal foot-ground angle at footstrike, knee flexion at footstrike, and knee flexion range of motion during stance, and vertical displacement of the pelvis.
Conflicting findings were reported for the amplitude of muscle activity, with some studies reporting lower muscle activity during treadmill running and other studies reporting no differences.

## Introduction

Motorized treadmills (MT) are often used for research, clinical practice, and training purposes. The 2017 United States national runners survey for instance found that 14% of the runners preferred to run on a MT [1] and MTs are also often used as a supplementary training mode among elite athletes [2]. In clinical settings, MT running is increasingly combined with video analysis to investigate running technique and inform footwear, orthotic, and gait retraining strategies for performance enhancement, injury prevention and rehabilitation [3–5]. MTs are also used during rehabilitation to commence running in a controlled environment [6, 7]. Finally, instrumented MTs are frequently used in research settings to evaluate running biomechanics [8–12].

Several studies have reported differences in running biomechanics between MT and overground running [6, 13–16], although the evidence across studies is often conflicting. Biomechanical differences between MT and overground running may arise from a variety of aspects. A widely held belief is that MT running requires less propulsion as the belt moves the supporting leg under the body rather than the body moving over the supporting leg [17]. van Ingen Schenau [18] investigated this issue and showed that MT and overground running are theoretically similar when using a coordinate system that moves with the belt, when belt speed is constant and air drag (resistance) is negligible. However, experimental studies have shown that belt speed is not constant and instead decelerates at foot strike and accelerates at toe-off [14, 15, 17, 19, 20], thereby potentially altering running biomechanics. Further, faster speeds require higher stride frequencies which increases air resistance during both treadmill and overground running. However, air resistance increases more with increases in running speed during overground running, because the body moves through the air, and this could introduce biomechanical differences at higher running speeds [21, 22]. MT running biomechanics can also be affected by the familiarity/comfort with MT running [18, 23, 24], visual focus [25], belt dimensions [14] and differences in perception [26], surface hardness [7, 27], and mechanical treadmill model [28] compared to overground running.

Although a large body of research has investigated biomechanical differences between MT and overground running, there has been no systematic review on this topic. Therefore, the objective of this systematic review and meta-analysis was to synthesize evidence from crossover studies that investigated biomechanical differences between overground and MT running. This systematic review will highlight whether the findings of individual studies are consistent or contradictory and avoid issues associated with inferring results from single studies that often have relatively small sample sizes. Further, this review will also provide an overview of the factors that may influence differences between MT and overground running biomechanics and provide suggestions on strategies to reduce biomechanical differences between MT and overground running. The findings of this review will, therefore, be useful for (1) athletes and coaches to better understand the specificity of MT running for improving overground running performance, (2) researchers to better understand the validity of MT running and the generalizability of MT running biomechanics to overground running, and (3) clinicians that use MT running during rehabilitation or to investigate running biomechanics to inform footwear, orthotic and gait retraining strategies for performance enhancement, injury prevention and rehabilitation.

## Methods

### Registry of Systematic Review Protocol

A systematic review of the literature was performed using guidelines in the Cochrane Handbook for Systematic Reviews of Interventions (version 5.1.0) and following the checklist for the Preferred Reporting Items for Systematic reviews and Meta-Analyses 2015 (PRISMA) [29]. The protocol was prospectively registered with the International Prospective Register of Systematic Reviews (PROSPERO; registration number CRD42018083906) and was part of a larger systematic review project comparing MT and overground running across a range of variables [30]. Registration occurred after searches had been conducted, but before screening was completed.

### Information Sources

A librarian (KS) searched four electronic databases to avoid a biased literature sample: MEDLINE via PubMed, SPORTDiscus, Web of Science, and Embase. The searches covered all dates of available literature as of June, 2017, with the date of the last search being June 9, 2017. No limits were applied for language within each database to prevent excluding articles that were not assigned a language. Search alerts were created to monitor any new search results after the date of the last search up to June 20, 2019. Any articles identified by this search that were deemed to be relevant (based on title and abstract) were sent to two researchers (RW and GR) for full-text eligibility assessment. Another researcher (BVH) double checked the included papers from this assessment and modified the eligibility criteria to limit the scope of the review. Hand searching of reference lists and forward citation searching of included studies was also used to identify articles.

### Eligibility Criteria

To be included, studies had to be (1) crossover studies comparing non-inclined, non-cushioned, quasi-constant-velocity MT and overground running; (2) performed among healthy human individuals between 18 and 65 years; (3) focused on biomechanical variables of the legs or pelvic area, such as joint angles, ground reaction forces, muscle activity and muscle–tendon unit interaction; and (4) written in English. Conference abstracts were excluded due to the difficulty in obtaining full methods and complete data sets, and hence in assessing risk of bias and data analysis. Theses were also excluded because it was often unclear whether they were published as an original article and included in the review, which would have led to assigning double the weight to the same study in meta-analysis. Data on sprinting (defined here as > 25 km/h or > 7 m/s) [31] were excluded because most commercial treadmills cannot reach the speed threshold above which we consider running to be sprinting. Barefoot running and running in a fatigued status were also excluded. Studies with < 3 participants and studies that used a substantially (> 10% difference) different running speed during the overground and MT trials were excluded. Studies that did not specify whether a motorized or non-motorized treadmill was used were assumed to use a MT as this is traditionally the most frequently used treadmill in research and practice.

### Search Strategy

A PICO strategy was used to build search criteria for electronic databases. The PICO consisted of terms for running, treadmills, and overground surfaces. The search strategy was mapped to appropriate subject headings for each of the databases used for this review. The search string used for MEDLINE/PubMed is reported in Supplementary file I.

### Study Selection

Duplicate references were removed first by systematic review software (Rayyan, QatarComputing Research Institute, Doha, Qatar) [32] and then manual methods. Two authors (RW and GR) independently screened titles and abstracts to determine initial eligibility using systematic review software (Rayyan). Blinding of authors was used to reduce bias during this process. Finally, the authors reviewed the full-text to determine eligibility for inclusion based on the eligibility criteria. Disagreements in eligibility decisions were resolved through discussion, or with a third reviewer (BVH) when required.

### Data Collection Process

Data extraction was completed independently by four authors (BVH, JF, JM and CBa) using a standardized form that was pilot-tested on ten randomly selected included studies and refined accordingly. The data were then merged by one author (BVH) and any discrepancies in the extracted data were resolved through discussion, or with a third reviewer (JF) consulted if required. Extracted data from each full-text article included (1) study identification information, (2) study design, (3) sample size, (4) gender, (5) age, height and body mass, (6) running ability (e.g., weekly distance), (7) experience with MT running, (8) MT brand, model, motor power, and belt dimensions, (9) description of overground condition (e.g., length, surface), (10) running velocities in both conditions, (11) time between conditions, (12) duration of familiarization with MT running, (13) means and standard deviations for relevant outcome measures and (14) an exact *p* value, *t* value, or confidence intervals for the comparison between conditions. If insufficient data were reported, the authors were contacted by-email. When data were not presented in tables or text and when authors did not provide the requested data, these were extracted from figures using WebPlot Digitizer (Web Plot Digitizer, V.4.1. Texas, USA) [33] where possible.

### Risk of Bias Assessment

After the literature search and selection, a risk of bias assessment was performed independently by two authors (JB and CBi) using a modified Cochrane Collaboration’s tool for assessing risk of bias in randomised trials [34]. More information on the criteria used in risk of bias assessment can be found in Supplementary file II. Risk of bias was assessed based on the information reported in the published paper and not on information provided by authors for Table 1. Disagreements in risk of bias assessment was resolved by discussion before the scores were merged into a spreadsheet. Mean kappa agreement between the authors was 0.99 (nearly perfect). Risk of bias was considered in the interpretation of the results by applying the Grading of Recommendations Assessment, Development and Evaluation (GRADE) system [35]. Briefly, the overall quality was rated as high and downgraded one level to moderate, low or very low for each of the following limitations: total sample size < 100 participants (imprecision), high statistical heterogeneity (inconsistency), more than 50% of studies in meta-analysis had > 1 risk of bias item assessed to be high-risk (risk of bias).

**Table 1:** Study characteristics

Author (year)	Participants info		Conditions		
	M/F; mean ± SD age (years)	Overground; treadmill running ability	Treadmill condition (brand and model; motor power; belt dimensions; speed in km/h)^a^	Overground condition (length and surface; speed)	Time between conditions; familiarisation
Asmussen et al. [28] (2018)	7/4; 26 ± 2	Running 1–4 times p/w; unclear	Quinton Q65 research treadmill with unclear motor power (Quinton Instrument Co., Seattle, WA, USA); 1.4 × 0.51 m; 9.7, 13 and 16.2 km/h	Unclear length indoor lab runway; 9.7, 13 and 16.2 km/h	Unclear, unclear
			Healthrider H20T with 1.5 kW motor (ICON Health & Fitness, Logan, UT, USA); 1.27 × 0.41; similar speeds to other TMs		
			Bertec TM-09 instrumented split-belt TM with 2.6 kW motor (Bertec, Columbus, OH, USA); 1.75 × 0.5 m; similar speeds to other TMs		
Barton et al. [52] (2015)	15/11; 27 ± 7	Unclear; unclear	HP Cosmos (Nussdorf-Traunstein, Germany); model, motor power and belt dimensions unclear; self-selected pace (speed unclear)	Unclear length outdoor track; unclear	Unclear; 2–5 min and participants reported feeling comfortable
Baur et al. [51] (2007)	14/0; 37 ± 10	50 ± 17 km/w; > 3 h TM running experience	HP Cosmos Quasar with 3.3 kW motor (Nussdorf-Traunstein, Germany); 1.7 × 0.65 m; 12 km/h	400 m track; 12 km/h	5–8 days; ~ 3–10 min
Bigelow et al. [53] (2013)	Total sample size 12; 33 ± 10	>3 h/w for > 6 w and previous TM running experience	Precor 9.33 with 2.2 kW motor (Precor, Woodinville, WA); 1.42 × 0.56 m; 4-mile run pace matched to OG	200 m indoor synthetic track; self-selected 4 mile pace (speed unclear)	7 days; unclear
Brookes et al. [43] (1971)	2/2; 33 ± 5	Trained athletes; unclear	Unclear brand, model, motor power and belt dimensions; running speed matched to OG	Road of unclear length; 10.1, 14.8, 18.5 and 19.6 km/h.	Unclear, unclear
Chambon et al. [41] (2015)	12/0; 22 ± 2	Recreational runners; unclear	“Stiff” TM, HEF Tecmachine Medical Development S1200 (Andrézieux-Bouthéon, France); unclear motor power and belt dimensions; running speed matched to OG	15 m concrete runway in lab; preferred running speed ± 5% as determined on TM (speed unclear)	~ 20 min, ~ 7 min familiarization
Cronin, Finni [54] (2013)	10/0; 29 ± 4	Habitual runners; previous TM running experience	OJK-1, Telineyhtymä, Kotka, Finland; unclear motor power and belt dimensions; 10.2 km/h (matched to OG)	Unclear length indoor runway; self-selected speed (10.3 km/h)	Unclear, 6 min walking and 3 min running
Elliott, Blanksby [13] (1976)	12/12; 24 ± –	Regular non-competitive runners running > 20 km/w; > 10 sessions TM running experience	Unclear brand, model, motor power and belt dimensions; speed matched to OG	200 m track; freely selected jogging and running speed of < 12 km/h for 3 laps, then > 12 km/h for 2 laps. Male group: 13.3 km/h for jogging, 19.5 km/h for running, females: 14.3 km/h for jogging, 19.0 km/h for running	Unclear, 6 min at 11.9 km/h
Fellin et al. [55] (2010)	10/10; 25 ± 9	Recreational runners, > 16 km/w; score 9/10 for comfort with TM running	Quinton TM, model and motor power unclear (Cardiology Inc., Bothell, WA, USA); unclear belt dimensions; 12.1 km/h	25 m runway in lab with standard floor tiles; 12.1 km/h ± 5%	‘A few minutes’, 2 min during w-up and 3 min at 12.1 km/h
Firminger et al. [56] (2018)	11/0; 30 ± 8	Recreational runners; familiar with MT running	Bertec TM-09 instrumented split-belt treadmill with 2.6 kW motor (Bertec, Corporation, Columbus, OH); 1.75 × 0.5 m; 10 and 13 km/h ± 5%	5.8 m wooden runway with 9 m run-up; 10 and 13 km/h ± 5%	~10 min; ~ 30 s
Fu et al. [7] (2015)	13/0; 24 ± 1	20.4 ± 5.2 km/w with average of 3.5 years running experience on TM and OG	‘Normal’ TM with 0.7 kW motor (SH-A5188; Shuhua Co. Ltd., Jinjiang, China) 1.25 × 0.44 m	30 m straight concrete, track and grass runways; 12.0 ± 0.6 km/h	5 min; 5 min
			TM with 0.9 kW motor cushioned with 5 cm ethylene vinyl acetate (SH-5199; Shuhua Co. Ltd.); 1.3 × 0.46 m; 12 km/h for both TM’s		
Fullenkamp et al. [48] (2018)	10/10; 23 ± 4	Unclear, prior experience with TM running	True fitness Technology, model and motor power unclear (St. Louis, MO); unclear belt dimensions; speed matched to OG	Unclear length, indoor lab runway; self-selected pace of 9.4 ± 1.8 km/h	5 min; 60 s before data collection and 1 familiarization session
Garcia-Perez et al. [57] (2013)	17/10; 34 ± 8	Maximum aerobic speed: 16.1 ± 1/6 km/h; unclear	Excite Run 700 with 4.4 kW motor (TechnogymSpA, Bambettola, Italy); 2.18 × 0.94 m; 400 m at 12.0 km/h and at 14.4 km/h	400 m track; 400 m at 12.0 km/h and 400 m at 14.4 km/h	7 days; 15 min
Garcia-Perez et al. [58] (2014)	11/9; 34 ± 8	49.8 ± 17.8 km/w, with on average 9.5 years running experience; unclear	Excite Run 700 with 4.4 kW motor (TechnogymSpA, Bambettola, Italy); 2.18 × 0.94 m; 400 m at 14.4 km/h	400 m rubberized track; 400 m at 14.4 km/h	At least one day; 15 min
Hong et al. [27] (2012)	16/0; 23 ± 2	University long distance team, > 20 km/w; experience with TM running	6300HR, SportsArt Fitness with 2.9 kW motor (Woodinville, WA, USA); 1.47 × 0.5 m; 13.7 km/h	30 m straight concrete and natural grass (soccer field) runways; 13.7 km/h ± 5%	Unclear; 6 min at 11.9 km/h
Kluitenberg et al. [40] (2012)	12/12; age range 18–35	Track and field club members training > 2x/w for > 1 year; unclear	Instrumented TM with 1.8 kW motor (Entred, Forcelink, Culemborg, The Netherlands); 1.60 × 0.60 m; speed matched to OG for all conditions	17.5 m runway in lab; three speeds: normal endurance run (mean = 12.3 km/h), slower speed (running speed during a warming-up; mean = 11.0 km/h), and a faster speed (10 km race speed; mean = 14 km/h	Unclear, 10 min at 10 km/h
Lindsay et al. [59] (2014)	9/0; 28 ± 7	Trained distance runners running 49.7 ± 24.4 km/w; TM experience not assessed	HP Cosmos VIASYS LE 500CE 170/65 with 2.2 kW motor (Nussdorf-Traunstein, Germany); 1.7 × 0.65 m; 8 min at preferred and 80% & 120% of preferred speed	140 m indoor oval running track; preferred and 80% and 120% of preferred speed (speed unclear)	48 h; unclear
Meinert et al. [60] (2016)	20/0; 23 ± 3	20.35 ± 8.11 km/w with 5.4 ± 3.2 years of running experience; unclear	Woodway ERGO XELG 90 with 3.7 kW motor (Woodway USA INC., Waukesha, WI, USA); unclear belt dimensions; 10.4 km/h	30 m concrete runway; 10.4 km/h (between 9.7 – 11.9 km/h)	Unclear, 6 min
Milgrom et al. [6] (2003)	2/1; 39 ± 16	Recreationally active runners (males) or tennis player (female); prior experience with treadmill running	Unclear brand, model, motor power and treadmill dimensions (in personal communication, author mentioned that TM was likely longer and wider than most TM’s used in fitness centers); 11 km/h	100 m straight runway of 400 m track with asphalt; 11 km/h	< 1 h; Familiarization session 1 week before data collection and additional 30 s before data collection
Montgomery et al. [61] (2016)	15/0; 24 ± 4	Recreationally active individuals; already familiar with OG and TM locomotion	Woodway, ELG55 with 3.68 kW motor, Woodway (Weil an Rhein, Germany); 2.44 × 0.69 m; speed matched to OG speed	40 m indoor lab runway with rubber surface; jogging at 10.4 ± 1.3 km/h and running at 15.4 ± 1.3 km/h	4–5 min; familiarization was undertaken > 48 h before the main testing, and involved walking, jogging and running at a constant speed on a non-motorized TM
Nelson et al. [49] (1972)	16/0; 20 ± –	Experienced runners, including sprinters, middle distance and cross country runners; 6 TM practice sessions	Quinton Instruments, Model 18–60 with 2.2 kW motor (Cardiology Inc., Bothell, WA, USA); 1.52 × 0.46 m; speed matched to OG	Indoor platform of ‘relatively long distance’; 12.1, 17.6 and 23 km/h	Separate days; separate familiarization sessions
Nigg et al. [14] (1995)	Total sample size 22; age unclear	11 runners running 70.9 ± 37.7 km/w and 11 non-runners; most runners had experience with TM running	Three TM’s: large TM with 2.5 kW motor (Q2), 2 × 0.6 m; midsize TM with 2.5 kW motor (Q6); 1.65 × 0.51 m; and small TM with 1.1 kW motor (A); 1.3 × 0.4 m; 10.8, 16.2, 18.0 and 21.6 km/h on all TM’s except for the small TM	30 m indoor lane in lab with ‘sport surface’; speed matched to TM	Unclear; subjects ran on the TM before data collection until they felt comfortable
Oliveira et al. [50] (2016)	12/0; 28 ± 4	Regular runners running 2–3x/w; previous TM running experience	WoodwayPro with 1.5 kW motor (Woodway USA INC., Waukesha, WI, USA); 1.73 × 0.7 m; preferred speed (10.8 km/h)	75 m straight indoor corridor; preferred speed determined on TM (10.8 km/h)	1–2 min; 5–10 min
Pink et al. [62] (1994)	5/9; 32, range 24–45	Recreational runners, > 16 km/w; unclear	Unclear brand, model, motor power and belt dimensions; matched to OG	15 m surface in lab; self-selected warm-up (slow, 10.6 km/h) and training (fast, 14.2 km/h) paces. Subjects who selected < 12.1 km/h were used for slow pace (*n *= 8) and subjects faster than 12.9 km/h were used for fast pace (*n *= 14)	Unclear; unclear
Riley et al. [63] (2008)	10/10; 25 ± 5	Regular runners, > 24 km/w; all experience with TM running	Instrumented TM of unclear brand with 4.7 kW motor; 2.80 × 0.66 m; speed matched to OG (13.9 ± 2.2 km/h)	15 m runway in lab; self-selected 10 km race pace (13.8 ± 2.3 km/h)	Unclear; 5 min
Roussos et al. [64] (2019)	10/0; 21 ± 3	Physically active individuals; at least 6 months 1–2 p/w TM	Pegasus T1600 with 2.2 kW motor power (United Kingdom); 1.48 × 0.53 m; 11 ± 1 km/h	20 m concrete runway; 11 ± 1 km/h	24 h; 15 s
Schache et al. [19] (2001)	9/1; 28 ± 5	Active runners, > 20 km/w; experienced TM runners	Custom made TM with a 15 kW motor (Sportech Gymnasium and Electronic Sports Equipment, Jamison, ACT, Australia); 3.5 × 1.1 m; speed matched to OG (14.3 km/h)	40 m indoor synthetic runway; self-selected pace of 14.4 km/h	On average 8.2 ± 3.4 days; 3 min
Sedighi et al. [65] (2019)	14/0; 23 ± 6	Recreationally active, > 25 km/w; previous TM running experience	Unclear model, motor power and belt dimensions; 12 km/h	25 m laboratory floor; 12 km/h ± 5%	Unclear, 6 min at 10.8 km/h
Sinclair et al. [66] (2013)	11/1; 23 ± 4	Recreationally active, > 25 km/w; previous TM running experience	Woodway ELG with 5.45 kW motor (Weil am Rhein, Germany); 2.0 × 0.55/0.70; 14.4 km/h	22 m laboratory floor (altrosports 6 mm, Altro Ltd, Letchworth Garden City, Hertfordshire, UK); 14.4 km/h	Unclear; 5 min
Sinclair et al. [67] (2014)	12/0; 24 ± 1	Unclear; unclear	Woodway ELG with 5.45 kW motor (Weil am Rhein, Germany); 2.0 × 0.55/0.70; 14.4 km/h	22 m laboratory floor (altrosports 6 mm, Altro Ltd, Letchworth Garden City, Hertfordshire, UK); 14.4 km/h ± 5%	Unclear; 5 min
Wang et al. [68] (2014)	13/0; 22 ± 4	Experienced runners, > 20 km/w; experience with TM running	6300HR, SportArt Fitness with 3.3 kW motor (Woodway USA INC., Waukesha, WI, USA); 1.47 × 0.5 m; 13.7 km/h	30 m straight runway; 13.7 km/h	Unclear; 6 min at 11.9 km/h
Wank et al. [69] (1998)	10/0; 28 ± 3	Physically active in different sports; familiar with TM running	Woodway with a 2.2 kW motor (Woodway USA INC., Waukesha, WI, USA); 2.0 × 0.7 m; 14.4 and 21.6 km/h	45 m indoor track with tartan surface; 14.4 and 21.6 km/h	Unclear; unclear
Willy et al. [16] (2016)	9/9; 24 ± 4	Habitual runners, 36.7 km/w with 7.4 ± 3.6 years of running experience; only participants comfortable with TM running included	Bertec TM-09 instrumented split-belt TM with 2.6 kW motor (Bertec Corporation, Columbus, OH); 1.75 × 0.5 m; Self-selected speed of 10.4 km/h	25 m runway with laminate tiles on a cement floor; same self-selected speed as TM (10.4 km/h ± 3%)	10 min; 6 min

*F* female, *M* male, *OG* overground, *SD* standard deviation, *TM* treadmill

^a^Treadmill motor power and belt dimensions were obtained from the authors or manufacturer specifications if not provided in the paper. Similarly, if relevant information on other aspects such as familiarisation period was not report but provided by the authors, this was included in the table

### Statistical Analysis

A separate random-effects meta-analysis for each review outcome was performed using the Metafor statistical package in R software (version 3.5.2, R Foundation for Statistical Computing) [36] when two or more studies reported on the same outcome. Sub-group analyses were performed with overground surface (i.e., track, lab runway, concrete, and grass) as the categorical outcome. Meta-regression was performed when at least six effects were available for an outcome [37] using the following variables as continuous covariates: running speed, treadmill motor power and treadmill belt length and width. Multiple study effects were included for studies that used multiple overground surfaces, treadmills or speeds. In this case, the sample size of the study was divided evenly among the effects to avoid assigning more weight to these studies [38]. When participants could not be divided evenly among the effects (e.g., nine participants for two effects), the remaining participant was allocated to the stiffer surface (e.g., concrete) as this best reflects the running environment of recreational runners [1, 39] or to the higher running speed to increase statistical power as most studies used relatively slow to moderate speeds. When multiple study effects were reported that were not of direct interest to this review (e.g., separate effects for the left and right leg [19], separate effects for heel-strike and non-heel-strike runners [40], and separate effects for shoes with different rearfoot midsole thicknesses [41]), a combined effect was computed across these outcome measures as detailed by Borenstein et al. [42] for dependent continuous outcomes. One study reported separate effects for males and females [13]. Since they ran at different speeds, the effect was not combined into one effect, but the participants were divided among the speeds. Brookes et al. [43] reported stride frequency data for four speeds, but only included four participants. To have > 1 participant per condition, we only included only the lowest and highest running speed in the analysis. Similarly, Asmussen et al. [28] reported foot pressure for three different treadmills and at three different speeds. To have ≥ 3 participant per condition (required for computing Hedge’s *g*_rm_), we only included the highest speed for the commercially available Healthrider treadmill and the lowest and highest speed for the Bertec research instrumented treadmill.

Individual studies were weighted using the inverse variance method. If studies reported data to compute a mean difference and an exact *p* value without reporting the variance of the mean difference, we calculated the variance based on the equivalent T-statistic. Where sufficient information was available, the correlation between treadmill and overground running measurements was also estimated. However, it was often unclear whether studies reported a Bonferroni-corrected or uncorrected *p* value. As a result, the estimated correlation coefficient was often implausible (e.g., > 1). Therefore, a default correlation coefficient of 0.50 was used in all meta-analyses [44]. This ensured that the maximum number of studies were included. Meta-analysis was performed using raw mean differences when included studies reported outcomes in the same units or when data could be converted into same units. Standardized mean differences were calculated when included studies reported outcomes in different units by dividing the mean difference by the mean standard deviation, while correcting for small sample bias (Hedge’s *g*_rm_) [45]. The between-subject standard deviation from another study that reported the same outcome measure and used a similar speed was used if no standard deviation or other data to estimate the standard deviation was reported. Standardized mean differences were considered trivial (< 0.20), small (0.20–0.59), moderate (0.60–1.19), large (1.20–1.99), and very large (≥ 2.00) [46]. Heterogeneity was assessed using the I^2^ and T statistic. I^2^ represents the percentage of total variation in estimated effects across studies due to heterogeneity rather than chance and was interpreted as small (*I*^2^ < 25%), moderate (*I*^2^ = 25–49%), and high (*I*^2^ > 50%) [47]. The T statistic represents the standard deviation of the true effects and is reported in the same scale as the meta-analysis [42]. For the meta-analyses, limb angular kinematics were expressed such that positive values corresponded to (a) sagittal plane measures of hip flexion, knee extension and dorsiflexion; (b) frontal plane measures of hip adduction, knee adduction and ankle eversion; (c) transverse plane measures of internal rotation of the hip, knee and ankle.

### Publication Bias

Publication bias was not assessed because there was only a small number of studies included in most meta-analyses. and we did not see any reason why studies reporting no difference between treadmill and overground conditions would be less likely to be published than studies finding a statistically significant difference.

## Results

### Search Results

**Fig. 1:**
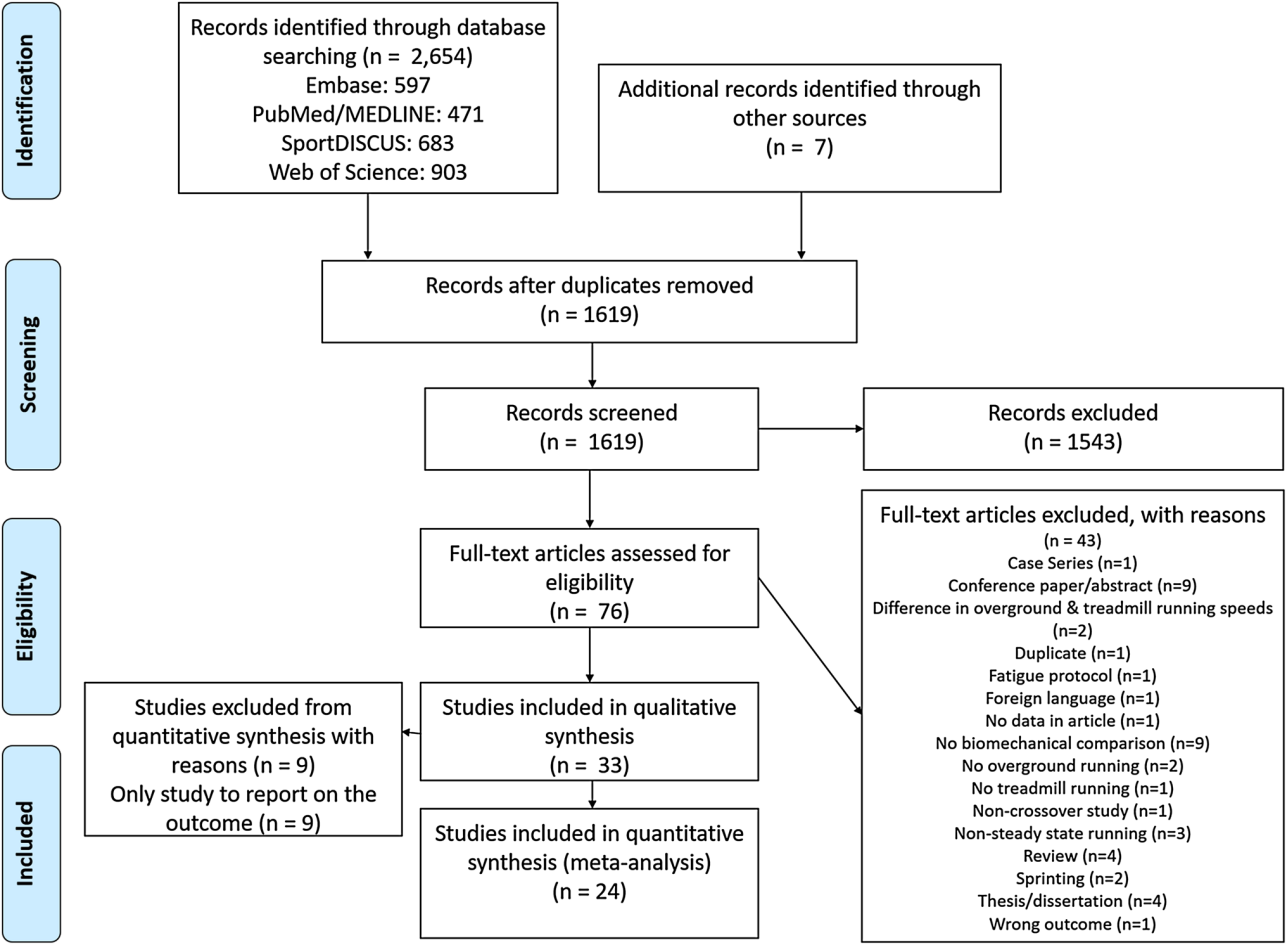
Literature search flow chart. *n* number of studies

The initial literature search yielded 2654 records through electronic databases (Fig. 1). Title and abstract screening resulted in exclusion of 1543 records. Forward citation searching for articles that passed title/abstract screening yielded 489 additional records to be screened, and five of these were included in the review. Monitoring newly published, relevant literature yielded an addition two records for consideration in the review. After screening 76 records for inclusion/exclusion criteria, 43 records were rejected, resulting in 33 articles being included in the review.

### Study Characteristics

Detailed study characteristics are reported in Table 1. All 33 studies included in this review were crossover studies that compared MT to overground running. The total number of participants in the included studies was 494 (349 males, 111 females, 34 not specified). Of the 33 included studies, 16 included males only, 15 a mix of males and females and two did not specify gender. 30 studies recruited participants that were runners or physically active in other sports, and three studies did not specify the physical activity of the participants. 21 studies further specified that the participants had prior experience with MT running, while this information was unclear in other studies. 23 studies specified the motor power and belt dimensions, or provided enough data to gather this information. In the overground conditions, nine studies used a synthetic track, three studies used a concrete road, 19 used an indoor lab runway and two studies used a combination of multiple overground surfaces (track, grass, concrete). Running speeds ranged from 9.4 km/h [48] to 23.0 km/h [49] and were not specified in four studies. Similarly, the time between the MT and overground condition varied between 1 min [50] to 8 days [19, 51] and was not specified in 16 studies. Finally, different approaches were used to familiarize the participants with MT running before data collection. Fourteen studies provided < 6 min of familiarization immediately before data collection, ten studies provided ≥ 6 min, and nine studies did not specify the familiarization procedure.

### Risk of Bias Assessment

**Fig. 2:**
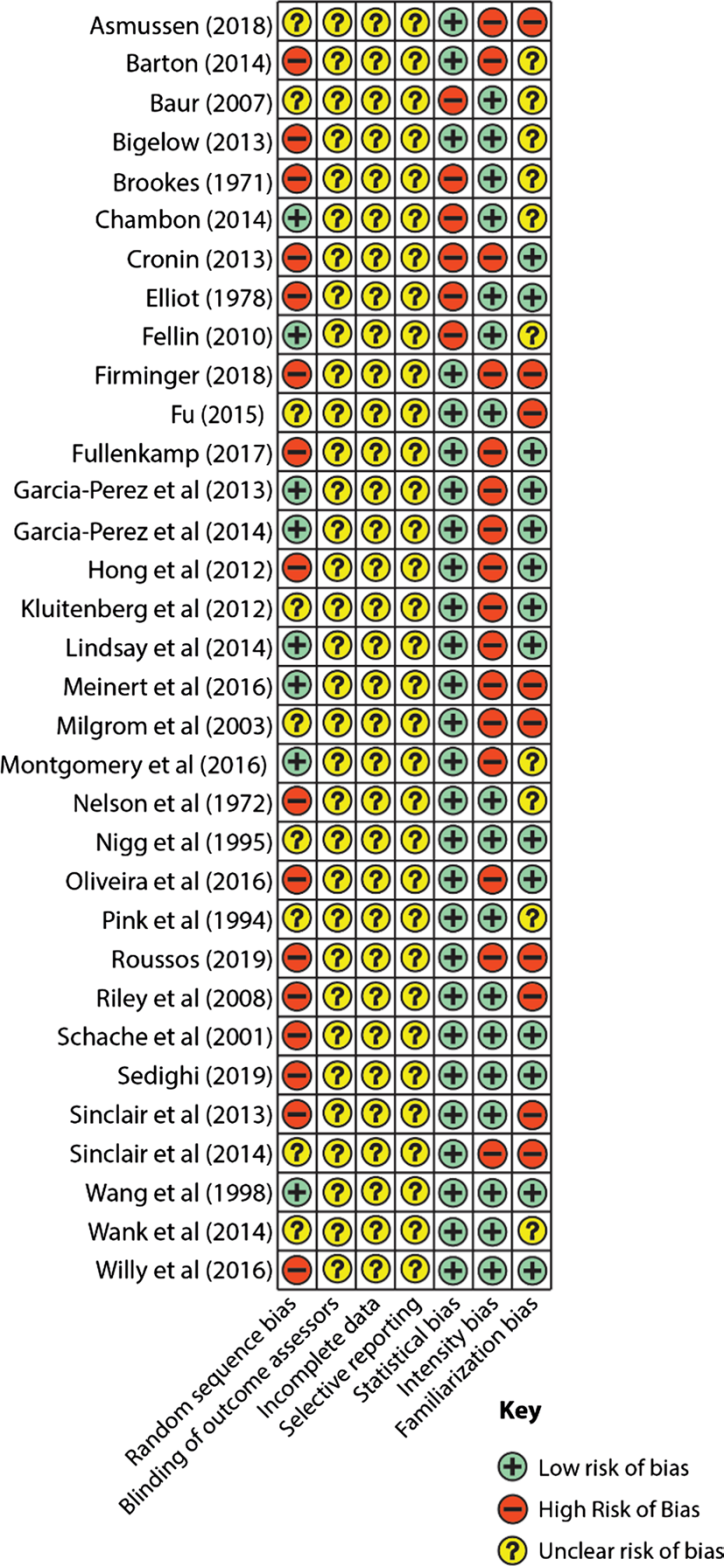
Risk of bias assessment for all included studies

The risk of bias score of included studies is reported in Fig. 2.

### Spatiotemporal Outcome Measures

**Fig. 3:**
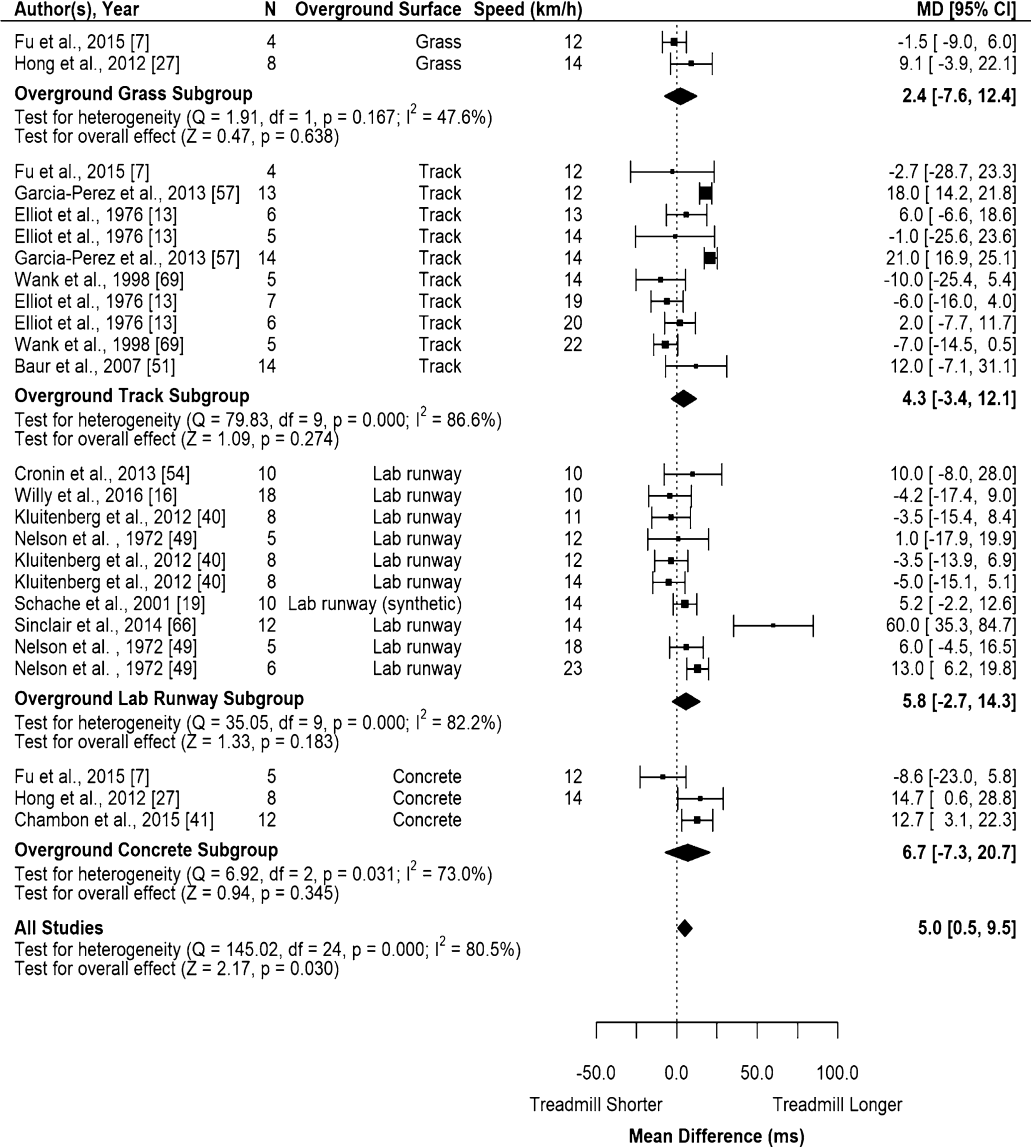
Random-effects meta-analysis of ground contact time during MT compared to overground running. Subgroup analysis based on overground surface with subgroups organized from least stiff surface to stiffest surface and studies organized from slowest to fastest speed. *CI* confidence interval, *df* degrees of freedom, *MD* mean difference, *N* sample size

**Fig. 4:**
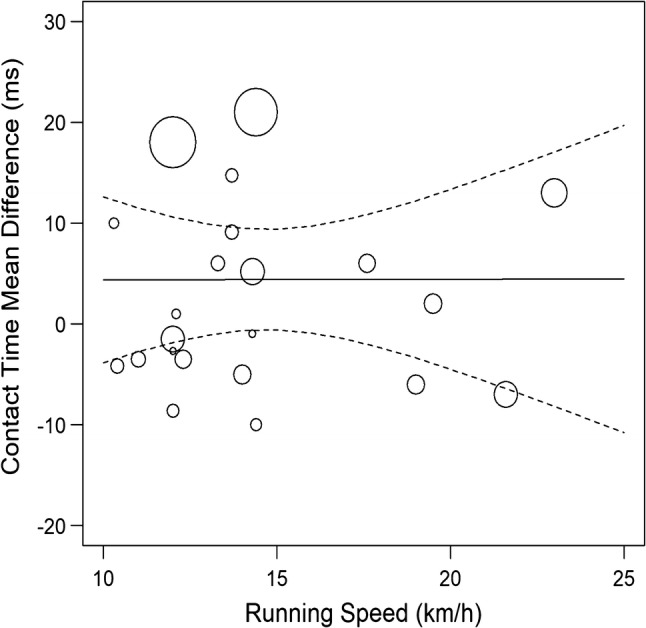
Random-effects meta-regression of contact time during MT compared with overground running based on running speed. Larger data points received greater weighting than smaller data points. Solid lines represent the estimated relationship and dashed lines represent the upper and lower 95% confidence limits

MT running did not significantly affect ground contact time when compared to track, concrete, lab runway or grass surfaces individually (Fig. 3 and Supplementary file IV). When combined across all overground surfaces, MT running resulted in a significant longer ground contact time by 5 ms (95% CI 0.48–9.51; Fig. 3 and Table 2 for GRADE quality evidence). MT running speed, motor power, and belt dimensions were no statistically significant moderators of the mean difference in contact time (Fig. 4, Supplementary file IV).

**Table 2:** Summary of meta-analysis findings and quality of evidence synthesis

Outcome	Summary of findings				Quality of evidence synthesis (GRADE)			
	*k*	*n*	Effect (95% CI)	Direction effect compared to overground	Imprecision	Inconsistency	Risk of bias	Overall quality
Spatiotemporal outcomes								
*Contact time (ms)*								
Track	10	79	4.3 (− 3.4 to 12.1)	↔	− 1	− 1	None	Low
Concrete	3	25	6.7 (− 7.2 to 20.7)	↔	− 1	− 1	None	Low
Lab runway	10	90	5.8 (− 2.7 to 14.3)	↔	− 1	− 1	None	Low
Grass	2	12	2.4 (− 7.6 to 12.4)	↔	− 1	− 1	None	Low
All	25	106	5.0 (0.48 to 9.5)	↑	None	− 1	None	Low
*Stride time (ms)*								
Track	5	38	− 19.4 (− 46.6 to 7.8)	↔	− 1	− 1	None	Low
Lab runway	8	71	− 7.4 (− 22.6 to 7.8)	↔	− 1	− 1	None	Low
All	13	109	− 12.0 (− 25.8 to 1.8)	↔	None	− 1	None	Low
*Stride length (cm)*								
Track	8	61	− 9.6 (− 20.6 to 1.5)	↔	− 1	− 1	None	Low
Lab runway	7	84	− 1.4 (− 8.2 to 5.4)	↔	− 1	− 1	None	Low
All	15	145	− 5.0 (− 11.5 to 1.6)	↔	None	− 1	None	Low
*Stride frequency (strides/s)*								
Track	8	61	0.05 (− 0.02 to 0.12)	↔	− 1	− 1	None	Low
Lab runway	6	68	− 0.02 (− 0.07 to 0.03)	↔	− 1	None	− 1	Very low
All	16	133	0.02 (− 0.03 to 0.06)	↔	None	− 1	None	Low
Ankle and foot kinematic outcomes								
*Foot–ground angle at footstrike (°)*								
All	3	22	− 9.8 (− 13.1 to − 6.6)	↓	− 1	None	None	Low
*Ankle angle at footstrike (°)*								
Lab runway	4	43	− 1.9 (− 7.0 to 3.3)	↔	− 1	− 1	None	Low
All	5	55	− 2.3 (− 7.2 to 1.4)	↔	− 1	− 1	None	Low
*Peak ankle angle during stance (°)*								
Lab runway	4	70	− 0.6 (− 1.3 to 0.2)	↔	− 1	None	None	Low
*Ankle dorsiflexion ROM (°)*								
All	2	24	− 0.52 (− 12.1 to 11.0)	↔	− 1	− 1	None	Low
*Ankle in- and eversion a footstrike (°)*								
Lab runway	2	32	− 3.3 (− 8.4 to 1.8)	↔	− 1	− 1	None	Low
*Peak ankle in- and eversion during stance (°)*								
Lab runway	2	32	− 2.5 (− 9.1 to 4.0)		− 1	− 1	None	Low
*Ankle add- and abduction at footstrike (°)*								
Lab runway	2	32	1.0 (− 4.5 to 6.5)	↔	− 1	− 1	None	Low
*Peak ankle add- and abduction during stance (°)*								
Lab runway	2	32	0.45 (− 1.7 to 2.6)	↔	− 1	− 1	None	Low
Knee kinematic outcomes								
*Knee flexion at footstrike (°)*								
Lab runway	4	43	− 1.7 (− 3.7 to 0.4)	↔	− 1	None	− 1	Very low
All	7	65	− 2.3 (− 3.6 to − 1.1)	↑	− 1	None	None	Low
*Peak knee flexion during swing (°)*								
Lab runway	2	40	3.4 (− 0.8 to 7.5)	↔	− 1	None	− 1	Very low
All	4	50	1.2 (− 2.2 to 4.5)	↔	− 1	None	− 1	Very low
*Peak knee flexion during stance (°)*								
Lab runway	3	50	1.9 (− 0.8 to 4.5)	↔	− 1	− 1	None	Low
All	5	60	0.5 (− 1.8 to 2.8)	↔	− 1	− 1	None	Low
*Minimum knee flexion during gait cycle (°)*								
Lab runway	2	40	− 0.8 (− 2.7 to 1.2)	↔	− 1	None	− 1	Very low
*Knee flexion ROM (°)*								
All	2	24	6.3 (4.5 to 8.2)	↓	− 1	None	None	Low
*Knee angle toe-off (°)*								
All	4	21	− 0.7 (− 2.5 to 1.1)	↔	− 1	None	None	Low
*Knee add- and abduction at footstrike (°)*								
Lab runway	2	32	0.6 (− 1.7 to 2.9)	↔	− 1	− 1	None	Low
*Peak knee add- and abduction during stance (°)*								
Lab runway	2	32	0.3 (− 3.2 to 3.8)	↔	− 1	− 1	None	Low
*Peak knee add- and abduction at footstrike (°)*								
Lab runway	2	32	0.3 (− 3.2 to 3.8)	↔	− 1	− 1	None	Low
*Knee in- and external rotation at footstrike (°)*								
Lab runway	2	32	1.0 (− 1.7 to 3.6)	↔	− 1	None	None	Low
*Peak knee in- and external rotation during stance (°)*								
Lab runway	2	32	− 0.9 (− 3.1 to 1.2)	↔	− 1	None	None	Low
Hip and pelvis outcomes								
*Hip flexion at footstrike (°)*								
Lab runway	4	43	− 2.5 (− 7.4 to 2.4)	↔	− 1	− 1	− 1	Very low
All	5	53	− 2.7 (− 6.2 to 0.8)	↔	− 1	− 1	None	Low
*Peak hip flexion during stance (°)*								
Lab runway	2	32	− 6.5 (− 18.3 to 5.2)	↔	− 1	− 1	− 1	Very low
All	43	42	− 5.6 (− 12.3 to 1.1)	↔	− 1	− 1	− 1	Very low
*Peak hip flexion during gait cycle (°)*								
Track	3	20	− 3.2 (− 7.1 to 0.8)	↔	− 1	− 1	None	Low
All	4	40	− 2.2 (− 4.6 to 0.2)	↔	− 1	None	None	Low
*Hip ROM during stance (°)*								
Lab runway	2	32	− 9.0 (− 24.2 to 6.1)	↔	− 1	− 1	− 1	Very low
*Peak hip extension during gait cycle (°)*								
Track	3	20	3.6 (− 2.7 to 10.0)	↔	− 1	− 1	None	Low
All	4	30	2.8 (− 2.0 to 7.5)	↔	− 1	− 1	None	Low
*Hip angle at toe-off (°)*								
All	3	21	1.5 (− 6.5 to 3.6)	↔	− 1	− 1	None	Low
*Hip add- and abduction at footstrike (°)*								
Lab runway	2	32	0.75 (− 0.7 to 2.2)	↔	− 1	None	None	Low
All	3	42	0.6 (− 0.4 to 1.6)	↔	− 1	None	None	Low
*Peak hip add- and abduction during stance (°)*								
Lab runway	3	52	0.6 (− 0.5 to 1.8)	↔	− 1	None	− 1	Very low
All	4	62	0.6 (− 0.4 to 1.7)	↔	− 1	None	None	Low
*Vertical displacement (cm)*								
Lab runway	3	25	− 1.8 (− 3.99 to 0.03)	↔	− 1	− 1	None	Low
All	5	35	− 1.5 (− 2.7 to − 0.2)	↓	− 1	− 1	None	Low
Kinetic outcomes								
*Total foot pressure (SMD)*								
Track	2	18	− 1.25 (− 2.13 to − 0.37)	↔	− 1	− 1	None	Low
All	7	38	− 0.34 (− 0.89 to 0.21)	↔	− 1	− 1	None	Low
*Peak vertical ground reaction force (BW)*								
Lab runway	6	55	− 0.05 (− 0.11 to 0.01)	↔	− 1	None	− 1	Very low
*Average vertical loading rate (BW/s)*								
Lab runway	5	35	0.56 (− 4.7 to 5.8)	↔	− 1	None	None	Low
All	6	47	− 7.7 (− 24.0 to 8.6)	↔	− 1	− 1	None	Low
*Instantaneous vertical loading rate (BW/s)*								
Lab runway	5	35	5.8 (− 1.1 to 12.7)	↔	− 1	None	None	Low
*Transient peak (BW/s)*								
All	4	36	− 0.02 (− 0.16 to 0.12)	↔	− 1	− 1	None	Low
*Peak propulsive force (BW)*								
Lab runway	3	31	− 0.04 (− 0.06 to − 0.02)	↓	− 1	None	− 1	Very low
*Ankle joint moment (Nm/kg)*								
Lab runway	2	38	− 0.4 (− 0.7 to − 0.2)	↑	− 1	None	None	Low
*Knee joint moment (Nm/kg)*								
Lab runway	2	38	− 0.3 (− 1.0 to 0.4)	↔	− 1	− 1	None	Low
*Eccentric ankle power (W/kg)*								
Lab runway	2	38	− 2.3 (− 3.3 to 0.8)	↔	− 1	− 1	None	Low
*Peak tibial acceleration (g)*								
Track	2	24	− 4.2 (− 14.0 to 5.6)	↔	− 1	− 1	None	Low
Lab runway	2	27	0.01 (− 0.02 to 0.2)	↔	− 1	None	None	Low
All	7	60	− 0.8 (− 2.8 to 1.3)	↔	− 1	− 1	None	Low

Only outcomes with *k* > 1 are included in this table

*CI* confidence interval, *GRADE* Grading of Recommendations Assessment, Development and Evaluation, *k* number of outcomes, *n* number of participants, *ROM* range of motion, *SMD* standardized mean difference

MT running did not significantly affect stride time when compared to track or lab runway surfaces individually (Supplementary file IV) or when pooled across all surfaces (mean difference − 12.0; − 25.8 to 1.8; Table 2). MT running speed was no statistically significant moderator of the mean difference in stride time (Supplementary file IV). MT motor power, belt length and width were, however, statistically significant moderators of the mean difference in stride time, with more powerful motors leading to longer stride times and longer and wider belts leading to shorter MT stride times relative to overground (Supplementary file IV).

MT stride length was not significantly different compared to track or lab runway surfaces individually (Supplementary file IV) or across all overground surfaces (mean difference − 5.0 cm; − 11.5 to 1.6; Table 2). MT running speed, motor power and belt dimensions were not statistically significant moderators of the mean difference in stride length (Supplementary file IV).

MT stride frequency was not significantly different compared to track, concrete or lab runway surfaces individually (Supplementary file IV) or when pooled across surfaces (mean difference 0.02 strides/s; − 0.03 to 0.06; Table 2). MT running speed, motor power and belt dimensions were not statistically significant moderators of the mean difference in stride frequency (Supplementary file IV).

All individual spatiotemporal study results used and not used in meta-analysis are reported in Supplementary file III, Table SI.

### Ankle and Foot Kinematic Outcome Measures

**Fig. 5:**
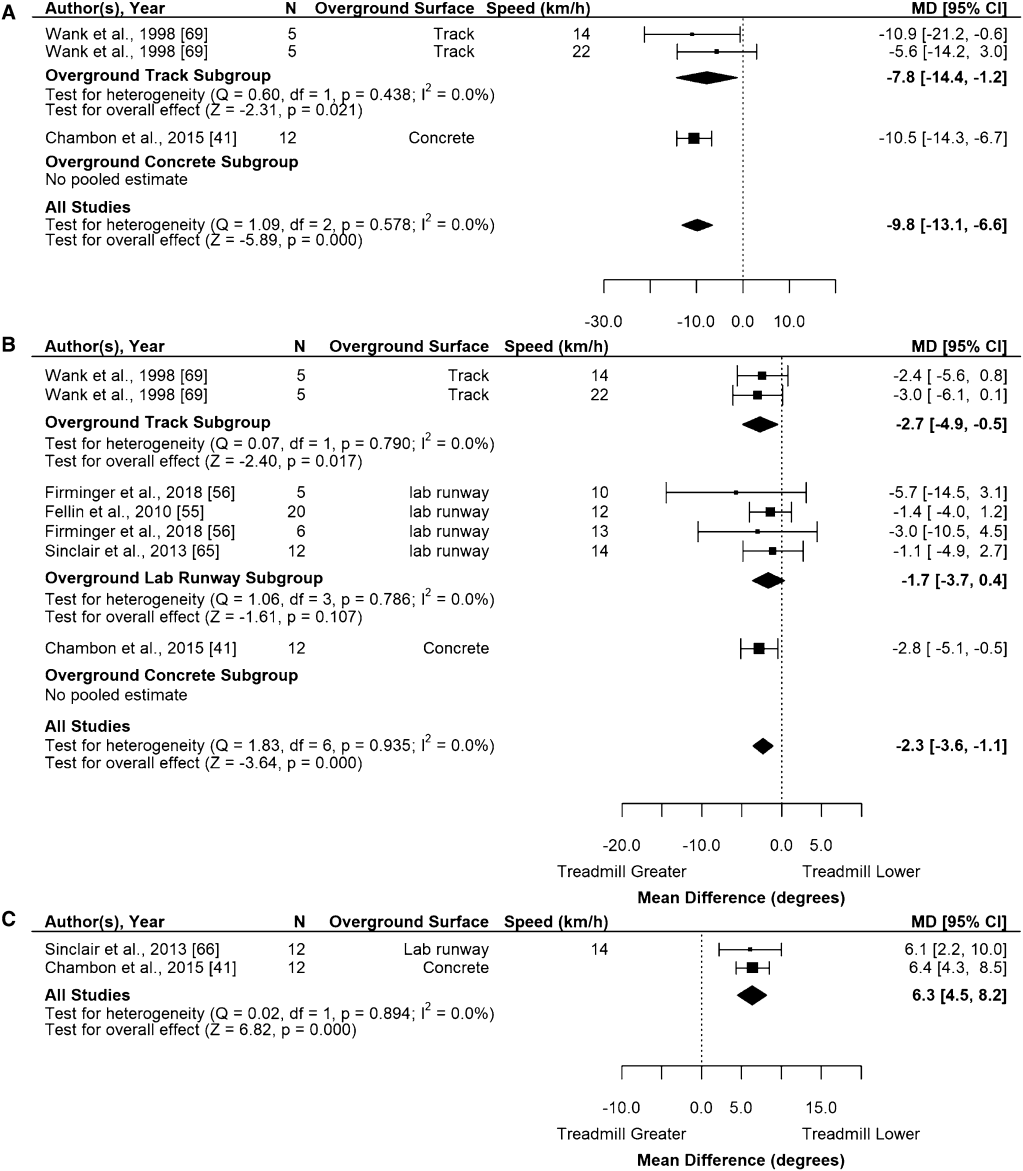
Random-effects meta-analysis of a sagittal foot–ground angle at footstrike, b knee flexion angle at footstrike and c knee flexion range of motion from footstrike to peak during stance during MT compared to overground running. Subgroup analysis based on overground surface with subgroups organized from least stiff surface to stiffest surface and studies organized from slowest to fastest speed. *CI* confidence interval, *df* degrees of freedom, *MD* mean difference, *N* sample size

Pooled results from one study indicated that MT sagittal foot-ground angle at footstrike was significantly lower (i.e., less inclined) than track foot-ground angle by − 7.8° (− 14.4 to − 1.2; *n* = 10; *k* = 2; *I*^2^ = 0%). Results from one study indicated that MT foot-ground angle at footstrike was significantly lower (i.e., less inclined) compared to concrete by − 10.5° (− 14.3 to − 6.7; *n* = 12; *k* = 1). When pooled across all surfaces, MT foot–ground angle at footstrike was significantly lower (less inclined) compared to overground foot angle by − 9.8° (− 13.1 to − 6.6; Table 2; Fig. 5a).

Results from one study indicated that MT ankle angle at footstrike was significantly lower (i.e., less dorsiflexed relative to neutral) compared to concrete by − 6.1° (− 9.6 to − 2.6; *n* = 12; *k* = 1). Pooled results from three studies indicated that MT ankle angle at footstrike was not significantly different from lab runway ankle angle (mean difference − 1.9°; − 7.0 to 3.3; *n* = 43; Table 2). When pooled across all surfaces, MT ankle angle at footstrike did not significantly differ from overground (mean difference − 2.3 = 9°; − 7.2 to 1.4; Table 2; Supplementary file IV).

MT peak sagittal ankle angle during stance was not significantly different from lab runway peak ankle angle (mean difference − 0.6°; − 1.3 to 0.2; Table 2; Supplementary file IV).

Results from one study indicated that MT ankle dorsiflexion range of motion was significantly higher (i.e., more dorsiflexion range of motion) when compared to concrete ankle dorsiflexion range of motion by 5.3° (2.5–8.1; *n* = 12; *k* = 1). Results from one other study, however, indicated that MT ankle dorsiflexion range of motion was significantly lower when compared to a lab runway by − 6.5° (− 10.4 to − 2.6; *n* = 12; *k* = 1). Pooled results across all surfaces showed no significant difference between MT and overground ankle dorsiflexion range of motion (mean difference − 0.52°; − 12.1 to 11.0; Table 2; Supplementary file IV).

MT ankle inversion and eversion at footstrike were not significantly different from a lab runway condition (mean difference − 3.3°; − 8.4 to 1.8; Table 2; Supplementary file IV).

Pooled results from two studies showed that MT peak ankle inversion and eversion during stance were not significantly different from a lab runway condition (mean difference − 2.5°; − 9.1 to 4.0; *n* = 32; *k* = 2; *I*^2^ = 85.7%; Table 2; Supplementary file IV).

MT ankle adduction and abduction at footstrike and the peak values during stance were not significantly different from a lab runway condition (mean difference 1.0°; − 4.5 to 6.5; Table 2; Supplementary file IV and mean difference 0.45°; − 1.7 to 2.6; Table 2, respectively).

All individual study results from all included studies that investigated ankle and foot kinematic outcomes used and not used in meta-analysis are reported in Supplementary file III, Table SII.

### Knee Kinematic Outcome Measures

Pooled results from one study show that MT knee flexion at footstrike was significantly higher (i.e., more flexed) than track knee flexion by − 2.7° (− 4.9 to − 0.5; *n* = 10; *k* = 2; *I*^2^ = 0%). Results from one study show that MT knee flexion at footstrike was significantly higher than concrete knee flexion by − 2.8° (− 5.1 to − 0.5; *n* = 12; *k* = 1). Pooled results from three studies show that MT knee flexion at footstrike was not significantly different from lab runway knee flexion (mean difference − 1.7°; − 3.7 to 0.4; *n* = 43; *k* = 4; *I*^2^ = 0%). When pooled across all surfaces, MT knee flexion at footstrike was significantly higher (i.e., more flexed) than overground knee flexion by − 2.3° (− 3.6 to − 1.1; Table 2; Fig. 5b). As heterogeneity was very low, meta-regression was not performed.

MT peak knee flexion during swing or the peak during stance was not significantly different compared to track or lab runway surfaces individually (Supplementary file IV) or when pooled across all surfaces (mean difference 1.2°; − 2.2 to 4.5; Table 2 and mean difference 0.5°; − 1.8 to 2.8; Table 2, respectively).

MT minimum knee flexion during the entire gait cycle was not significantly different from lab runway minimum knee flexion (mean difference − 0.8°; − 2.7 to 1.2; Table 2; Supplementary file IV).

MT knee flexion range of motion from footstrike to peak during stance was significantly smaller compared to track or lab runway surfaces individually (Fig. 5c and Supplementary file IV) or when compared to overground by 6.3° (4.5–8.2; Table 2).

MT knee angle at toe-off was not significantly different from track or lab runway surfaces individually (Supplementary file IV) or when pooled across surfaces (mean difference − 0.7°; − 2.5 to 1.1; Table 2).

MT knee adduction and abduction angle at footstrike and the peak values during stance were not significantly different from a lab runway condition (mean difference 0.6°; − 1.7 to 2.9; Table 2; Supplementary file IV and mean difference 0.3°; − 3.2 to 3.8; Table 2; Supplementary file IV, respectively).

MT knee internal and external rotation at footstrike and the peak values during stance were not significantly different from a lab runway condition (mean difference 1.0°; − 1.7 to 3.6; Table 2; Supplementary file IV and mean difference − 0.9°; − 3.1 to 1.2; Table 2; Supplementary file IV, respectively).

All individual study results from all included studies that investigated knee kinematic outcomes used and not used in meta-analysis are reported in Supplementary file III, Table SIII.

### Hip and Pelvic Kinematic Outcome Measures

Results from one study show a significantly less flexed hip at footstrike during MT running compared to track by − 4.1° (− 6.4 to − 1.8; *n* = 10; *k* = 1). MT hip flexion at footstrike was not significantly different from lab runway hip flexion (mean difference − 2.5°; − 7.4 to 2.4; Table 2) or the pooled effect across all surfaces (mean difference − 2.7°; − 6.2 to 0.8; Table 2; Supplementary file IV).

Results from one study show significantly less peak hip flexion during stance during MT compared to track by − 4.2° (− 6.3 to − 2.1; *n* = 10; *k* = 1). MT peak hip flexion during stance was not significantly different from lab runway hip flexion (mean difference − 6.5°; − 18.3 to 5.2; Table 2). When pooled across all surfaces, there were no significant differences between MT and overground running for peak hip flexion during stance (mean difference − 5.6°; − 12.3 to 1.1; Table 2; Supplementary file IV) or the entire gait cycle (mean difference − 2.2°; − 4.6 to 0.2; Table 2; Supplementary file IV).

MT hip range of motion during stance was not significantly different from lab runway hip range of motion (mean difference − 9.0°; − 24.2 to 6.1; Table 2; Supplementary file IV).

There were no significant differences in peak hip extension angle during the entire gait cycle for MT and over ground conditions when pooled across all surfaces (mean difference 2.8°; − 2.0 to 7.5; Table 2; Supplementary file IV) or when considering track or lab runway surfaces separately.

Results from one study indicated that MT hip angle at toe-off was significantly higher than track by − 6.1° (− 9.4 to − 2.8; *n* = 10; *k* = 1). MT hip angle at toe-off was not significantly different from lab runway (mean difference 1.2°; − 2.1 to 4.5; Table 2) or when pooled across all surfaces (mean difference − 1.5°; − 6.5 to 3.6; Table 2).

MT hip adduction and abduction at footstrike were not significantly different compared to track or lab runway surfaces individually or when pooled across all surface (mean difference 0.6°; − 0.4 to 1.6; Table 2; Supplementary file IV). MT peak hip adduction and abduction during stance were also not significantly different compared to track or lab runway surfaces individually or when pooled across surfaces (mean difference 0.6°; − 0.4 to 1.7; Table 2; Supplementary file IV).

**Fig. 6:**
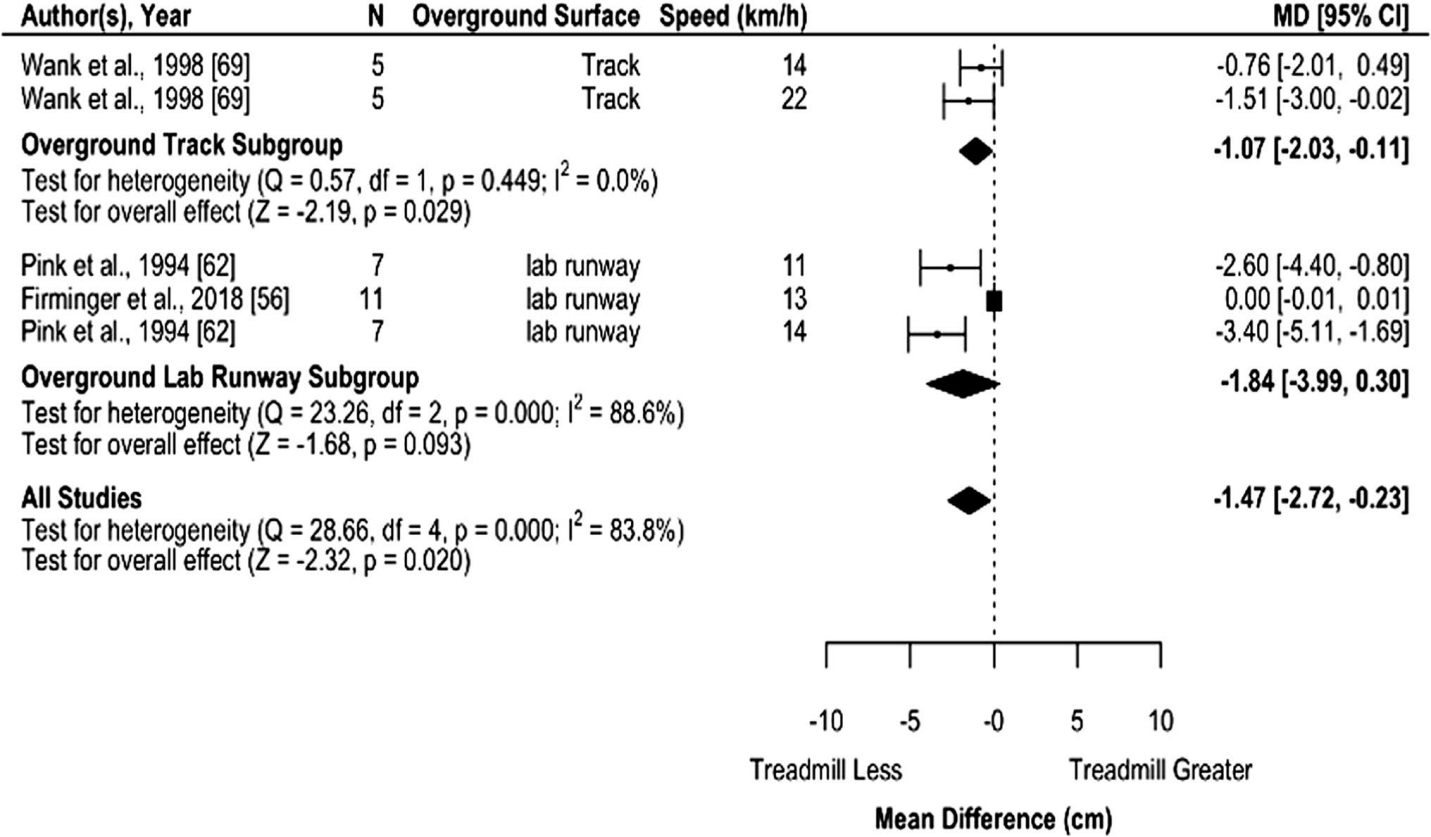
Random-effects meta-analysis of vertical displacement during MT compared to overground running. Subgroup analysis based on overground surface with subgroups organized from least stiff surface to stiffest surface and studies organized from slowest to fastest speed. *CI* confidence interval, *df* degrees of freedom, *MD* mean difference, *N* sample size

MT vertical displacement of pelvic markers/center of mass was significantly lower compared to track, but not lab runway surfaces individually (Fig. 6 and Supplementary file IV) and significantly lower when pooled across surfaces by − 1.47 cm (− 2.72 to − 0.23; Table 2).

All individual study results from all included studies that investigated hip and pelvis kinematic outcomes used and not used in meta-analysis are reported in Supplementary file III, Table SIV.

### Kinetic Outcome Measures

MT total foot peak pressure was significantly lower compared to track peak pressure by − 1.25 (Hedge’s *g*); − 2,13 to − 037; Table 2) but not concrete, lab runway or grass peak pressures (Supplementary file IV) or when pooled across all surfaces (− 0.34; − 0.89 to 0.21; Table 2).

MT peak vertical ground reaction force was not significantly different from lab runway peak vertical ground reaction force (mean difference − 0.05 BWs; − 0.11 to 0.01; Table 2; Supplementary file IV). MT running speed, motor power and belt dimensions were not statistically significant moderators of the mean difference in peak vertical ground reaction force (Supplementary file IV).

Results from one study show that MT average vertical loading rate is significantly lower compared to concrete by − 50 BW/s (− 64.5 to − 35.5; *n* = 12; *k* = 1). Average vertical loading rate did not significantly differ from lab runway (mean difference 0.56 BW/s; − 4.7 to 5.8; Table 2) or the pooled effect across surfaces (mean difference − 7.7 BW/s; − 24.0 to 8.6; Table 2; Supplementary file IV). MT instantaneous vertical loading rate did also not significantly differ from lab runway (mean difference 5.8 BW/s; − 1.1 to 12.7; Table 2; Supplementary file IV).

Results from one study indicated that MT impact transient was significantly lower than concrete transient peak by − 0.17 BW/s (− 0.28 to − 0.05; *n* = 12; *k* = 1; *I*^2^ = 0) and not significantly different from lab runway (mean difference 0.05 BW/s; − 0.06 to 0.17; Table 2) or when pooled across all surfaces, (mean difference − 0.02 BW/s; − 0.16 to 0.12; Table 2; Supplementary file IV).

Pooled results from two studies indicated that MT peak propulsive force was significantly lower than lab runway by − 0.04 BW (− 0.06 to − 0.02; Table 2; Supplementary file IV).

Pooled results from two studies indicated that MT ankle sagittal plane joint moment was significantly higher than lab runway ankle sagittal plane joint moment by − 0.4 Nm/kg (− 0.7 to − 0.2; Table 2; Supplementary file IV).

Pooled results from two studies indicated that MT knee sagittal plane joint moment was not significantly different from lab runway knee sagittal plane joint moment (mean difference − 0.3 Nm/kg; − 1.0 to 0.4; Table 2; Supplementary file IV).

Pooled results from two studies indicated that MT eccentric ankle power was not significantly different from lab runway eccentric ankle power (mean difference − 2.3 W/kg; − 3.3 to 0.8; Table 2; Supplementary file IV).

Pooled results indicated that MT peak positive tibial acceleration (i.e., tibial shock) was not significantly different compared to track, concrete, lab runway or grass surfaces individually (Supplementary file IV). When pooled across all surfaces, MT peak positive tibial acceleration was not significantly different from overground peak tibial acceleration with (mean difference − 0.8 g; − 2.8 to 1.3; Table 2) or without (mean difference 0.01 g; − 0.18 to 0.21; Table 2) the inclusion of one outlier study (Supplementary file III). MT speed, motor power and belt dimensions were not a statistically significant moderator of the mean difference in peak positive tibial acceleration both with and without outlier (Supplementary file IV).

All individual study results from all included studies that investigated kinetic outcomes used and not used in meta-analysis are reported in Supplementary file III, Table SV.

### Electromyography Outcome Measures

Results from all included studies that investigated electromyography outcomes are reported in Supplementary file III, Table SVI.

### Muscle–Tendon Unit and Bone Outcome Measures

Results from all included studies that investigated muscle–tendon unit or bone outcomes are reported in Supplementary file III, Table SVII. Briefly, muscle–tendon outcomes such as fascicle lengths and velocities were comparable between the two modes, while bone outcomes such as peak tibial axial compression strain and compression strain rate were lower during MT running.

## Discussion

Thirty-three studies comparing running biomechanics between MT and overground running with a total of 494 participants were included in this review. Considering the large number of outcome measures evaluated, the discussion focuses on outcome measures used in meta-analyses and additional outcome measures from individual studies that we considered most relevant for research, clinical practice or training. Potential reasons for biomechanical differences between both conditions, the implications of the findings and practical recommendations are discussed.

### Spatiotemporal Outcome Measures

Very low to low GRADE quality evidence indicated no differences in stride time, stride length and stride frequency. Low GRADE evidence indicated a 5-ms difference in ground contact time between MT and overground running. This difference may be too small to be of relevance for training, clinical practice and even research purposes because this is smaller than the minimum detectable change reported for contact time in several studies [11, 12]. Although these findings indicate that overall spatiotemporal measures do not differ between MT and overground running, there was notable inconsistency across individual studies, which sometimes reported significant differences between the two conditions (Supplementary file III). Inconsistent findings may result due to variation in the degree of comfort/familiarity with MT running, with a greater stride frequency and shorter stride length and contact time reported in individuals who are less comfortable [24]. Meta-regression showed a significant association between treadmill motor power, belt length and belt width and stride time, with a higher motor power being associated with longer MT stride times, and longer and wider belts being associated with shorter MT stride times relative to overground (Appendix IV in ESM). These findings likely represent type I errors for the following reason: a large number of studies investigated contact time and other outcomes such as stride length and frequency, but we found no significant association in the meta-regression with these outcomes and motor power or belt dimensions. Only four studies investigated stride time, while also providing information on motor power or belt dimensions and we, therefore, suspect these analyses to be more prone to co-variation of other variables and inferential errors and thus to represent a false positive finding given the non-significant effects with a larger number of studies on other outcomes. Further, the magnitude of the differences are likely trivial to small, since a one-meter increase in belt length or width would be required to decrease stride time by 22 ms and 70.2 ms relative to overground, respectively. Similarly, a one-kW increase in motor power is associated with an increase of only 2.88 ms in stride time relative to overground.

### Kinematic Outcome Measures

Overall, MT running kinematics are largely comparable to overground running kinematics, particularly in the frontal and transverse planes, although less studies investigated these outcomes. Nevertheless, some differences were observed for sagittal plane kinematics, particularly at footstrike.

Low GRADE evidence indicates ~ 10° lower foot-ground angle at footstrike (i.e., the angle between the shoe and ground) when running on a MT compared to overground. This finding remained when analysis was limited to concrete overground surfaces, but not lab runway surfaces. Similarly, subgroup analysis showed a larger ankle dorsiflexion range of motion during MT running when compared to concrete, but a smaller range of motion compared to a lab runway, resulting in no significant difference when pooled across surfaces. During MT running, the knee was also more flexed at footstrike by ~ 2° when pooled across all surfaces and track and concrete overground surfaces individually, but not lab runway surfaces. Further, MT knee flexion range of motion from footstrike to peak during stance was significantly smaller compared to concrete, lab runway and the combination of both surfaces by ~ 6°, likely because the knee was already placed in a more flexed position at footstrike during MT running. Similar findings were reported by a study published after the completion of the meta-analyses [70]. Hip flexion at footstrike was, however smaller (i.e., less flexed) when compared to track by ~ 4°. Further, MT peak hip flexion during stance was lower compared to track, but not lab runway and the hip was more extended at toe-off during MT running compared to track but not lab runway surfaces.

Similar to the spatiotemporal differences, some of the statistically significant kinematic differences between MT and overground running may be too small to be of practical relevance when considered in isolation. For example, the ~ 4° lower peak hip flexion during MT running is smaller than the standard error of measurement with manual marker placement [71]. Similarly, the ~ 2° larger knee flexion at footstrike in overground running is smaller than the smallest detectable difference with two-dimensional motion analysis [72]. However, other outcomes such as the foot-ground angle and ankle angle at footstrike are larger than the smallest detectable change reported for this outcome [72]. Although some kinematic differences may therefore be too small to be relevant when considered in isolation, their combined effect may be relevant and primarily reflect a strategy to compensate for differences in surface stiffness between the two conditions, although none of the studies actually reported surface stiffness. Specifically, it has been suggested that increases in knee flexion and ankle dorsiflexion at footstrike and increases in their peak values during stance are strategies to reduce lower extremity stiffness, which in turn compensates for increases in surface stiffness [73]. Indeed, increases in knee and hip angle at initial contact (i.e., a more flexed leg) have been observed with increases in surface stiffness [74, 75]. The increased knee flexion and smaller foot angle at initial contact during MT running could therefore reflect a compensatory strategy to reduce lower extremity stiffness when running on a stiffer MT running surface compared to a more compliant overground surface. Interestingly, these findings contrast with the findings of a recent study that found a lower surface stiffness in a treadmill compared to both concrete and tartan (track) overground surfaces [76]. Differences in surface stiffness between different treadmills and overground surfaces may explain these conflicting findings.

MT running vertical displacement during the entire gait cycle was significantly lower by ~ 1.5 cm when compared against the pooled effect of all overground surfaces, or track or lab runway separately. Similar findings have been reported by a study among three athletes not included in this review [77]. This difference is larger than the typical inter-trial variability in vertical displacement [78] and comparable to the difference in vertical displacement reported between highly trained, well trained and non-trained runners [79], suggesting it may be of practical relevance. The smaller vertical displacement may be a consequence of a higher stride frequency in MT running in this small sub-set of studies, as one of the studies that reported a smaller vertical displacement also reported a significantly higher stride frequency during MT running [69]. The higher stride frequency may again reflect insufficient familiarization/comfort with MT running in these studies [24]. In support of this, all studies that measured vertical displacement were of high risk of bias for providing insufficient familiarization. The higher hip extension at toe-off during MT running compared to track running found in subgroup analysis could be due to intra-belt speed variations [15, 19]. Specifically, the decreasing vertical friction force and increasing propulsive forces will accelerate the belt at the end of the stance phase and this can drag the hip joint into further extension at toe-off. This effect may however only occur in MTs with a less powerful motor, lightweight roller/flywheel or slow belt speed update frequency [15, 81, 82] and differences in these aspects between studies may explain the conflicting findings.

### Kinetic Outcome Measures

Meta-analyses provided very low to low GRADE quality evidence that MT most kinetic outcomes were not different from overground. Conflicting findings were found for total foot pressure, average vertical loading rate and transient peak, with subgroup analysis showing MT total foot pressure, average vertical loading rate and transient peak to be lower compared to running on track for foot pressure or concrete for loading rates and transient peaks. Meta-analyses further provided very low to low GRADE quality evidence that MT peak propulsive force was lower and ankle sagittal plane joint moment was significantly higher compared to overground (lab runway) surfaces.

The differences in some of the kinetic outcome measures result from various aspects. First, it is often believed that instrumented MTs cannot measure vertical and horizontal forces as accurate as a force platform. Using a simplified model of an instrumented MT, Willems, Gosseye [81] however mathematically demonstrated that both vertical and horizontal forces applied to the MT belt can be measured accurately by force sensors mounted under the MT, although compliance in the mechanical model can introduce some biomechanical differences [20, 28, 81–83]. Related to this, several studies have showed reduced plantar pressure when running on more compliant surfaces [27, 84], suggesting the decreased plantar force in MT running compared to running on a track found in this review could be due to differences in surface stiffness. However, no differences were found when comparing MT to concrete or lab runway surfaces, potentially due to the smaller groups and hence lack of statistical power. Peak vertical ground reaction force has been shown to remain constant within the range of surface stiffness likely used in the included studies due to changes in lower extremity stiffness [73, 74, 80]. Therefore, the altered sagittal plane kinematics in MT running likely partly compensated for the reduced surface stiffness, resulting in no significant difference in vertical peak ground reaction forces between MT and overground running. Loading rates have however found to be higher with increases in surface stiffness [73] and the higher surface stiffness of concrete compared to the MTs therefore likely partly explains the lower loading rate and transient peak found in MT running in this review. Finally, the reduced propulsive force during MT could be due to several reasons. First, a lack of air resistance during MT running reduces propulsion requirements, but this effect is expected to be negligible at the relatively low speeds investigated (10–13.7 km/h). Further, belt speed was relatively stable when peak propulsive force was exerted [63] and intra-belt speed fluctuations are therefore also unlikely to explain this effect. Rather, the authors suggested that the reduced peak propulsive force was due to insufficient familiarization (~ 5 min). Indeed, insufficient familiarization/comfort with MT running and perception differences can result in a higher stride frequency, and hence shorter stride length during MT running [24, 85]. The shorter stride length in turn reduces braking forces [86] and hence also requires less propulsive forces to maintain speed. Indeed, one study also reported significantly lower braking forces during MT running [56].

### Electromyographic Outcome Measures

No meta-analysis was performed for electromyography outcome measures as the outcomes measures were mostly expressed in different units, normalized using different procedures, or only reported for a specific phase. Further, several studies did also not report sufficient data to calculate (standardized) mean differences. With regard to the qualitative findings, the timing of muscle onset, offset, time of maximum muscle activity, and co-contractions tended to be similar between MT and overground running (Supplementary file III). Some differences were however reported in the amplitude of muscle activity.

It is often believed that MT running requires less propulsion as the MT moves the legs below the body, while in overground running the body needs to move over the legs. It has for example been suggested that whilst an explicit push-off is required in overground running, the leg only needs to be lifted at the end of stance in MT running, resulting in reduced soleus activity in MT running [51]. However, the evidence for this is conflicting, with one study reporting no significant difference in soleus muscle activity [69], one study reporting higher and lower activity in the weight acceptance and push-off phase respectively [51], and two other studies reporting significantly lower activity in MT running [50, 61]. Similarly, it has been suggested that the hamstrings are used to a greater extent in overground than MT running to produce propulsive forces [87]. However, the evidence is also conflicting with most studies reported no significant difference in hamstrings muscle activity [50, 61, 69], one study reporting lower activity during the stance phase only [68], one study reporting lower activity during the first 50% of the stance phase only [65], and another study reporting lower hamstring activity during the whole gait cycle [64]. Nevertheless, van Ingen Schenau [18] mathematically demonstrated MT running to be mechanically comparable to overground running when belt speed is constant and air drag is negligible. Both conditions therefore require equal propulsion of the body if these assumptions are met. Studies investigating muscle activity have been performed at relatively slow speeds (10.8–15.4 km/h), making the lack of air drag [21] unlikely to primarily contribute to these differences. A higher surface stiffness has however been shown to induce higher muscle activity in several muscles [68, 88]. Of the seven studies investigating differences in the amplitude of muscle activation, two studies used track as an overground surface [51, 69], three studies used a lab runway [50, 61, 65], one study used concrete [64], and one used a combination of different surfaces [68]. Most studies that reported lower muscle activity in MT running used a relatively stiff overground running surface such as concrete or a lab runway and this could therefore (partly) explain the potential for lower muscle activity in the MT condition in some but not all studies. Although this would be in line with a lower surface stiffness in a MT compared to concrete and tartan surfaces observed in a recent study [76], this would be in contrast to the findings of the kinematic differences discussed before, which suggest MT are often stiffer than the overground surfaces. A final explanation for the lower muscle activity could be the reduced vertical displacement of the center of mass [51] found in some studies. Specifically, a reduced vertical displacement will require less acceleration in the vertical direction, and it follows from Newton’s second law; force = mass × acceleration, that this reduced vertical acceleration will reduce total vertical forces when body mass remains equal. These lower forces in turn require less muscle activation.

These findings collectively indicate that the majority of electromyography outcome measures do not significantly differ between MT and overground running, but also that some muscles are activated to a lower extent during MT compared to overground running.

### Implications for Training, Research and Clinical Practice

**Fig. 7:**
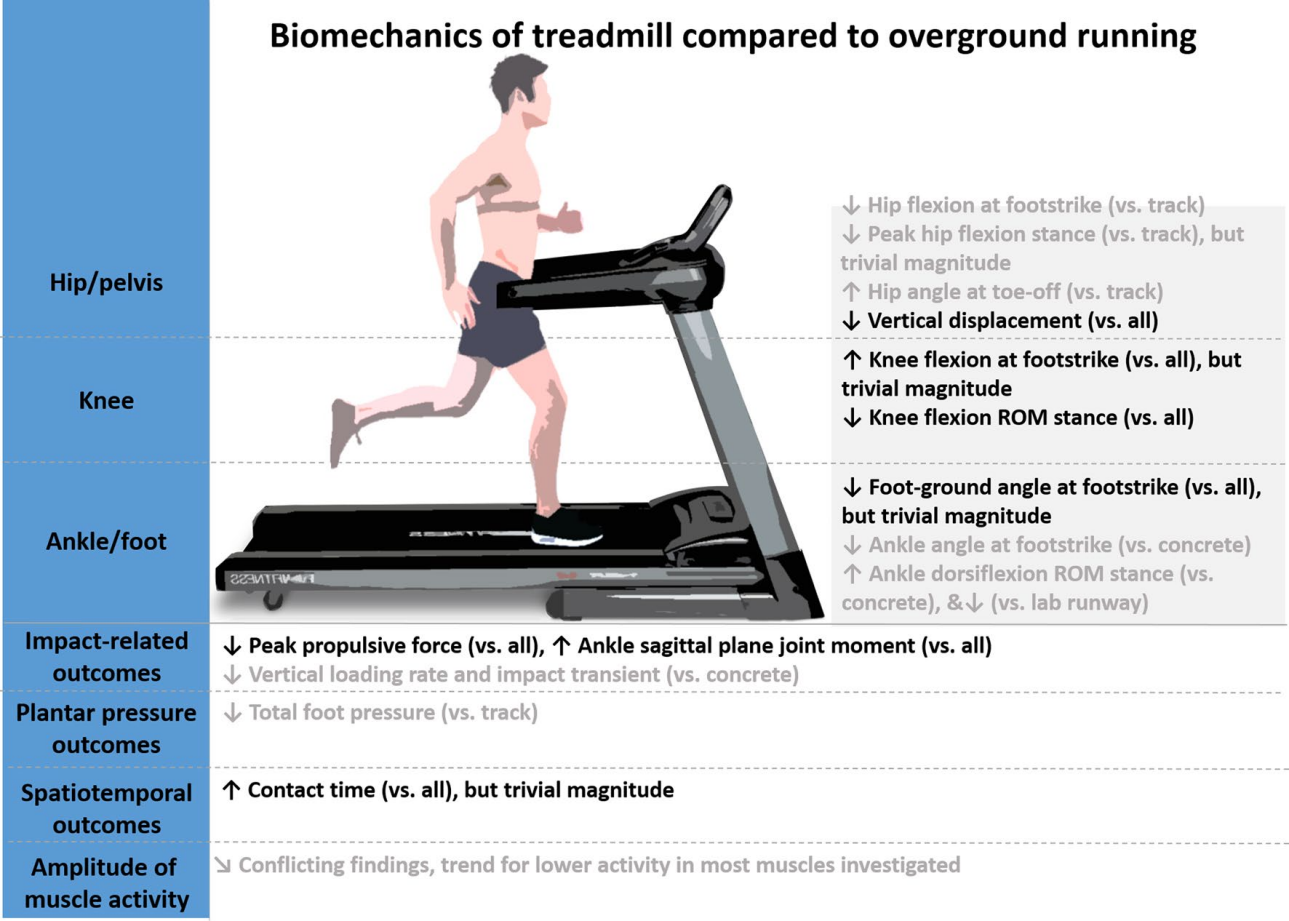
Summary of significant differences between treadmill and overground running biomechanics found with meta-analyses. Overall surface effects are indicated in black, subgroup (individual surface) effects in gray. Amplitude of muscle activity represents a qualitative interpretation of the findings as meta-analysis could not be performed for this outcome. *ROM* range of motion. Treadmill; “down arrow” lower; “up arrow”, greater/longer compared to overground

Overall, the findings of this review indicate that the biomechanics of MT running are largely comparable to overground running, with most outcomes not being significantly different, and some outcomes being significantly different but of trivial magnitude. However, some outcomes differ significantly and with substantial magnitude to potentially impact training, research and clinical practice. Figure 7 summarizes the most important findings of this review. Since researchers, clinicians and athletes often aim to use a MT to simulate overground training conditions as closely as possible, we provide several suggestions on how to minimize biomechanical differences between the two conditions and we also discuss the implications of the biomechanical differences found in this review.

First, differences in surface stiffness can affect running biomechanics and are likely partly responsible for the reported biomechanical differences between MT and overground running. MT surface stiffness should therefore be matched as closely as possible to the specific overground surface to improve generalizability of results. Since most runners run on concrete [1, 39], researchers, but also clinicians and athletes should attempt to use MTs that mimic the surface stiffness of concrete rather than a lab runway to mimic overground running conditions as closely as possible. Despite the importance of this aspect, none of the included studies reported the surface stiffness of the MT or overground condition and these could also not be derived from the MT manuals. We therefore urge future research to assess surface stiffness (see Colino et al. [89] for a standardized test) and match surface stiffness between the two conditions.

Second, intra-belt speed variations have been shown to affect running biomechanics [15] and these can also contribute to biomechanical differences. Intra-belt speed variations can result from inadequate motor power, too low belt speed update frequency or slip of the belt over the drivers [82]. The motor power and update frequency required to minimize biomechanical differences depends on factors such as the weight of the subject and the running speed, with heavier subjects and higher running speeds resulting in higher friction and braking forces and hence higher intra-belt speed variations. These higher intra-belt speed variations could in turn contribute to larger biomechanical differences observed at higher running speeds in some studies [13]. A high motor power and belt speed update frequency are therefore required to minimize biomechanical differences, particularly for heavier individuals and at higher running speeds. Meta-regression analyses did however not provide sufficient information on the required motor power to minimize differences. Nevertheless, lower quality commercial MTs usually have a lower motor power and belt speed update frequency and care should therefore be taken with generalizing the findings of biomechanical data collected in these MTs to overground running. Further, although intra-belt speed variations likely increase with increases in running speed due to the higher braking and propulsion forces, meta-regression showed no significant associations of running speed with any outcome.

Third, the degree of familiarization or comfort with MT running can affect MT running biomechanics. Several studies have investigated how much familiarization is required to achieve stable MT running biomechanics within one session. Although estimates vary considerably from 30 s [90] up to 9 min [23, 24, 91, 92], most studies report ~ 8 min [24, 91]. However, substantial individual differences have been reported and some individuals may therefore require considerably more or less familiarization [93]. Despite the importance of sufficient familiarization, only few studies reported the familiarization period or, if reported, provided sufficient familiarization prior to data collection (Table 1). Therefore, insufficient familiarization may also have contributed to some of the biomechanical differences and we suggest adopting at least 8 min of familiarization in novice MT runners before each condition and check their comfort prior to MT tests to minimize biomechanical differences.

Fourth, perceptual differences may also influence MT running biomechanics and should therefore be matched between both conditions. Specifically, it has been shown that individuals perceive MT running speed as faster than overground running speed [30] and the higher perceived MT running speed may result in higher stride frequencies and shorter stride lengths compared to overground running [85].

In some situations, the subtle differences in MT running biomechanics could be useful for training and rehabilitation. MTs with a less stiff surface may for example be preferable in rehabilitation settings as this will reduce vertical loading rates and transient peaks compared to stiff overground surfaces, such as concrete, as indicated by this review. It is however important to realize that this will also alter kinematics and muscle activation, hereby potentially changing the training stimulus and leading to mode-specific adaptations [94]. Further, there is likely a tradeoff between lower impacts in MT running, but also more regular stride dynamics [59] that will result in the same tissue being subjected to repetitive loading, which may in turn increase injury risk [95]. Similarly, although bone compression and strains, measured via an implanted bone strain gauge, [6] and plantar fascia strains [67] have been found to be lower in MT running, peak forces and loading rates on the Achilles tendon have been shown to be higher during MT running [16]. In line with this, a study published after completion of the meta-analysis also found higher muscle forces in the gastrocnemius and soleus during MT running [70]. MT running may therefore be suitable for rehabilitation from lower limb stress fractures, but not Achilles tendinopathy, Achilles ruptures or calf muscle strains. Finally, our previous systematic review found reduced endurance performance and no significant difference in oxygen uptake between non-inclined MT and overground running at speeds < 18 km/h [30]. The absence of air resistance in MT running reduces oxygen uptake and theoretically improves MT performance compared to overground running. In addition to a lack of comfort and lack of sweat evaporation and hence thermoregulation that can explain these differences [30], the findings of the current review suggest that biomechanical differences may also contribute to a higher energy cost and hence reduced running performance during MT compared to overground running. Specifically, numerous studies have found that modifications of running technique acutely decrease running economy [96–99]. Since some individuals -particularly individuals that are uncomfortable with MT running- show differences in their running technique during MT running, this may increase energy cost and hence partly mask the lack of air resistance, particularly at lower running speeds.

## Limitations and Future Directions

There are several limitations to this review that should be considered when interpreting the findings. First, most studies compared MT running to running in a lab runway, which does not necessarily reflect the concrete running surface, where most runners run [1, 39]. The findings of several subgroup analyses suggest that the overground surface used affects the biomechanical differences between MT and overground running and the current findings may, therefore, underestimate the actual biomechanical differences. Related to this, several studies used relatively high-quality MTs and biomechanical differences may be smaller and hence underestimated in these MTs [7, 14, 15, 27], although these findings could not be confirmed in the meta-regression with MT motor power and belt dimensions as co-variates. Nevertheless, these findings indicate that care should be taken with generalizing MT running biomechanics to overground running. Second, this review was restricted to non-incline, shod, non-fatigued motorized MT and constant-velocity running below 25 km/h in healthy adults. Biomechanical differences are likely larger when accelerating [100, 101] and when running at higher speeds (i.e., sprinting) on regular MT’s [17, 21] and may also be impacted by the use of shoes [41] and fatigue status [57, 58] and the findings of this review can therefore not be generalized to these conditions. Indeed, special MTs have been developed for sprinting that may reduce biomechanical differences [102–104]. Third, most meta-analyses were affected by high levels of heterogeneity. Although we attempted to explore the causes of the heterogeneity by performing sub-group analysis based on overground surface and meta-regression based on running speed, MT motor power and belt dimensions when sufficient studies were available, other factors that were not investigated such as running shoes used and footstrike pattern may also contribute to the high heterogeneity. Indeed, we could not include MT running experience or familiarization as a subgroup or in meta-regression because most studies did not clearly specify the prior experience of the participants with MT running, even though this is likely to affect the differences between the two modes [23, 24, 91, 92]. Similarly, most studies did not specify the running shoes and footstrike pattern used. Fourth, some studies did not report all information required for meta-analysis and we therefore extracted the required information from figures or estimated the information based on other studies. This likely introduced some error and we therefore urge researchers to improve the reporting and provide open data. In line with these suggestions, we have provided all data extracted or provided by authors in the supplementary file to facilitate further research. Finally, the estimated correlation coefficient used for computation of the variance was often implausible (e.g., > 1), likely because Bonferroni-corrected p-values were reported. This necessitated the use of a default correlation coefficient of 0.50 for all studies which could cause underestimation of the actual differences between MT and overground running.

## Conclusion

Overall, the findings indicate that MT running biomechanics are largely comparable to overground running biomechanics, but nevertheless differ on several aspects. These differences likely result from (1) differences in MT and overground surface stiffness, (2) insufficient MT running experience and comfort, (3) insufficient MT motor power, restricting belt dimensions and a compliant mechanical model, (4) differences in air resistance at higher running speeds, and (5) altered speed perception. Researchers, clinicians and athletes should therefore take these factors into consideration to minimize biomechanical differences between MT and overground running. Minimizing these biomechanics differences can in turn improve the generalizability of research and clinical gait analysis and improve the transfer of training.

## Electronic supplementary material

Below is the link to the electronic supplementary material.

Supplementary material 1 (PDF 190 kb)

Supplementary material 2 (PDF 460 kb)

Supplementary material 3 (PDF 776 kb)

Supplementary material 4 (PDF 410 kb)

Supplementary material 5 (XLSX 70 kb)

## Data Availability

The datasets generated and/or analyzed during the current systematic review are available in Electronic Supplementary Material file III and V.
