# Supplementary material for: Is Motorized Treadmill Running Biomechanically Comparable to Overground Running? A Systematic Review and Meta-Analysis of Cross-Over Studies
Source: Sports Med. 2019 Dec 4;50(4):785–813. doi: 10.1007/s40279-019-01237-z (PMC7069922; doi:10.1007/s40279-019-01237-z)
Supplement: Supplementary file 1 — Supplementary material 1 (PDF 190 kb) [file 40279_2019_1237_MOESM1_ESM.pdf]

Van Hooren et al. (2019). Biomechanical comparison of treadmill and overground running. Sports Medicine.
Email corresponding author: basvanhooren@hotmail.com. Department of Nutrition and Movement Sciences, NUTRIM School of Nutrition and Translational Research in Metabolism, Maastricht University Medical Centre+, Maastricht, The Netherlands

Supplementary file I: Systematic search string used in MEDLINE/PubMed

The following combination of subject headings, keywords and Booleans was used for the systematic search in MEDLINE/PubMed: (("Running"[MeSH] OR "Jogging"[MeSH] OR Running[tiab] OR Run[tiab] OR Runner[tiab] OR Runners[tiab] OR Jogging[tiab] OR Jog[tiab] OR Jogger[tiab] OR Joggers[tiab] OR Sprinting[tiab] OR Sprint[tiab] OR Sprinter[tiab] OR Sprinters[tiab] OR Running[ot] OR Run[ot] OR Runner[ot] OR Runners[ot] OR Jogging[ot] OR Jog[ot] OR Jogger[ot] OR Sprinting[ot] OR Sprint[ot] OR Sprinter[ot] OR Sprinters[ot]) AND (Treadmill[tiab] OR Treadmills[tiab] OR "Alter G"[tiab] OR "Pulsar"[tiab] OR "ActiveStep"[tiab] OR treadmill[ot])) AND ("Track and Field"[MeSH] OR Overground[tiab] OR Track[tiab] OR Field[tiab] OR "In-situ"[tiab] OR "over ground"[tiab] OR Floor[tiab] OR Road[tiab] OR Overground[ot] OR Track[ot] OR Field[ot] OR "In-situ"[ot] OR "over ground"[ot] OR Floor[ot] OR Road[ot]) NOT ("animals"[MeSH Terms] NOT "humans"[MeSH Terms]).
