# Supplementary material for: Is Motorized Treadmill Running Biomechanically Comparable to Overground Running? A Systematic Review and Meta-Analysis of Cross-Over Studies
Source: Sports Med. 2019 Dec 4;50(4):785–813. doi: 10.1007/s40279-019-01237-z (PMC7069922; doi:10.1007/s40279-019-01237-z)
Supplement: Supplementary file 2 — Supplementary material 2 (PDF 460 kb) [file 40279_2019_1237_MOESM2_ESM.pdf]

Email corresponding author: basvanhooren@hotmail.com. Department of Nutrition and Movement Sciences, NUTRIM School of Nutrition and Translational Research in Metabolism, Maastricht University Medical Centre+, Maastricht, The Netherlands

Supplementary file II Extended risk of bias information

The Cochrane Collaboration's tool's performance bias criterion was removed because it was considered impossible to blind participants to running conditions. Criteria related to statistical bias, familiarization bias and intensity bias were added as these were considered important contributors to risk of bias. Statistical bias related to small sample sizes and whether the included studies used appropriate statistical analysis for paired data from a cross-over design. Familiarization bias related to whether the participants were adequately familiarized (> 6min [23]) with the MT condition. Intensity bias related to whether included studies effectively matched the speed and surface stiffness between MT and overground running conditions and was a qualitative assessment based on length of the overground runway, the method by which speed was monitored during the overground trials, whether or not MT speed was calibrated and match of surface stiffness and running shoes. Specifically, if overground conditions involved a short runway (e.g., <25 m when the running speed was > 15 km/h) there was a chance that the participants were still accelerating or already decelerating during the trial, herby potentially inducing bias. Second, we considered timing gates or markers on the pelvis as methods with low risk of bias to control overground speed, but if the range in speed allowed during trials was >5% from the target speed this was considered as a source of bias. Third, if authors did not mention whether MT speed was calibrated prior to data collection this may also have induced bias. Fourth, if authors did not clearly state the surface of overground conditions, bias may have risen from the differences in surface stiffness.
