# Supplementary material for: Is Motorized Treadmill Running Biomechanically Comparable to Overground Running? A Systematic Review and Meta-Analysis of Cross-Over Studies
Source: Sports Med. 2019 Dec 4;50(4):785–813. doi: 10.1007/s40279-019-01237-z (PMC7069922; doi:10.1007/s40279-019-01237-z)
Supplement: Supplementary file 3 — Supplementary material 3 (PDF 776 kb) [file 40279_2019_1237_MOESM3_ESM.pdf]

Van Hooren et al. (2019). Biomechanical comparison of treadmill and overground running. Sports Medicine.
Email corresponding author: basvanhooren@hotmail.com. Department of Nutrition and Movement Sciences, NUTRIM School of Nutrition and Translational Research in Metabolism, Maastricht University Medical Centre+, Maastricht, The Netherlands

Table SI. Spatiotemporal outcome measures

Study	Treadmill mean ± SD	Overground mean ± SD	P-value (when available); arrow indicating direction of effect compared to overground
		**Ground contact time (ms)**	
Baur et al. [51]	212 ± 33	200 ± 39	n.s.; ↔
Chambon et al. [41]	Shoe with 0 mm drop: 297 ± 17<br>Shoe with 4 mm drop: 293 ± 18<br>Shoe with 8 mmm drop: 299 ± 16	Shoe with 0 mm drop: 281 ± 20<br>Shoe with 4 mm drop: 284 ± 27<br>Shoe with 8 mm drop: 286 ± 22	-<br>-<br>-
Cronin, Finni [54]	303 ± 28	293 ± 30	n.s.; ↔
Elliott, Blanksby [13]	Males 3.7 m/s: 233 ± 17<br>Males 5.41 m/s: 181 ± 11<br>Females 3.97 m/s: 211 ± 27<br>Females 5.29 m/s: 167 ± 11	Males 3.7 m/s: 227 ± 14<br>Males 5.41 m/s: 179 ± 13<br>Females 3.97 m/s: 212 ± 29<br>Females 5.29 m/s: 173 ± 15	$p = 0.14$; ↔<br>$p = 0.53$; ↔<br>$p = 0.73$; ↔<br>$p = 0.19$; ↔
Fu et al. [7]	Non-cushioned TM: 264 ± 5.8<br>Cushioned TM: 262 ± 6.1	Concrete: 272.6 ± 18.5<br>Synthetic track: 266.7 ± 28.9<br>Grass: 265.5 ± 8.7	Cushioned TM vs concrete: $p = 0.045$; ↓<br>All other comparisons: n.s.; ↔
Garcia-Perez et al. [57]	3.33 m/s: 252 ± 5<br>4 m/s: 233 ± 5	3.33 m/s: 234 ± 8<br>4 m/s: 212 ± 9	$p = 0.035$; ↑<br>$p = 0.017$; ↑
Hong et al. [27]	 Total foot: 221 ± 13.9<br>Medial head: 92.2 ± 14.5<br>Lateral heel 90.8 ± 14.7<br>Medial midfoot: 83.6 ± 14.6<br>Lateral midfoot: 118.4 ± 14.6<br>Medial forefoot: 160.0 ± 9.7<br>Central forefoot: 168.5 ± 21.1<br>Lateral forefoot: 150.7 ± 21.9<br>Greater toe: 161.1 ± 13.9<br>Lesser toes: 159.5 ± 13.2	Concrete<br>Total foot: 206.3 ± 23.4<br>Medial head: 82.1 ± 25.4<br>Lateral heel: 78.2 ± 25.5<br>Medial midfoot: 73.2 ± 22.4<br>Lateral midfoot: 113.1 ± 39.4<br>Medial forefoot: 157.8 ± 30.4<br>Central forefoot: 165.0 ± 29.2<br>Lateral forefoot: 155.7 ± 31.2<br>Greater toe: 140.3 ± 18.6<br>Lesser toe: 143.6 ± 17.7	 n.s.; ↔<br>n.s.; ↔<br>n.s.; ↔<br>n.s.; ↔<br>n.s.; ↔<br>n.s.; ↔<br>n.s.; ↔<br>n.s.; ↔<br>$p = 0.002$; ↑<br>$p = 0.002$; ↑

Van Hooren et al. (2019). Biomechanical comparison of treadmill and overground running. Sports Medicine.
Email corresponding author: basvanhooren@hotmail.com. Department of Nutrition and Movement Sciences, NUTRIM School of Nutrition and Translational Research in Metabolism, Maastricht University Medical Centre+, Maastricht, The Netherlands

		Natural grass<br>Total foot: 211.9 ± 21.4<br>Medial head: 86.8 ± 16.9<br>Lateral heel: 83.5 ± 20.7<br>Medial midfoot: 80.1 ± 20.1<br>Lateral midfoot: 111.9 ± 28<br>Medial forefoot: 166.1 ± 20<br>Central forefoot: 171 ± 20<br>Lateral forefoot: 160.2 ± 24.1<br>Greater toe: 143.9 ± 17.6<br>Lesser toe: 145.1 ± 18.6	 n.s.; ↔<br>n.s.; ↔<br>n.s.; ↔<br>n.s.; ↔<br>n.s.; ↔<br>n.s.; ↔<br>n.s.; ↔<br>n.s.; ↔<br>*p* = 0.002; ↑<br>*p* = 0.002; ↑
Kluitenberg et al. [40]	HS runners slow speed: 254 ± 21.13<br>HS runners preferred speed: 232 ± 20.49<br>HS runners fast speed: 220 ± 21.14<br>NHS runners slow speed: 237 ± 19<br>NHS runners preferred speed: 222 ± 12<br>NHS runners fast speed: 206 ± 12	HS runners slow speed: 258 ± 22.00<br>HS runners preferred speed: 232 ± 23.34<br>HS runners fast speed: 223 ± 21<br>NHS runners slow speed: 240 ± 17<br>NHS runners preferred speed: 229 ± 12<br>NHS runners fast speed: 213 ± 12	-<br>-<br>-<br>-<br>-<br>-
Nelson et al. [49]	3.35 m/s: 255 ± 21.13[⁑]<br>4.88 m/s: 207 ± 12.0[⁑]<br>6.4 m/s: 175 ± 8.0[⁑]	3.35 m/s: 254 ± 22.00[⁑]<br>4.88 m/s: 201 ± 12.0[⁑]<br>6.4 m/s: 162 ± 9.0[⁑]	*p* = 0.81; ↔<br>*p* = 0.18; ↔<br>*p* = 0.002; ↑
Schache et al. [19] [a]	Left: 228.82 ± 14.54<br>Right: 226.01 ± 13.18	Left: 220.50 ± 11.33<br>Right: 223.88 ± 13.73	-<br>-
Sinclair et al. [67]	290 ± 30	230 ± 50	*p* = 0.005; ↑
Wank et al. [69]	4.0 m/s: 209 ± 18<br>6.0 m/s: 157 ± 8	4.0 m/s: 219 ± 17<br>6.0 m/s: 164 ± 9	*p* = 0.002; ↓<br>*p* = 0.002; ↓
Willy et al. [16]	273.1 ± 30.6	277.3 ± 26.1	*p* = 0.230; ↔
	**Relative contact time plantar region in relation to whole foot (%)**		
Hong et al. [27]	Medial head: 40.6 ± 5.6	Medial head/concrete: 38.6 ± 9.2<br>Medial head/natural grass: 39± 5.4	n.s.; ↔<br>n.s.; ↔
Hong et al. [27]	Lateral head: 40 ± 6.1	Lateral head/concrete: 36.8 ± 9.3	n.s.; ↔

Email corresponding author: basvanhooren@hotmail.com. Department of Nutrition and Movement Sciences, NUTRIM School of Nutrition and Translational Research in Metabolism, Maastricht University Medical Centre+, Maastricht, The Netherlands

		Lateral head/natural grass: 37.2 ± 5.9	n.s.; ↔
Hong et al. [27]	Medial midfoot: 36.9 ± 6.3	Medial midfoot/concrete: 34.6 ± 8.8<br>Medial midfoot/natural grass: 36.6 ± 8.4	n.s.; ↔<br>n.s.; ↔
Hong et al. [27]	Lateral midfoot: 52.50 ± 7.6	Lateral midfoot/concrete: 53.20 ± 14.4<br>Lateral midfoot/natural grass: 53.00 ± 9.7	n.s.; ↔<br>n.s.; ↔
Hong et al. [27]	Medial forefoot: 70.80 ± 5.2	Medial forefoot/concrete: 75.90 ± 11.8<br>Medial forefoot/natural grass: 79.50 ± 10.8	n.s.; ↔<br>n.s.; ↔
Hong et al. [27]	Central forefoot: 74.60 ± 6.4	Central forefoot/concrete: 79.20 ± 10.0<br>Central forefoot/natural grass: 81.90 ± 9.4	n.s.; ↔<br>n.s.; ↔
Hong et al. [27]	Lateral forefoot: 71.00 ± 9.4	Lateral forefoot/concrete: 74.70 ± 10.9<br>Lateral forefoot/natural grass: 74.60 ± 10.7	n.s.; ↔<br>n.s.; ↔
Hong et al. [27]	Greater toe: 67.70 ± 9.1	Greater toe/concrete: 68.00 ± 11.4<br>Greater toe/natural grass: 71.00 ± 10.5	n.s.; ↔<br>n.s.; ↔
Hong et al. [27]	Lesser toes: 68.10 ± 7.9	Lesser toes/concrete: 70.30 ± 12.8<br>Lesser toes/natural grass: 71.20 ± 11.8	n.s.; ↔<br>n.s.; ↔
		**Stride time (ms)**	
Baur et al. [51]	712 ± 29	718 ± 24	n.s.; ↔
Cronin, Finni [54]*	746 ± 40	763 ± 53	n.s.; ↔
Elliott, Blanksby [13][a]	Males at 3.70 m/s: 720 ± 34<br>Males at 5.41 m/s: 640 ± 30<br>Females at 3.97 m/s: 710 ± 24<br>Females at 5.29 m/s: 590 ± 24	Males at 3.70 m/s: 710 ± 26<br>Males at 5.41 m/s: 658 ± 26<br>Females at 3.97 m/s: 720 ± 32<br>Females at 5.29 m/s: 660 ± 22	$p = 0.14$; ↔<br>$p = 0.03$; ↓<br>$p = 0.14$; ↔<br>$p < 0.001$; ↓
Montgomery et al. [61]	2.88 m/s: 746 ± 47<br>4.28 m/s: 672 ± 55	2.88 m/s: 744 ± 40<br>4.28 m/s: 692 ± 39	$p = 0.98$; ↔<br>$p = 0.30$; ↔
Nelson et al. [49][a]	3.35 m/s: 748 ± 34[δ]<br>4.88 m/s: 684 ± 30[δ]<br>6.4 m/s: 636 ± 26[δ]	3.35 m/s: 746 ± 26[δ]<br>4.88 m/s: 682 ± 26[δ]<br>6.4 m/s: 604 ± 26[δ]	$p = 1$; ↔<br>$p = 0.38$; ↔<br>$p = 0.59$; ↔

Email corresponding author: basvanhooren@hotmail.com. Department of Nutrition and Movement Sciences, NUTRIM School of Nutrition and Translational Research in Metabolism, Maastricht University Medical Centre+, Maastricht, The Netherlands

Riley et al. [63]	690 ± 4	710 ± 6	p = 0.001; ↓
Schache et al. [19]	Left : 720 ± 4<br>Right: 720 ± 4	Left: 750 ± 3<br>Right: 750 ± 4	p = 0.02; ↓<br>p = 0.05; ↓
		**Duty factor (%)**	
Cronin, Finni [54]	41 ± 4	38 ± 3	n.s.; ↔
		**Toe off (% cycle)**	
Riley et al. [63]	33.26 ± 3.53	33.52 ± 2.91	p = 0.686; ↔
		**Swing duration (ms)**	
Cronin, Finni [54]	443 ± 17	470 ± 23	n.s.; ↔
		**Stride length (m)**	
Elliott, Blanksby [13][†,b]	Males at 3.70 m/s: 2.66 ± 0.28<br>Males at 5.41 m/s: 3.46 ± 0.16<br>Females at 3.97 m/s: 2.88 ± 0.42<br>Females at 5.29 m/s: 3.12 ± 0.24	Males at 3.70 m/s: 2.62 ± 0.28<br>Males at 5.41 m/s: 3.58 ± 0.28<br>Females at 3.97 m/s: 3.12 ± 0.38<br>Females at 5.29 m/s: 3.48 ± 0.28	p = 0.25; ↔<br>p = 0.001; ↓<br>p = 0.12; ↔<br>p < 0.001; ↓
Fullenkamp et al. [48] [b]	1.36 ± 0.16	1.36 ± 0.16	n.s.; ↔
Garcia-Perez et al. [57]	3.33 m/s: 2.28 ± 0.03<br>4.0 m/s: 2.58 ± 0.04	3.33 m/s: 2.25 ± 0.03<br>4.0 m/s: 2.51 ± 0.04	n.s.; ↔<br>n.s.; ↔
Nelson et al. [49][‡, b]	3.35 m/s: 2.62 ± 0.28[∅]<br>4.88 m/s: 3.44 ± 0.16 [∅]<br>6.4 m/s: 4.02 ± 0.18 [∅]	3.35 m/s: 2.60 ± 0.28 [∅]<br>4.88 m/s: 3.42 ± 0.14 [∅]<br>6.4 m/s: 3.84 ± 0.18 [∅]	p = 0.67; ↔<br>p = 0.62; ↔<br>p = 0.002; ↑
Riley et al. [63]	2.60 ± 0.36	2.71 ± 0.36	p = 0.001; ↓
Schache et al. [19]	Left: 2.85 ± 0.32<br>Right: 2.85 ± 0.32	Left: 2.99 ± 0.3<br>Right: 2.96 ± 0.29	p = 0.002; ↓<br>p = 0.001; ↓
Wank et al. [69] [b]	4.0 m/s: 2.92 ± 0.14<br>6.0 m/s: 3.64 ± 0.18	4.0 m/s: 3.04 ± 0.14<br>6.0 m/s: 3.88 ± 0.18	p < 0.001; ↓<br>p < 0.001; ↓
Willy et al. [16] [b]	1.04 ± 0.1	1.10 ± 0.12	p < 0.001; ↓

Email corresponding author: basvanhooren@hotmail.com. Department of Nutrition and Movement Sciences, NUTRIM School of Nutrition and Translational Research in Metabolism, Maastricht University Medical Centre+, Maastricht, The Netherlands

		**Step width (cm)**		
Fullenkamp et al. [48]		8.1 ± 2.2	8.1 ± 2.6	n.s.; ↔
Wank et al. [69]		No data reported/provided	No data reported/provided	-
		**Stride frequency (strides/s)**		
Brookes et al. [43][c]		6.3 mph: 2.86 ± 0.11<br>9.2 mph: 2.95 ± 0.13<br>11.5 mph: 3.14 ± 0.11<br>12.2 mph: 3.38 ± 0.13	6.3 mph: 2.86 ± 0.04<br>9.2 mph: 2.98 ± 0.09<br>11.5 mph: 3.08 ± 0.10<br>12.2 mph: 3.28 ± 0.13	n.s.; ↔<br>n.s.; ↔<br>n.s.; ↔<br>n.s.; ↔
Elliott, Blanksby [13]		Males at 3.70 m/s: 2.80 ± 0.13<br>Males at 5.41 m/s: 3.14 ± 0.21<br>Females at 3.97 m/s: 2.85 ± 0.29<br>Females at 5.29 m/s: 3.40 ± 0.23	Males at 3.70 m/s: 2.82 ± 0.11<br>Males at 5.41 m/s: 3.04 ± 0.17<br>Females at 3.97 m/s: 2.80 ± 0.25<br>Females at 5.29 m/s: 3.07 ± 0.19	$p = 0.23$; ↔<br>$p = 0.001$; ↑<br>$p = 0.19$; ↔<br>$p < 0.001$; ↑
Fullenkamp et al. [48][d]		1.33 ± 0.07	1.35 ± 0.07	n.s.; ↔
Garcia-Perez et al. [57][d]		3.33 m/s: 1.47 ± 0.02<br>4 m/s: 1.57 ± 0.02	3.33 m/s: 1.49 ± 0.02<br>4 m/s: 1.61 ± 0.03	n.s.; ↔<br>n.s.; ↔
Nelson et al. [49]		3.35 m/s: 2.69 ± 0.13[Δ]<br>4.88 m/s: 2.94 ± 0.21[Δ]<br>6.4 m/s: 3.15 ± 0.13[Δ]	3.35 m/s: 2.69 ± 0.11[Δ]<br>4.88 m/s: 2.96 ± 0.17[Δ]<br>6.4 m/s: 3.32 ± 0.13[Δ]	$p = 1$; ↔<br>$p = 0.78$; ↔<br>$p < 0.001$; ↓
Oliveira et al. [50][d]		1.38 ± 0.08	1.39 ± 0.09	n.s.; ↔
Riley et al. [63] [d]		1.46 ± 0.09	1.42 ± 0.13	$p = 0.009$; ↑
Wank et al. [69][d]		4.0 m/s: 1.38 ± 0.06<br>6.0 m/s: 1.64 ± 0.08	4.0 m/s: 1.33 ± 0.06<br>6.0 m/s: 1.55 ± 0.08	$p < 0.001$; ↑<br>$p < 0.001$; ↑
		**Non-linear measures**		
Lindsay et al. [59]	Detrended fluctuation analysis (α)[1]	80% preferred speed: 1.04 ± 0.18<br>100% preferred speed: 0.98 ± 0.17<br>120% preferred speed: 1.05 ± 0.19	80% preferred speed: 0.86 ± 0.17<br>100% preferred speed: 0.86 ± 0.14<br>120% preferred speed: 0.84 ± 0.17	-<br>-<br>-
Lindsay et al. [59]	Power spectral density analysis (β)[2]	80% preferred speed: 0.99 ± 0.44<br>100% preferred speed: 1.11 ± 0.27<br>120% preferred speed: 1.02 ± 0.33	80% preferred speed: 0.7 ± 0.36<br>100% preferred speed: 0.73 ± 0.31<br>120% preferred speed: 0.7 ± 0.39	-<br>-<br>-

Email corresponding author: basvanhooren@hotmail.com. Department of Nutrition and Movement Sciences, NUTRIM School of Nutrition and Translational Research in Metabolism, Maastricht University Medical Centre+, Maastricht, The Netherlands

HS, heel strike; NHS, non-heel strike; TM, treadmill.
[a] Converted from step time to stride time; [b] converted from step length to stride length; [c] Data obtained by averaging over all individual data reported and then converted from strides/min to strides/s; [d] converted to strides/s.
* This variable was reported as step duration. However, the time in ms, corresponds very well with stride time reported in other studies, including studies that used a similar speed. Further, the time in ms is longer than step time reported by other studies (see table) and therefore we have assumed that this variable reflects stride time rather than step time; [†] This variable was reported as stride length, but in the method section the authors describe that this was measured as the distance covered from toe-off until the next foot plant of the other foot and hence reflects a step and not a stride; [‡] This variable was reported as stride length, but in the method section the authors describe that this was measured as the distance between take-off of the right foot and ground contact of the left foot and hence reflects a step and not a stride. [⁑] SD at slowed speed was taken from slow running speed of heel strike running males in study from Kluitenberg et al. [40], SD at medium speed was also taken from fast running speed of non-heel strike male runners in Kluitenberg et al. [40] and SD at highest speed taken from highest speed in Wank et al. [69]; [δ] SD at slowest speed and medium speed taken from slowest and fastest speed in Elliott, Blanksby [13], while SD at fastest speed was estimated based on SD's reported by Elliot; [≈] SD at slowest and medium speed taken from slowest and fastest speed in Elliott, Blanksby [13], while SD at fastest speed was taken from fastest speed in Wank et al. [69]; [Δ], SD at slowest and medium speed taken from slowest and fastest speed in Elliott, Blanksby [13], while SD at fastest speed was taken from fastest speed in Brookes et al. [43].
[1] Indicates the regularity of stride dynamics as anti-correlated (α <0.5), white noise (α 0.5), correlated α >.05) and Brownian motion (α.1.5); [2] also provides information on the regularity of stride dynamics.

Email corresponding author: basvanhooren@hotmail.com. Department of Nutrition and Movement Sciences, NUTRIM School of Nutrition and Translational Research in Metabolism, Maastricht University Medical Centre+, Maastricht, The Netherlands

Table SII. Ankle kinematic outcome measures

Study	Outcome	Treadmill mean ± SD	Overground mean ± SD	P-value (when available); arrow indicating direction of effect compared to overground
		**Navicular kinematics**		
Barton et al. [52]	Navicular motion magnitude (mm)	9.9 ± 3.9	8.6 ± 2.5	0.003; ↑
Barton et al. [52]	Navicular velocity (mm/s)	394 ± 169	396 ± 140	0.85; ↔
		**Sagittal plane ankle angle at footstrike** (˚, positive values indicate dorsiflexion relative to neutral)		
Chambon et al. [41]	Rearfoot sagittal plane angle at footstrike (˚)	Shoe with 0 mm drop: 2.4 ± 8.3<br>Shoe with 4 mm drop: 4.0 ± 7.3<br>Shoe with 8 mm drop: 5.4 ± 5.4	Shoe with 0 mm drop: 8.5 ± 7.9<br>Shoe with 4 mm drop: 11.1 ± 8.2<br>Shoe with 8 mm drop: 10.5 ± 7.4	-<br>-<br>-
Fellin et al. [55]	Rearfoot sagittal plane angle at footstrike (˚)	4.5 ± 3.7	9.0 ± 3.8	-
Firminger et al. [56]	Ankle angle at heel strike (˚)	10 km/h: 10.49 ± 11.26<br>13 km/h: 10.87 ± 12.76	10 km/h: 14.65 ± 11.05<br>13 km/h: 16.09 ± 12.53	$p = 0.46$; ↔<br>$p = 0.42$; ↔
Pink et al. [62]	Ankle angle at footstrike (˚)	2.95 m/s: No data reported/provided<br>3.94 m/s: No data reported/provided	2.95 m/s: No data reported/provided<br>3.94 m/s: No data reported/provided	n.s.; ↔<br>n.s.; ↔
Sinclair et al. [66]	Ankle angle at footstrike (˚)	4.9 ± 4.0	0.6 ± 8.8	$p = 0.136$; ↔
		**Sagittal plane foot-ground angle with ground at footstrike** (˚, positive values indicate more inclination)		
Chambon et al. [41]	Horizontal foot angle and lab coordinate system (˚)	Shoe with 0 mm drop: 5.2 ± 8.6<br>Shoe with 4 mm drop: 8.0 ± 8.3<br>Shoe with 8 mm drop: 11.4 ± 5.6	Shoe with 0 mm drop: 16.1 ± 8.3<br>Shoe with 4 mm drop: 19.6 ± 8.9<br>Shoe with 8 mm drop: 20.3 ± 8.3	$p < 0.01$; ↓<br>$p < 0.01$; ↓<br>$p < 0.01$; ↓
Nigg et al. [14]	Horizontal foot angle and lab coordinate system (˚)	No data reported/provided for all speeds (3, 4.5, 5, 6 m/s) both shoe types	No data reported/provided for all speeds (3, 4.5, 5, 6 m/s) both shoe types	-

Email corresponding author: basvanhooren@hotmail.com. Department of Nutrition and Movement Sciences, NUTRIM School of Nutrition and Translational Research in Metabolism, Maastricht University Medical Centre+, Maastricht, The Netherlands

Wank et al. [69]	Horizontal foot angle and lab coordinate system (˚)	4.0 m/s: 6.7 ± 10.8<br>6.0 m/s: 1.3 ± 8.9	4.0 m/s: 17.6 ± 12.5<br>6.0 m/s: 6.9 ± 10.5	p = 0.006; ↓<br>p = 0.008; ↓
		**Peak ankle angle during stance** (˚, positive values indicate dorsiflexion relative to neutral)		
Fellin et al. [55]	Peak rearfoot angle sagittal plane (˚)	Peak during stance: 21.7 ± 4	Peak during stance: 22.9 ± 3.4	-
Fullenkamp et al. [48]	Rearfoot angle sagittal plane (˚)	24.0 ± 3.1	24.0 ± 2.7	n.s.; ↔
Sinclair et al. [66]	Rearfoot angle sagittal plane (˚)	16.0 ± 5.7	18.2 ± 3.8	p = 0.258; ↔
Willy et al. [16]	Rearfoot angle sagittal plane (˚)	22.4 ± 3	22.8 ± 3	p = 0.320; ↔
		**Ankle range of motion from footstrike to toe-off** (˚, positive values indicate dorsiflexion excursion)		
Fullenkamp et al. [48]	Rearfoot angle sagittal plane ROM during stance (˚)	44.6 ± 5.3	43.7 ± 4.2	n.s.; ↔
Pink et al. [62]	Rearfoot angle sagittal plane ROM during stance (˚)	2.95 m/s: No data reported/provided<br>3.94 m/s: No data reported/provided	2.95 m/s: No data reported/provided<br>3.94 m/s: No data reported/provided	n.s.; ↔<br>n.s.; ↔
		**Ankle dorsiflexion range of motion from footstrike to peak dorsiflexion** (˚, positive values indicate dorsiflexion excursion)		
Chambon et al. [41]	Ankle dorsiflexion ROM from footstrike to peak dorsiflexion angle during stance (˚)	Shoe with 0 mm drop: 26.8 ± 6.7<br>Shoe with 4 mm drop: 24.4 ± 5.8<br>Shoe with 8 mm drop: 22.4 ± 4.1	Shoe with 0 mm drop: 21.5 ± 6.5<br>Shoe with 4 mm drop: 18.4 ± 6.5<br>Shoe with 8 mm drop: 17.9 ± 6.1	-<br>-<br>-
Sinclair et al. [66]	Ankle dorsiflexion ROM from footstrike to peak dorsiflexion angle during stance (˚)	11.1 ± 4.3	17.6 ± 7.9	p = 0.015; ↓
		**Ankle angle at mid-stance** (˚)		

Email corresponding author: basvanhooren@hotmail.com. Department of Nutrition and Movement Sciences, NUTRIM School of Nutrition and Translational Research in Metabolism, Maastricht University Medical Centre+, Maastricht, The Netherlands

Firminger et al. [56]	Ankle angle at mid-stance (50% stance phase, ˚)	10 km/h: 21.68 ± 5.68<br>13 km/h: 22.73 ± 5.97	10 km/h: 23.95 ± 5.57<br>13 km/h: 23.96 ± 5.86	*p* = 0.43; ↔<br>*p* = 0.68; ↔
Pink et al. [62]	Ankle sagittal plane angle midstance (˚)	2.95 m/s: No data reported/provided<br>3.94 m/s: No data reported/provided	2.95 m/s: No data reported/provided<br>3.94 m/s: No data reported/provided	n.s.; ↔<br>n.s.; ↔
			**Ankle angle at toe-off** (˚)	
Firminger et al. [56]	Ankle angle at toe-off (˚)	10 km/h: -12.68 ± 8.15<br>13 km/h: -15.82 ± 7.93	10 km/h: -12.67 ± 8.00<br>13 km/h: -12.83 ± 7.78	*p* = 1.00; ↔<br>*p* = 0.46; ↔
Pink et al. [62]	Ankle sagittal plane angle before toe-off (˚)	2.95 m/s: No data reported/provided<br>3.94 m/s: No data reported/provided	2.95 m/s: No data reported/provided<br>3.94 m/s: No data reported/provided	n.s.; ↔<br>n.s.; ↔
			**Other ankle kinematics sagittal plane** (positive values indicate dorsiflexion)	
Fullenkamp et al. [48]	Peak plantarflexion angle (˚)	20.5 ± 4.3	19.7 ± 3.8	n.s.; ↔
Pink et al. [62]	Ankle sagittal plane angle during loading phase (˚)	2.95 m/s: No data reported/provided<br>3.94 m/s: No data reported/provided	2.95 m/s: No data reported/provided<br>3.94 m/s: No data reported/provided	n.s.; ↔<br>n.s.; ↔
Sinclair et al. [66]	Ankle ROM in sagittal plane during stance (˚)	15.1 ± 6.3	17.6 ± 10.5	*p* = 0.833; ↔
Sinclair et al. [66]	Peak dorsiflexion velocity in sagittal plane during loading phase(˚/s)	384.5 ± 145.2	575.5 ± 129	*p* = 0.001; ↓
Sinclair et al. [66]	Peak plantarflexion velocity during propulsive phase (˚/s)	-185.5 ± 66.6	-286.6 ± 50.2	*p* < 0.001; ↑
Wank et al. [69]	Angle between center of gravity, heel and vertical line at first ground contact (˚)*	4.0 m/s: 18.64 ± 2.95<br>6.0 m/s: 20.57 ± 2.55	4.0 m/s: 20.68 ± 2.43<br>6.0 m/s: 20.57 ± 2.55	-<br>-

Email corresponding author: basvanhooren@hotmail.com. Department of Nutrition and Movement Sciences, NUTRIM School of Nutrition and Translational Research in Metabolism, Maastricht University Medical Centre+, Maastricht, The Netherlands

Wank et al. [69]	Angle between center of gravity, ball and vertical line at first ground contact (˚)*	4.0 m/s: 25.90 ± 1.41<br>6.0 m/s: 30.72 ± 1.67	4.0 m/s: 25.16 ± 0.74<br>6.0 m/s: 30.18 ± 1.34	-<br>-
	**Frontal plane**			
		**Ankle in- and eversion at footstrike** (˚, positive angle represents inversion)		
Fellin et al. [55]	Rearfoot angle frontal plane (˚)	-3 ± 3.9	-1.8 ± 4	-
Nigg et al. [14]	Achilles tendon angle (β, angle between leg and heel on medial side, ˚)	No data reported/provided	No data reported/provided	No data reported/provided
Sinclair et al. [66]	Rearfoot angle frontal plane (˚)	-4.5 ± 10.1	2.0 ± 5.2	p = 0.013; ↔
		**Peak ankle in- and eversion during stance** (˚, positive angle represents inversion)		
Fellin et al. [55]	Rearfoot angle frontal plane (˚)	-12.5 ± 3.5	-12.9 ± 2.9	-
Sinclair et al. [66]	Peak ankle eversion (˚)	Peak: -15.5 ± 8.9	Peak: -9.2 ± 7.8	p = 0.006; ↓
		**Other ankle kinematics frontal plane** (positive values correspond with inversion)		
Nigg et al. [14]	Leg frontal plane angle (α, angle between leg and ground on medial side, (˚)	No data reported/provided	No data reported/provided	No data reported/provided
Nigg et al. [14]	Rear foot frontal plane angle (γ, angle between heel of shoe and ground on medial side, (˚)	No data reported/provided	No data reported/provided	No data reported/provided

Email corresponding author: basvanhooren@hotmail.com. Department of Nutrition and Movement Sciences, NUTRIM School of Nutrition and Translational Research in Metabolism, Maastricht University Medical Centre+, Maastricht, The Netherlands

Riley et al. [63]	Peak ankle eversion (˚), unclear during which phase of gait cycle	-1.9 ± 2.9	-2.5 ± 3.0	*p* = 0.006; ↓
Sinclair et al. [66]	ROM in frontal plane (˚)	4.0 ± 8.0	3.2 ± 5.0	*p* = 0.688; ↔
Sinclair et al. [66]	Excursion from footstrike to peak angle in frontal plane (˚)	11.0 ± 6.4	11.2 ± 4.6	*p* = 0.952; ↔
Sinclair et al. [66]	Peak inversion velocity (˚/s)	196.6 ± 120.6	161.4 ± 480	*p* = 0.325; ↔
Sinclair et al. [66]	Peak eversion velocity (˚/s)	-177.4 ± 144.4	-215.2 ± 61.8	*p* = 0.398; ↔
	**Transverse plane**			
		**Ankle add- and abduction at footstrike** (˚, negative values denote ankle abduction)		
Fellin et al. [55]	Rearfoot angle transverse plane (˚)	-11.4 ± 4.3	-9.7 ± 4.9	-
Sinclair et al. [66]	Rearfoot angle transverse plane (˚)	-9.6 ± 4.9	-13.5 ± 5.1	*p* = 0.007; ↑
		**Peak ankle add- and abduction during stance** (˚, negative values denote ankle abduction)		
Fellin et al. [55]	Rearfoot angle transverse plane (˚)	-16.5 ± 4.7	-15.9 ± 4	-
Sinclair et al. [66]	Rearfoot angle transverse plane (˚)	-1.1 ± 3	-2.7 ± 4.3	*p* = 0.239; ↔
		**Other ankle kinematics transverse plane** (negative values denote ankle abduction)		
Sinclair et al. [66]	Excursion in transverse plane from footstrike to toe off (˚)	0.7 ± 11.2	3.2 ± 3.5	*p* = 0.457; ↔
Sinclair et al. [66]	Excursion from footstrike to peak	8.5 ± 4.1	10.9 ± 3.5	*p* = 0.086; ↔

Email corresponding author: basvanhooren@hotmail.com. Department of Nutrition and Movement Sciences, NUTRIM School of Nutrition and Translational Research in Metabolism, Maastricht University Medical Centre+, Maastricht, The Netherlands

	angle in transverse plane (˚)			
Sinclair et al. [66]	Peak external rotation rotation velocity in transverse plane (˚/s)	210.6 ± 146.9	209.7 ± 89.4	$p = 0.985$; ↔
Sinclair et al. [66]	Peak internal rotation velocity in transverse plane (˚/s)	-136.6 ± 48.8	-155.4 ± 89.8	$p = 0.517$; ↔
		**Rearfoot-tibial kinematics** (˚, positive angle is dorsiflexion) ‡		
	**Sagittal plane**			
Sinclair et al. [67]	Angle at footstrike (˚)	-3.34 ± 10.61	2.3 ± 4.57	$p < 0.05$; ↓
Sinclair et al. [67]	Angle at toe-off (˚)	-16.92 ± 7.91	-18.02 ± 4.81	n.s.; ↔
Sinclair et al. [67]	Peak angle (˚)	13.46 ± 5.64	16.18 ± 3.58	n.s.; ↔
Sinclair et al. [67]	ROM (˚)	13.58 ± 5.28	20.32 ± 4.92	$p < 0.05$; ↓
Sinclair et al. [67]	Relative ROM (˚)	16.8 ± 10.5	13.88 ± 4.68	n.s.; ↔
	**Coronal plane**			
Sinclair et al. [67]	Angle at footstrike (˚)	2.93 ± 4.33	2.82 ± 4.67	n.s.; ↔
Sinclair et al. [67]	Angle at toe-off (˚)	2.82 ± 5.08	3.31 ± 4.35	n.s.; ↔
Sinclair et al. [67]	Peak angle (˚)	-7.41 ± 3.94	-6.95 ± 4.37	n.s.; ↔
Sinclair et al. [67]	ROM (˚)	3.87 ± 2.15	3.88 ± 2.78	n.s.; ↔
Sinclair et al. [67]	Relative ROM (˚)	10.34 ± 3.92	9.77 ± 3.94	n.s.; ↔
	**Transverse plane**			
Sinclair et al. [67]	Angle at footstrike (˚)	-1.15 ± 2.19	-1.62 ± 2.59	n.s.; ↔
Sinclair et al. [67]	Angle at toe-off (˚)	2.11 ± 3.55	1.55 ± 2.21	n.s.; ↔
Sinclair et al. [67]	Peak angle (˚)	-6.79 ± 3.28	-7.02 ± 2.84	n.s.; ↔

Email corresponding author: basvanhooren@hotmail.com. Department of Nutrition and Movement Sciences, NUTRIM School of Nutrition and Translational Research in Metabolism, Maastricht University Medical Centre+, Maastricht, The Netherlands

Sinclair et al. [67]	ROM (˚)	3.85 ± 2.75	3.49 ± 1.86	n.s.; ↔
Sinclair et al. [67]	Relative ROM (˚)	5.64 ± 3.03	5.4 ± 2.56	n.s.; ↔
		**Midfoot-rearfoot kinematics**‡		
	**Sagittal plane**			
Sinclair et al. [67]	Angle at footstrike (˚)	1.8 ± 2.78	1.8 ± 1.44	n.s.; ↔
Sinclair et al. [67]	Angle at toe-off (˚)	-1.11 ± 3.71	-3.26 ± 2.47	n.s.; ↔
Sinclair et al. [67]	Peak angle (˚)	6.24 ± 2.1	5.82 ± 2.18	n.s.; ↔
Sinclair et al. [67]	ROM (˚)	4.49 ± 2.56	5.06 ± 1.64	n.s.; ↔
Sinclair et al. [67]	Relative ROM (˚)	4.43 ± 3	4.02 ± 1.61	n.s.; ↔
	**Coronal plane**			
Sinclair et al. [67]	Angle at footstrike (˚)	-1.61 ± 2.08	0.14 ± 1.5	n.s.; ↔
Sinclair et al. [67]	Angle at toe-off (˚)	-2.19 ± 3.42	-1 ± 1.34	n.s.; ↔
Sinclair et al. [67]	Peak angle (˚)	-0.08 ± 2.56	0.72 ± 1.4	n.s.; ↔
Sinclair et al. [67]	ROM (˚)	2.43 ± 1.88	1.47 ± 0.88	n.s.; ↔
Sinclair et al. [67]	Relative ROM (˚)	1.53 ± 2.51	0.58 ± 0.69	n.s.; ↔
	**Transverse plane**			
Sinclair et al. [67]	Angle at footstrike (˚)	1.46 ± 1.15	0.81 ± 0.8	n.s.; ↔
Sinclair et al. [67]	Angle at toe-off (˚)	2.23 ± 1.69	1.77 ± 0.87	n.s.; ↔
Sinclair et al. [67]	Peak angle (˚)	-0.65 ± 1.27	-0.85 ± 1.04	n.s.; ↔
Sinclair et al. [67]	ROM (˚)	1.3 ± 1.25	1.24 ± 0.8	n.s.; ↔
Sinclair et al. [67]	Relative ROM (˚)	2.11 ± 1.35	1.67 ± 1.04	n.s.; ↔
		**Forefoot-midfoot kinematics**‡		

Email corresponding author: basvanhooren@hotmail.com. Department of Nutrition and Movement Sciences, NUTRIM School of Nutrition and Translational Research in Metabolism, Maastricht University Medical Centre+, Maastricht, The Netherlands

	**Sagittal plane**			
Sinclair et al. [67]	Angle at footstrike (˚)	4.48 ± 6.17	4.06 ± 2.89	n.s.; ↔
Sinclair et al. [67]	Angle at toe-off (˚)	13.51 ± 11.77	14.46 ± 4.41	n.s.; ↔
Sinclair et al. [67]	Peak angle (˚)	19.32 ± 9.44	19.37 ± 4.6	n.s.; ↔
Sinclair et al. [67]	ROM (˚)	11.5 ± 4.36	10.4 ± 3.47	n.s.; ↔
Sinclair et al. [67]	Relative ROM (˚)	14.83 ± 4.15	15.31 ± 3.7	n.s.; ↔
	**Coronal plane**			
Sinclair et al. [67]	Angle at footstrike (˚)	0.11 ± 1.28	-0.6 ± 0.6	n.s.; ↔
Sinclair et al. [67]	Angle at toe-off (˚)	1.61 ± 2.12	0.89 ± 0.85	n.s.; ↔
Sinclair et al. [67]	Peak angle (˚)	2.27 ± 2.14	1.21 ± 1.09	n.s.; ↔
Sinclair et al. [67]	ROM (˚)	1.83 ± 1.26	1.48 ± 0.73	n.s.; ↔
Sinclair et al. [67]	Relative ROM (˚)	2.16 ± 1.54	1.81 ± 0.97	n.s.; ↔
	**Transverse plane**			
Sinclair et al. [67]	Angle at footstrike (˚)	0.14 ± 1.73	-0.37 ± 1.06	n.s.; ↔
Sinclair et al. [67]	Angle at toe-off (˚)	1.38 ± 1.16	0.57 ± 1.55	n.s.; ↔
Sinclair et al. [67]	Peak angle (˚)	2.81 ± 1.48	1.56 ± 1.66	n.s.; ↔
Sinclair et al. [67]	ROM (˚)	2.53 ± 1.68	1.42 ± 0.76	n.s.; ↔
Sinclair et al. [67]	Relative ROM (˚)	2.67 ± 1.24	1.93 ± 1.25	n.s.; ↔
			**Forefoot-rearfoot kinematics**[‡]	
	**Sagittal plane**			
Sinclair et al. [67]	Angle at footstrike (˚)	6.17 ± 5.87	5.65 ± 3	n.s.; ↔
Sinclair et al. [67]	Angle at toe-off (˚)	12.41 ± 9.34	11.04 ± 4.39	n.s.; ↔
Sinclair et al. [67]	Peak angle (˚)	18.42 ± 8.44	18.21 ± 3.92	n.s.; ↔

Email corresponding author: basvanhooren@hotmail.com. Department of Nutrition and Movement Sciences, NUTRIM School of Nutrition and Translational Research in Metabolism, Maastricht University Medical Centre+, Maastricht, The Netherlands

Sinclair et al. [67]	ROM (˚)	7.57 ± 3.97	5.79 ± 2.76	n.s.; ↔
Sinclair et al. [67]	Relative ROM (˚)	12.25 ± 5.01	12.56 ± 3.92	n.s.; ↔
	**Coronal plane**			
Sinclair et al. [67]	Angle at footstrike (˚)	-1.11 ± 3.33	0.85 ± 2.31	n.s.; ↔
Sinclair et al. [67]	Angle at toe-off (˚)	-1.56 ± 4.54	0.29 ± 2.64	n.s.; ↔
Sinclair et al. [67]	Peak angle (˚)	1.37 ± 3.15	1.89 ± 2.41	n.s.; ↔
Sinclair et al. [67]	ROM (˚)	3.97 ± 3.55	1.58 ± 0.99	n.s.; ↔
Sinclair et al. [67]	Relative ROM (˚)	2.48 ± 3.19	1.04 ± 1.18	n.s.; ↔
	**Transverse plane**			
Sinclair et al. [67]	Angle at footstrike (˚)	1.23 ± 2.1	-0.11 ± 0.91	n.s.; ↔
Sinclair et al. [67]	Angle at toe-off (˚)	2.41 ± 2.73	1.35 ± 0.93	n.s.; ↔
Sinclair et al. [67]	Peak angle (˚)	-1.05 ± 1.44	-1.6 ± 0.83	n.s.; ↔
Sinclair et al. [67]	ROM (˚)	2.36 ± 1.67	1.84 ± 0.75	n.s.; ↔
Sinclair et al. [67]	Relative ROM (˚)	2.28 ± 2.11	1.49 ± 1.29	n.s.; ↔
		**Trend symmetry values (unitless)[a]**		
Fellin et al. [55]	Rearfoot sagittal	0.97 ± 0.02		n.a.
Fellin et al. [55]	Rearfoot frontal	0.98 ± 0.02		n.a.
Fellin et al. [55]	Rearfoot transverse	0.89 ± 0.12		n.a.
		**Range offset (˚)[b]**		
Fellin et al. [55]	Rearfoot sagittal	0.7 ± 2.7		n.a.
Fellin et al. [55]	Rearfoot frontal	-0.3 ± 2.0		n.a.
Fellin et al. [55]	Rearfoot transverse	1 ± 2.0		n.a.

Email corresponding author: basvanhooren@hotmail.com. Department of Nutrition and Movement Sciences, NUTRIM School of Nutrition and Translational Research in Metabolism, Maastricht University Medical Centre+, Maastricht, The Netherlands

		**Range amplitude (unitless)[c]**	
Fellin et al. [55]	Rearfoot sagittal	0.92 ± 0.12	n.a.
Fellin et al. [55]	Rearfoot frontal	1.04 ± 0.14	n.a.
Fellin et al. [55]	Rearfoot transverse	0.99 ± 0.15	n.a.
		**Phase offset (%)[d]**	
Fellin et al. [55]	Rearfoot sagittal	0 ± 0	n.a.
Fellin et al. [55]	Rearfoot frontal	n/a	n.a.
Fellin et al. [55]	Rearfoot transverse	n/a	n.a.

[a] indicates how similar the trends are with values closer to 1 indicating higher similarities; [b] indicates the offset in the trend (positive = overground larger than treadmill; [c] > 1.0 indicates that overground excursions are larger than treadmill; ; [d] indicates the offset, with positive values indicating that overground shifted forward relative to stance time with respect to the treadmill curve.
*Data extracted using WebPlotDigitizer; ‡ The model used in this study was a multi-segmented foot model and hence not considered to be comparable to data from studies that used a rigid-segment foot model.

Email corresponding author: basvanhooren@hotmail.com. Department of Nutrition and Movement Sciences, NUTRIM School of Nutrition and Translational Research in Metabolism, Maastricht University Medical Centre+, Maastricht, The Netherlands

Table SIII. Knee kinematic outcome measures

Study	Outcome	Treadmill mean ± SD	Overground mean ± SD	P-value (when available); arrow indicating direction of effect compared to overground
		**Knee flexion angle at footstrike** (˚ , negative angle denotes knee flexion)		
	**Sagittal plane**			
Chambon et al. [41]	Knee flexion angle at footstrike (˚)	Shoe with 0 mm drop: -17.4 ± 4.9<br>Shoe with 4 mm drop: -16.6 ± 5.1<br>Shoe with 8 mm drop: -15.3 ± 4.2	Shoe with 0 mm drop: -14.3 ± 5.6<br>Shoe with 4 mm drop: -13.4 ± 4.9<br>Shoe with 8 mm drop: -13.3 ± 5.3	-<br>-<br>-
Fellin et al. [55]	Knee flexion angle at footstrike (˚)	-16 ± 6.7	-14.6 ± 4.9	-
Firminger et al. [56]	Knee flexion angle at heel strike (˚)	10 km/h: -18.09 ± 10.10<br>13 km/h: -18.10 ± 9.42	10 km/h: -12.43 ± 9.91<br>13 km/h: -15.08 ± 9.25	$p = 0.27$; ↔<br>$p = 0.52$; ↔
Nigg et al. [14]	Knee flexion angle (˚)	No data reported/provided	No data reported/provided	No data reported/provided
Sinclair et al. [66]	Knee flexion angle at footstrike (˚)	-19.1 ± 6.3	-18 ± 7	$p = 0.661$; ↔
Wank et al. [69]	Knee flexion angle at footstrike (˚)†	4.0 m/s: -9.7 ± 3.1<br>6.0 m/s: -12.3 ± 3.6	4.0 m/s: -7.3 ± 4<br>6.0 m/s: -9.3 ± 3.4	$p = 0.302$; ↔<br>$p = 0.026$; ↓
		**Knee flexion-extension range of motion sagittal plane** (˚ , negative angle denotes knee flexion)		
Fullenkamp et al. [48]	Knee flexion-extension ROM during full gait cycle (˚)	78.3 ± 9.7	80.1 ± 11.2	n.s.; ↔
Pink et al. [62]	Knee flexion-extension ROM (˚)	2.95 m/s: No data reported/provided<br>3.94 m/s: No data reported/provided	2.95 m/s: No data reported/provided<br>3.94 m/s: No data reported/provided	n.s.; ↔<br>n.s.; ↔
Sinclair et al. [66]	Knee flexion excursion from footstrike to toe-off (˚)	2.1 ± 8.1	1.1 ± 8.7	$p = 0.222$; ↔
		**Peak knee flexion angle sagittal plane during swing** (˚ , negative value denotes knee flexion)		

Email corresponding author: basvanhooren@hotmail.com. Department of Nutrition and Movement Sciences, NUTRIM School of Nutrition and Translational Research in Metabolism, Maastricht University Medical Centre+, Maastricht, The Netherlands

Fullenkamp et al. [48]	Max knee angle during swing (˚)	-84.1 ± 9.2	-86.1 ± 9.9	n.s.; ↔
Riley et al. [63]	Knee flexion during swing (˚)	-103.5 ± 12.2	-110.1 ± 18.4	*p* = 0.001; ↓
Wank et al. [69]	Min knee angle during entire gait cycle (˚)	4.0 m/s: -121.5 ± 9.52<br>6.0 m/s: -135 ± 6.4	4.0 m/s: -118.6 ± 8.74<br>6.0 m/s: -133.2 ± 7.0	*p* = 0.041; ↓<br>*p* = 0.120; ↔
		**Peak knee flexion angle sagittal plane during stance** (˚ , negative value denotes knee flexion)		
Fellin et al. [55]	Peak knee flexion angle during stance (˚)	-41.9 ± 4.4	-43.2 ± 3.4	-
Sinclair et al. [66]	Peak knee flexion during stance (˚)	-34.5 ± 5.7	-39.5 ± 5.2	*p* = 0.015; ↓
Wank et al. [69]	Peak knee flexion during stance (˚)	4.0 m/s: -43.3 ± 2<br>6.0 m/s: -41.0 ± 5.4	4.0 m/s: -41.8 ± 2.6<br>6.0 m/s: -38.3 ± 3.9	*p* = 0.007; ↓<br>*p* = 0.026; ↓
Willy et al. [16]	Peak knee flexion angle during stance (˚)	-34.2 ± 3.5	-34.3 ± 3.8	*p* = 0.960; ↔
		**Minimum knee flexion angle sagittal plane** (˚ , negative value denotes knee flexion)		
Fullenkamp et al. [48]	Min knee angle during entire gait cycle (˚)	-5.9 ± 3.7	-6.0 ± 5.6	n.s.; ↔
Riley et al. [63]	Min knee angle during entire gait cycle (˚)	-10.2 ± 5.4	-8.3 ± 6.0	*p* = 0.001; ↑
		**Knee flexion range of motion from footstrike to peak during stance** (˚ , negative value denotes knee flexion)		
Chambon et al. [41]	Knee flexion excursion from footstrike to peak angle during stance (˚)	Shoe with 0 mm drop: -27.9 ± 3.3<br>Shoe with 4 mm drop: -28.8 ± 3.8<br>Shoe with 8 mm drop: -30.9 ± 3.0	Shoe with 0 mm drop: -34.8 ± 4.6<br>Shoe with 4 mm drop: -35.5 ± 5.1<br>Shoe with 8 mm drop: -35.9 ± 5.5	-<br>-<br>-
Sinclair et al. [66]	Knee flexion excursion from footstrike to peak	-15.4 ± 6.2	-21.5 ± 7.5	*p* = 0.063; ↔

Email corresponding author: basvanhooren@hotmail.com. Department of Nutrition and Movement Sciences, NUTRIM School of Nutrition and Translational Research in Metabolism, Maastricht University Medical Centre+, Maastricht, The Netherlands

	angle in during stance (˚)			
		**Knee flexion angle at toe-off** (˚, negative values correspond with flexion)		
Firminger et al. [56]	Knee angle at toe-off (˚)	10 km/h: -17.33 ± 9.60<br>13 km/h: -14.86 ± 9.54	10 km/h: -15.62 ± 9.43<br>13 km/h: -15.19 ± 9.36	*p* = 0.72; ↔<br>*p* = 0.95; ↔
Wank et al. [69]	Knee flexion angle at toe-off (˚)	4.0 m/s: -16.0 ± 3.3<br>6.0 m/s: -17.4 ± 2.3	4.0 m/s: -15.5 ± 4.1<br>6.0 m/s: -16.6 ± 3	*p* = 0.596; ↔<br>*p* = 0.236; ↔
		**Other knee kinematics sagittal plane** (˚, negative values correspond with flexion)		
Firminger et al. [56]	Knee angle at mid-stance (˚)	10 km/h: -38.92 ± 9.39<br>13 km/h: -42.49 ± 9.21	10 km/h: -42.49 ± 9.21<br>13 km/h: -43.86 ± 8.59	*p* = 0.45; ↔<br>*p* = 0.53; ↔
Sinclair et al. [66]	Peak knee flexion angular velocity (˚/s)	-276.3 ± 96	-318.3 ± 87	*p* = 0.375; ↔
Sinclair et al. [66]	Peak knee extension angular velocity (˚/s)	+241.8 ± 116.3	+273.9 ± 72.9	*p* = 0.430; ↔
	**Frontal plane**			
		**Knee add- and abduction angle at footstrike** (˚, positive angle is knee adduction/varus)		
Fellin et al. [55]	Knee angle frontal plane (˚)	-1.3 ± 3.3	-1 ± 2.8	-
Sinclair et al. [66]	Knee angle frontal plane (˚)	1.7 ± 5.6	-0.4 ± 3.6	*p* = 0.119; ↔
		**Peak knee add- and abduction angle during stance** (˚, positive angle is knee adduction/varus)		
Fellin et al. [55]	Knee angle frontal plane (˚)	1.2 ± 4.5	2.2 ± 4.4	-
Sinclair et al. [66]	Knee angle frontal plane (˚)	-2.7 ± 9	-5.4 ± 5.4	*p* = 0.161; ↔
		**Other knee kinematics frontal plane** (positive angle is knee adduction/varus)		
Sinclair et al. [66]	Knee adduction ROM (˚) from footstrike to toe-off	3.5 ± 4.6	2.1 ± 8.1	*p* = 0.199; ↔

Email corresponding author: basvanhooren@hotmail.com. Department of Nutrition and Movement Sciences, NUTRIM School of Nutrition and Translational Research in Metabolism, Maastricht University Medical Centre+, Maastricht, The Netherlands

Sinclair et al. [66]	Knee adduction excursion from footstrike to peak angle in coronal plane (˚)	4.6 ± 4.2	5 ± 2.7	*p* = 0.601; ↔
Sinclair et al. [66]	Peak knee adduction velocity in coronal plane (˚/s)	72.7 ± 44.6	77.4 ± 31.6	*p* = 0.562; ↔
Sinclair et al. [66]	Peak knee abduction velocity in coronal plane (˚/s)	-87.4 ± 53.6	-73 ± 34.5	*p* = 0.324; ↔
	**Transverse plane**			
		**Knee in- and eternal rotation angle at footstrike** (˚, positive angle is knee internal rotation)		
Fellin et al. [55]	Knee angle transverse plane (˚)	-3.4 ± 7.7	-4.4 ± 7.4	-
Sinclair et al. [66]	Knee angle transverse plane (˚)	-3.6 ± 7.8	-4.5 ± 7.2	*p* = 0.257; ↔
		**Peak knee in- and eternal rotation angle during stance** (˚, positive angle is knee internal rotation)		
Fellin et al. [55]	Knee angle transverse plane (˚)	4.0 ± 5.9	5.2 ± 5.9	-
Sinclair et al. [66]	Knee angle transverse plane (˚)	6.4 ± 8	6.6 ± 6	*p* = 0.917; ↔
		**Other knee kinematics transverse plane** (positive angle is knee internal rotation)		
Sinclair et al. [66]	Knee internal rotation ROM (˚) from footstrike to toe-off	3.2 ± 9.9	3.2 ± 4	*p* = 0.991; ↔
Sinclair et al. [66]	Knee internal rotation excursion from footstrike to peak angle during stance (˚)	10 ± 7	11.2 ± 5	*p* = 0.616; ↔

Email corresponding author: basvanhooren@hotmail.com. Department of Nutrition and Movement Sciences, NUTRIM School of Nutrition and Translational Research in Metabolism, Maastricht University Medical Centre+, Maastricht, The Netherlands

Sinclair et al. [66]	Peak knee internal rotation velocity during stance (˚/s)	145.3 ± 58.8	166.6 ± 61.4	*p* = 0.326; ↔
Sinclair et al. [66]	Peak external rotation velocity during stance (˚/s)	-108.8 ± 89.5	-160.9 ± 80.4	*p* = 0.118; ↔
		**Trend symmetry values (unitless)[a]**		
Fellin et al. [55]	Knee sagittal	0.99 ± 0.00		n.a.
Fellin et al. [55]	Knee frontal	0.86 ± 0.12		n.a.
Fellin et al. [55]	Knee transverse	0.89 ± 0.14		n.a.
		**Range offset (˚degrees)[b]**		
Fellin et al. [55]	Knee sagittal	-0.7 ± 2.9		n.a.
Fellin et al. [55]	Knee frontal	0.6 ± 2.0		n.a.
Fellin et al. [55]	Knee transverse	0.1 ± 2.6		n.a.
		**Range amplitude (unitless)[c]**		
Fellin et al. [55]	Knee sagittal	0.96 ± 0.09		n.a.
Fellin et al. [55]	Knee frontal	0.96 ± 0.20		n.a.
Fellin et al. [55]	Knee transverse	0.82 ± 0.23		n.a.
		**Phase offset (%)[d]**		
Fellin et al. [55]	Knee sagittal	0.2 ± 2		n.a.
Fellin et al. [55]	Knee frontal	n/a		n.a.
Fellin et al. [55]	Knee transverse	n/a		n.a.

[a] indicates how similar the trends are with values closer to 1 indicating higher similarities; [b] indicates the offset in the trend (positive = overground larger than treadmill; [c] > 1.0 indicates that overground excursions are larger than treadmill; ; [d] indicates the offset, with positive values indicating that overground shifted forward relative to stance time with respect to the treadmill curve

[†] Converted to similar values as other studies by subtracting from 180˚.

Email corresponding author: basvanhooren@hotmail.com. Department of Nutrition and Movement Sciences, NUTRIM School of Nutrition and Translational Research in Metabolism, Maastricht University Medical Centre+, Maastricht, The Netherlands

Table SIV. Hip and pelvic kinematic outcome measures

Study	Outcome	Treadmill mean ± SD	Overground mean ± SD	P-value (when available); arrow indicating direction of effect compared to overground
		**Hip flexion angle at foot strike** (˚, positive values correspond with hip flexion)		
Fellin et al. [55]	Hip flexion angle at foot strike (˚)	28 ± 6.2	27.2 ± 5.7	-
Firminger et al. [56]	Hip flexion at heel strike (˚)	10 km/h: 24.59 ± 6.81<br>13 km/h: 14.48 ± 5.49	10 km/h: 25.28 ± 6.68<br>13 km/h: 15.56 ± 5.38	p = 0.84; ↔<br>p = 0.70; ↔
Schache et al. [19]	Hip flexion at foot strike (˚)	Left: 34.0 ± 5.2<br>Right: 35.4 ± 4.5	Left: 39.2 ± 3.8<br>Right: 38.3 ± 3.2	p = 0.001; ↓<br>p = 0.007; ↓
Sinclair et al. [66]	Hip flexion at foot strike (˚)	35.1 ± 12.7	47.1 ± 13.5	p < 0.001; ↓
		**Peak hip flexion angle during stance** (˚, positive values correspond with hip flexion)		
Fellin et al. [55]	Peak hip angle during stance (˚)	30.1 ± 4.9	30.8 ± 4.1	-
Schache et al. [19]	Peak hip flexion during stance (˚)	Left: 36.1 ± 4.3<br>Right: 37.0 ± 3.5	Left: 41.7 ± 4.0<br>Right: 39.7 ± 3.8‡	p = 0.004; ↓<br>p = 0.028; ↓
Sinclair et al. [66]	Peak hip flexion during stance (˚)	36.6 ± 7.9	49.3 ± 8.6	p < 0.001; ↓
		**Peak hip flexion angle during gait cycle** (˚, positive values correspond with hip flexion)		
Fullenkamp et al. [48]	Peak hip angle throughout gait cycle (˚)	34.9 ± 6.8	36.2 ± 6.8	n.s.; ↔
Schache et al. [19]	Peak hip flexion throughout gait cycle (˚)	Left: 49.9 ± 5.1<br>Right: 52 ± 5.1	Left: 51.0 ± 6.2<br>Right: 51.3 ± 6.0	p = 0.477; ↔<br>p = 0.562; ↔

Wank et al. [69]	Peak hip flexion throughout gait cycle (˚)*†	4.0 m/s: 39.26 ± 6.29<br>6.0 m/s: 51.18 ± 5.79	4.0 m/s: 43.74 ± 5.28<br>6.0 m/s: 57.90 ± 7.55	-<br>-
		**Hip range of motion sagittal plane during stance** (˚, positive values correspond with hip flexion)		
Fullenkamp et al. [48]	Hip flexion-extension excursion during stance (˚)	47.7 ± 7.4	49.2 ± 6.8	n.s.; ↔
Sinclair et al. [66]	Hip flexion-extension excursion during stance (˚)	32.6 ± 10.5	49.6 ± 10.4	$p < 0.001$; ↓
		**Peak hip extension angle throughout gait cycle** (˚, negative values correspond with hip extension)		
Fullenkamp et al. [48]	Peak hip extension (˚)	-12.7 ± 5.1	-13.0 ± 6.0	n.s.; ↔
Schache et al. [19]	Peak hip extension (˚)	Left: -20.5 ± 5.6<br>Right: -18.9 ± 5.5	Left: -17.8 ± 4.2<br>Right: -16.9 ± 5.0	$p = 0.023$; ↑<br>$p = 0.030$; ↑
Wank et al. [69]	Peak hip extension (˚)*†	4.0 m/s: -10.87 ± 3.93<br>6.0 m/s: -16.65 ± 3.24	4.0 m/s: -19.49 ± 3.70<br>6.0 m/s: -21.5 ± 3.47	-<br>-
		**Hip angle at toe-off** (˚, negative values correspond with hip extension)		
Firminger et al. [56]	Hip angle at toe-off (˚)	10 km/h: -11.96 ± 5.64<br>13 km/h: -14.80 ± 5.66	10 km/h: -13.35 ± 5.53<br>13 km/h: -15.90 ± 5.56	$p = 0.62$; ↔<br>$p = 0.70$; ↔
Schache et al. [19]	Peak hip extension at toe-off (˚)	Left: -18.1 ± 7.4<br>Right: -16.9 ± 5.9	Left: -12.0 ± 5.1<br>Right: -10.9 ± 5.6	$p = 0.003$; ↑<br>$p < 0.001$; ↑
		**Other hip kinematics sagittal plane**		
Firminger et al. [56]	Hip flexion at mid-stance (˚)	10 km/h: 11.55 ± 6.19<br>13 km/h: 14.48 ± 5.49	10 km/h: 13.49 ± 6.07<br>13 km/h: 15.56 ± 5.38	$p = 0.53$; ↔<br>$p = 0.70$; ↔
Schache et al. [19]	Hip flexion-extension excursion during gait cycle (˚)	Left: 70.4 ± 7.8<br>Right: 70.9 ± 6.3	Left: 68.8 ± 7.5<br>Right: 68.2 ± 6.3	$p = 0.266$; ↔<br>$p = 0.040$; ↑

Email corresponding author: basvanhooren@hotmail.com. Department of Nutrition and Movement Sciences, NUTRIM School of Nutrition and Translational Research in Metabolism, Maastricht University Medical Centre+, Maastricht, The Netherlands

Schache et al. [19]	Time at max hip flexion at loading response (% stride duration)	Left: 7.8 ± 1.4<br>Right: 7.2 ± 1.1	Left: 8.3 ± 0.9‡<br>Right: 7.1 ± 2.2	p = 0.334; ↔<br>p = 0.666; ↔
Schache et al. [19]	Time at max hip extension (% stride duration)	Left: 37.8 ± 5.8<br>Right: 39.4 ± 4.5	Left: 42.8 ± 7.2‡<br>Right: 40.6 ± 5.6	p = 0.152; ↔<br>p = 0.173; ↔
Schache et al. [19]	Time at max hip flexion (% stride duration)	Left: 81.2 ± 3.8<br>Right: 80.2 ± 4.2	Left: 79.7 ± 2.8<br>Right: 78.8 ± 3.0	p = 0.350; ↔<br>p = 0.205; ↔
Sinclair et al. [66]	Hip flexion excursion from foot strike to peak angle (load acceptance) (˚)	1.45 ± 2.0	2.2 ± 2.7	p = 0.687; ↔
Sinclair et al. [66]	Peak flexion velocity in sagittal plane (˚/s)	15.0 ± 109.2	-49.3 ± 70.0	p = 0.067; ↔
Sinclair et al. [66]	Peak extension velocity in sagittal plane (˚/s)	-288.4 ± 115.7	-399.2 ± 52.3	p = 0.035; ↔
	**Frontal plane**			
		**Hip add- and abduction at footstrike** (˚, positive values correspond to hip adduction)		
Fellin et al. [55]	Hip adduction at footstrike (˚)	12.3 ± 4.2	11.3 ± 4.6	-
Schache et al. [19]	Hip adduction at footstrike (˚)	Left: 6.4 ± 2.2<br>Right: 7.3 ± 2.0	Left: 7.2 ± 2.5<br>Right: 5.5 ± 2.5	p = 0.344; ↔<br>p = 0.089; ↔
Sinclair et al. [66]	Hip adduction at footstrike (˚)	6.1 ± 4.5	5.7 ± 3.4	p = 0.816; ↔
		**Peak hip add- and abduction during stance** (˚, positive values correspond to hip adduction)		
Fellin et al. [55]	Peak hip adduction during stance (˚)	18.7 ± 4.3	18 ± 4.4	-

Email corresponding author: basvanhooren@hotmail.com. Department of Nutrition and Movement Sciences, NUTRIM School of Nutrition and Translational Research in Metabolism, Maastricht University Medical Centre+, Maastricht, The Netherlands

Riley et al. [63]	Peak hip adduction during stance (˚)	12.7 ± 4.0	12.1 ± 4.1	*p* = 0.041; ↔
Schache et al. [19]	Peak hip adduction during stance (˚)	Left: 12.8 ± 2.8<br>Right: 10.4 ± 3.7	Left: 11.5 ± 4.6<br>Right: 10.4 ± 4.2	*p* = 0.37; ↔<br>*p* = 0.999; ↔
Sinclair et al. [66]	Peak hip adduction during stance (˚)	11.7 ± 5.7	11.2 ± 3.8	*p* = 0.774; ↔
		**Hip add- and abduction range of motion** (˚, positive values correspond to hip adduction)		
Schache et al. [19]	Hip adduction ROM throughout gait cycle (˚)	Left: 26.2 ± 4.7<br>Right: 25.6 ± 4.8	Left: 25 ± 6.0<br>Right: 24 ± 4.9	*p* = 0.459; ↔<br>*p* = 0.338; ↔
Sinclair et al. [66]	Hip adduction ROM(˚) from footstrike to toe-off	1.0 ± 7.6	4 ± 5.4	*p* = 0.064; ↔
Sinclair et al. [66]	Hip adduction excursion from footstrike to peak angle (˚)	5.6 ± 7.4	5.5 ± 2.8	*p* = 0.037; ↔
		**Other hip kinematics frontal plane** (positive values correspond with hip adduction)		
Schache et al. [19]	Hip abduction at toe-off (˚)	Left: -4.6 ± 3.7<br>Right: -3.5 ± 2.9	Left: -5.5 ± 3.0<br>Right: -3.8 ± 3.5	*p* = 0.439; ↔<br>*p* = 0.612; ↔
Schache et al. [19]	Max swing phase hip abduction (˚)	Left: 13.4 ± 3.5<br>Right: 15.2 ± 2.0	Left: 13.5 ± 2.6<br>Right: 13.7 ± 3.3	*p* = 0.875; ↔<br>*p* = 0.159; ↔
Schache et al. [19]	Time at maximum stance phase hip adduction (% stride duration)	Left: 61.9 ± 1.7<br>Right: 61.1 ± 1.4	Left: 60.7 ± 1.7<br>Right: 60.4 ± 2.5	*p* = 0.174; ↔<br>*p* = 0.209; ↔
Schache et al. [19]	Time at max swing phase hip abduction left (% stride duration)	Left: 9.5 ± 1.8‡<br>Right: 10.3 ± 2.4	Left: 10.0 ± 0.9‡<br>Right: 10.2 ± 1.5	*p* = 0.196; ↔<br>*p* = 0.885; ↔

Email corresponding author: basvanhooren@hotmail.com. Department of Nutrition and Movement Sciences, NUTRIM School of Nutrition and Translational Research in Metabolism, Maastricht University Medical Centre+, Maastricht, The Netherlands

Sinclair et al. [66]	Peak hip adduction velocity in coronal plane (˚/s)	122.0 ± 34.9	139.9 ± 53.2	*p* = 0.410; ↔
Sinclair et al. [66]	Peak hip abduction velocity in coronal plane (˚/s)	-99.7 ± 50.9	-140 ± 64.2	*p* = 0.204; ↔
	**Transverse plane**			
		**Hip transverse plane kinematics** (positive values correspond with internal rotation)		
Fellin et al. [55]	Hip internal rotation (˚)	At foot strike: 5.7 ± 7.2<br>Peak during stance: 7.1 ± 7.1	At foot strike: 6.2 ± 6.0<br>Peak during stance: 7.8 ± 6.3	-<br>-
Sinclair et al. [66]	Hip internal rotation (˚)	At footstrike: -11.3 ± 11.7<br>Peak during stance: -20.1 ± 1 3.7	-5.7 ± 15.3<br>-16 ± 16.5	*p* = 0.034; ↔<br>*p* = 0.267; ↔
Riley et al. [63]	Peak hip internal rotation throughout the gait cycle (˚)	13.0 ± 13.4	14.3 ± 12.8	*p* = 0.041; ↔
Riley et al. [63]	Peak hip external rotation throughout gait cycle (˚)	13.2 ± 12.4	15.0 ± 12.4	*p* = 0.049; ↔
Sinclair et al. [66]	Peak internal rotation velocity (˚/s)	93.2 ± 83.4	77.5 ± 80.0	*p* = 0.208; ↔
		**Other hip transverse plane variables**		
Schache et al. [19]	Transverse plane ROM (% stride duration)	Left: 29.1 ± 6.6<br>Right: 28.7 ± 5.3	Left: 31.1 ± 5.2<br>Right: 30.9 ± 5.3	*p* = 0.243; ↔<br>*p* = 0.172; ↔
Sinclair et al. [66]	ROM in transverse plane across stance phase (˚)	0.4 ± 13.5	8.4 ± 5.4	*p* = 0.008; ↓
Sinclair et al. [66]	Excursion from footstrike to peak angle in transverse plane (˚)	8.9 ± 6.8	10.3 ± 4.9	*p* = 0.232; ↔

Email corresponding author: basvanhooren@hotmail.com. Department of Nutrition and Movement Sciences, NUTRIM School of Nutrition and Translational Research in Metabolism, Maastricht University Medical Centre+, Maastricht, The Netherlands

Sinclair et al. [66]	Peak external rotation velocity in transverse plane (˚/s)	-45.5 ± 77.0	-139.7 ± 50.9	*p* = 0.060; ↔
		**Pelvic in- and external rotation** (˚, positive value corresponds to internal pelvic rotation)		
Riley et al. [63]	Peak pelvic rotation throughout gait cycle (˚)	8.5 ± 3.0	7.6 ± 3.5	*p* = 0.019; ↔
Schache et al. [19]	Pelvic rotation at footstrike (˚)	Left: 3.8 ± 2.6<br>Right: 2.3 ± 2.7	Left: 2.7 ± 2.8<br>Right: 0.7 ± 3.1	*p* = 0.193; ↔<br>*p* = 0.142; ↔
Schache et al. [19]	Pelvic rotation at toe-off (˚)	Left: -0.1 ± 2.3<br>Right: -0.8 ± 2.7	Left: 1.0 ± 2.1<br>Right: -0.2 ± 2.8	*p* = 0.128; ↔<br>*p* = 0.467; ↔
Schache et al. [19]	Peak internal pelvic rotation (% stride duration)	Left: 7.2 ± 2.7<br>Right: 7.8 ± 2.3	Left: 5.8 ± 3.2<br>Right: 8.0 ± 3.3	*p* = 0.060; ↔<br>*p* = 0.867; ↔
		**Pelvic elevation and depression** (˚, positive values correspond with pelvic elevation)		
Schache et al. [19]	Frontal plane pelvic position at foot strike (˚)	Left: -3.1 ± 1.9<br>Right: -3.4 ± 2.4	Left: -2.7 ± 2.1<br>Right: -3.7 ± 2.8	*p* = 0.562; ↔<br>*p* = 0.758; ↔
Schache et al. [19]	Peak frontal plane pelvic position during stance (˚)	Left: -7.6 ± 1.6<br>Right: -6.9 ± 2.6	Left: -8.3 ± 3.8<br>Right: -6.6 ± 2.7	*p* = 0.498; ↔<br>*p* = 0.623; ↔
Schache et al. [19]	Pelvic frontal plane position at toe-off (˚)	Left: +5.8 ± 2.4<br>Right: +5.3 ± 2.1	Left: +6.9 ± 2.6<br>Right: +5.1 ± 2.4	*p* = 0.201; ↔<br>*p* = 0.720; ↔
Schache et al. [19]	Peak frontal plane position during swing (˚)	Left: 6.9 ± 2.6<br>Right: 7.5 ± 1.7	Left: 5.8 ± 2.2<br>Right: 7.8 ± 2.6	*p* = 0.099; ↔<br>*p* = 0.752; ↔
		**Pelvic anterior and posterior tilt** (˚, negative values correspond with anterior pelvic tilt)		
Schache et al. [19]	Anterior pelvic tilt at footstrike (˚)	Left: -13.2 ± 4.0<br>Right: -13.3 ± 3.9	Left: -15.3 ± 4.4<br>Right: -15.0 ± 4.1	*p* = 0.024; ↓<br>*p* = 0.023; ↓

Email corresponding author: basvanhooren@hotmail.com. Department of Nutrition and Movement Sciences, NUTRIM School of Nutrition and Translational Research in Metabolism, Maastricht University Medical Centre+, Maastricht, The Netherlands

Schache et al. [19]	peak anterior pelvic tilt throughout gait cycle (˚)	Left: -18.6 ± 3.6<br>Right: -18.6 ± 3.5	Left: -20.6 ± 2.9<br>Right: -20.3 ± 3.6	$p = 0.063$; ↔<br>$p = 0.028$; ↓
		**Other transverse plane pelvic kinematics** (positive value corresponds to internal pelvic rotation)		
Schache et al. [19]	Time at first max upward pelvic obliquity (% stride duration)	Left: 60.7 ± 2.9<br>Right: 60.0 ± 1.83‡	Left: 61.2 ± 1.9<br>Right: 59.1 ± 3.3	$p = 0.634$; ↔<br>$p = 0.393$; ↔
Schache et al. [19]	Time at first max downward pelvic obliquity (% stride duration)	Left: 9.8 ± 2<br>Right: 10.8 ± 1.8	Left: 9.5 ± 2.3<br>Right: 10.3 ± 1.8	$p = 0.565$; ↔<br>$p = 0.440$; ↔
Schache et al. [19]	Time at first maximum anterior pelvic tilt (% stride duration)	Left: 35 ± 5.6<br>Right: 36.3 ± 2.7	Left: 36.4 ± 5.3<br>Right: 35.6 ± 5.3	$p = 0.285$; ↔<br>$p = 0.877$; ↔
Schache et al. [19]	Time at maximum pelvic internal rotation (% stride duration)	Left: 60.3 ± 5.6<br>Right: 59.8 ± 6.6	Left: 64.0 ± 5.9‡<br>Right: 63.8 ± 15.4‡	$p = 0.135$; ↔<br>$p = 0.514$; ↔
		**Vertical displacement** (cm)		
Firminger et al. [56]	Vertical displacement sacral marker (cm)	10 km/h: -<br>13 km/h: 11.0 ± 2.0	10 km/h: -<br>13 km/h: 11.0 ± 2.0	$p > 0.05$; ↔<br>$p = 0.64$; ↔
Pink et al. [62]	Vertical displacement (cm)	2.95 m/s: 11.2 ± 1.2<br>3.94 m/s: 10.7 ± 2.2	2.95 m/s: 13.8 ± 2.8<br>3.94 m/s: 14.1 ± 2.4	$p < 0.05$<br>$p < 0.05$
Wank et al. [69]	Vertical displacement center of gravity (cm)*	4.0 m/s: 9.76 ± 1.48<br>6.0 m/s: 7.04 ± 1.71	4.0 m/s: 10.52 ± 1.36<br>6.0 m/s: 8.55 ± 1.69	-<br>-
		**Vertical velocity (m/s)**		
Nelson et al. [49]	Vertical velocity center of gravity (m/s)	3.35 m/s: Not reported/provided<br>4.88 m/s: Not reported/provided<br>6.4 m/s: Not reported/provided	3.35 m/s: Not reported/provided<br>4.88 m/s: Not reported/provided<br>6.4 m/s: Not reported/provided	-<br>-<br>-

Email corresponding author: basvanhooren@hotmail.com. Department of Nutrition and Movement Sciences, NUTRIM School of Nutrition and Translational Research in Metabolism, Maastricht University Medical Centre+, Maastricht, The Netherlands

		**Variability of vertical velocity (m/s)**		
Nelson et al. [49]	Variability of vertical velocity of center of gravity (m/s)	3.35 m/s: Not reported/provided<br>4.88 m/s: Not reported/provided<br>6.4 m/s: Not reported/provided	3.35 m/s: Not reported/provided<br>4.88 m/s: Not reported/provided<br>6.4 m/s: Not reported/provided	$p < 0.01$; ↓<br>$p < 0.01$; ↓<br>$p < 0.01$; ↓
Wank et al. [69]	Vertical speed variance of center of gravity (m/s)	4.0 m/s: 0.42 ± 0.07<br>6.0 m/s: 0.49 ± 0.05	4.0 m/s: 0.56 ± 0.14<br>6.0 m/s: 0.68 ± 0.11	$p = 0.103$; ↔<br>$p < 0.001$; ↓
		**Variability of horizontal velocity (ms)**		
Nelson et al. [49]	Variability of horizontal velocity of center of gravity (m/s)	3.35 m/s: Not reported/provided<br>4.88 m/s: Not reported/provided<br>6.4 m/s: Not reported/provided	3.35 m/s: Not reported/provided<br>4.88 m/s: Not reported/provided<br>6.4 m/s: Not reported/provided	$p < 0.01$; ↓<br>$p < 0.01$; ↓<br>$p < 0.01$; ↓
Wank et al. [69]	Horizontal speed variance of center of gravity (m/s)	4.0 m/s: 1.80 ± 0.27<br>6.0 m/s: 1.56 ± 0.25	4.0 m/s: 1.89 ± 0.23<br>6.0 m/s: 1.74 ± 29	$p = 0.006$; ↓<br>$p = 0.015$; ↓
		**Trend symmetry values (unitless)[a]**		
Fellin et al. [55]	Hip sagittal	0.98 ± 0.00		n.a.
Fellin et al. [55]	Hip frontal	0.98 ± 0.00		n.a.
Fellin et al. [55]	Hip transverse	0.9 ± 0.009		n.a.
		**Range offset (°degrees)[b]**		
Fellin et al. [55]	Hip sagittal	0.7 ± 2.7		n.a.
Fellin et al. [55]	Hip frontal	-0.7 ± 2.3		n.a.
Fellin et al. [55]	Hip transverse	0.2 ± 1.8		n.a.
		**Range amplitude (unitless)[c]**		
Fellin et al. [55]	Hip sagittal	1.00 ± 0.08		n.a.
Fellin et al. [55]	Hip frontal	1.06 ± 0.14		n.a.
Fellin et al. [55]	Hip transverse	1.05 ± 0.43		n.a.
		**Phase offset (%)[d]**		

Email corresponding author: basvanhooren@hotmail.com. Department of Nutrition and Movement Sciences, NUTRIM School of Nutrition and Translational Research in Metabolism, Maastricht University Medical Centre+, Maastricht, The Netherlands

Fellin et al. [55]		0 ± 0	n.a.
	Hip sagittal		
Fellin et al. [55]		n/a	n.a.
	Hip frontal		
Fellin et al. [55]	Hip transverse	n/a	n.a.

ROM, range of motion;
[a] Indicates how similar the trends are with values closer to 1 indicating higher similarities; [b] indicates the offset in the trend (positive = overground larger than treadmill; [c] > 1.0 indicates that overground excursions are larger than treadmill; ; [d] indicates the offset, with positive values indicating that overground shifted forward relative to stance time with respect to the treadmill curve.
*Data extracted using WebPlotDigitizer; ‡ Mean and SD estimated based on median and range for small sample sizes as detailed by Hozo et al. [101]; † Converted to similar values reported by other studies by subtracting with 180˚.

Email corresponding author: basvanhooren@hotmail.com. Department of Nutrition and Movement Sciences, NUTRIM School of Nutrition and Translational Research in Metabolism, Maastricht University Medical Centre+, Maastricht, The Netherlands

Table SV. Kinetic outcome measures

Study	Outcome	Treadmill mean ± SD	Overground mean ± SD	P-value (when available); arrow indicating direction of effect compared to overground
		**Pressure variables**		
		**Total foot peak pressure**		
Asmussen et al. [28]	Peak impact force (N)	Quinton TM		
		Insole at 2.7 m/s: 925 ± 219	Insole at 2.7 m/s: 862 ± 226	-
		Force plate at 2.7 m/s: 1642 ± 380	Force plate at 2.7 m/s: 990 ± 243	-
		Insole at 3.6 m/s: 1115 ± 230	Insole at 3.6 m/s: 1051 ± 206	-
		Force plate at 3.6 m/s: 2017 ± 510	Force plate at 3.6 m/s: 1176 ± 203	-
		Insole at 4.5 m/s: 1257 ± 226	Insole at 4.5 m/s: 1232 ± 192	-
		Force plate at 4.5 m/s: 2376 ± 519	Force plate at 4.5 m/s: 1359 ± 200	-
		Healthrider TM		
		Insole at 2.7 m/s: 1026 ± 289		-
		Force plate at 2.7 m/s: 1156 ± 227		-
		Insole at 3.6 m/s: 1128 ± 203		-
		Force plate at 3.6 m/s: 1368 ± 261		-
		Insole at 4.5 m/s: 1315 ± 245		-
		Force plate at 4.5 m/s: 1656 ± 314		-
		Bertec TM		
		Insole at 2.7 m/s: 965 ± 238		-
		Force plate at 2.7 m/s: 1077 ± 233		-
		Insole at 3.6 m/s: 1121 ± 272		-
		Force plate at 3.6 m/s: 1301 ± 317		-
		Insole at 4.5 m/s: 1361 ± 331		-
		Force plate at 4.5 m/s: 1551 ± 365		-
Baur et al. [51]	Plantar peak pressure (kPa)⁑	310 ± 33	386 ± 47	n.s.; ↔
Fu et al. [7]	Plantar peak pressure (kPa/BW)	Non-cushioned TM: 63.3 ± 6.2<br>Cushioned TM: 64.7 ± 7.4	Concrete: 67.6 ± 9.9<br>Synthetic track: 70.8 ± 8.5<br>Grass: 69.1 ± 7.5	All comparisons n.s.; ↔

Email corresponding author: basvanhooren@hotmail.com. Department of Nutrition and Movement Sciences, NUTRIM School of Nutrition and Translational Research in Metabolism, Maastricht University Medical Centre+, Maastricht, The Netherlands

Hong et al. [27]	Total foot peak plantar pressure (kPa)	381.2 ± 83.1	Concrete: 454 ± 82.3<br>Natural grass: 395.9 ± 75.1	$p < .0002$; ↓<br>n.s.; ↔
			**Fore foot peak pressure**	
Baur et al. [51]	Fore foot peak plantar pressure (N/cm^2)*†	30.6 ± 5.6	38.5 ± 5.6	n.s.; ↔
Fu et al. [7]	Fore foot peak plantar pressure (kPa)*	Non-cushioned TM: 26.2 ± 5.0<br>Cushioned TM: 28.5 ± 5.2	Concrete: 25.9 ± 5.2<br>Synthetic track: 30.0 ± 7.3<br>Grass: 29.7 ± 5.8	All comparisons n.s.; ↔
Garcia-Perez et al. [6]	Medial metatarsal peak pressure (kPa)*	3.33 m/s: 121.4 ± 17.3<br>4.0 m/s: 119.7 ± 13.9	3.33 m/s: 138.7 ± 22.5<br>4.0 m/s: 152.6 ± 20.8	3.33 m/s: n.s.; ↔<br>4.0 m/s: $p < .05$; ↓
Garcia-Perez et al. [6]	Lateral metatarsal peak pressure (kPa)*	3.33 m/s: 71.1 ± 20.8<br>4.0 m/s: 52.0 ± 24.3	3.33 m/s: 74.6 ± 19.1<br>4.0 m/s: 72.8 ± 20.8	3.33 m/s: n.s.; ↔<br>4.0 m/s: n.s.; ↔
Hong et al. [27]	Medial forefoot peak pressure (kPa)	302.2 ± 78.1	Concrete: 336.9 ± 80<br>Natural grass: 331.2 ± 82.4	n.s.; ↔<br>n.s.; ↔
Hong et al. [27]	Central forefoot peak pressure (kPa)	350.8 ± 82.3	Concrete: 369.7 ± 96.8<br>Natural grass: 331.5 ± 83.5	n.s.; ↔<br>n.s.; ↔
Hong et al. [27]	Lateral forefoot peak pressure (kPa)	237.4 ± 89.6	Concrete: 300.3 ± 72.3<br>Natural grass: 247.6 ± 58.8	n.s.; ↔<br>n.s.; ↔
			**Rearfoot peak pressure**	
Baur et al. [51]	Rearfoot plantar peak pressure (N/cm^2)	17.1 ± 6.0	19.4 ± 6.5	n.s.; ↔
Fu et al. [7]	Heel peak plantar pressure (kPa)*	Non-cushioned TM: 114.2 ±17.2<br>Cushioned TM: 106.0 ± 15.4	Concrete: 117.4 ± 16.3<br>Synthetic track: 111.3 ± 15.7<br>Grass: 112.1 ± 15.1	Cushioned TM vs concrete: $p = 0.039$; ↓<br>Other comparisons n.s.; ↔
			**Medial heel peak pressure**	
Garcia-Perez et al. [57]	Medial heel peak pressure (kPa)*	3.33 m/s: 256.6 ± 20.8<br>4.0 m/s: 291.3 ± 31.2	3.33 m/s: 369.4 ± 31.2<br>4.0 m/s: 431.8 ± 39.9	3.33 m/s: $p < .05$; ↓<br>4.0 m/s: $p < .05$; ↓

Email corresponding author: basvanhooren@hotmail.com. Department of Nutrition and Movement Sciences, NUTRIM School of Nutrition and Translational Research in Metabolism, Maastricht University Medical Centre+, Maastricht, The Netherlands

Hong et al. [27]	Medial heel peak pressure (kPa)	233.3 ± 50	Concrete: 245.8 ± 106.9<br>Natural grass: 214.2 ± 52.0	n.s.; ↔<br>n.s.; ↔
		**Lateral heel peak pressure**		
Garcia-Perez et al. [57]	Lateral heel peak pressure (kPa)*	3.33 m/s: 176.9 ± 19.1<br>4.0 m/s: 225.4 ± 26.0	3.33 m/s: 251.4 ± 26.0<br>4.0 m/s: 317.3 ± 32.9	3.33 m/s: $p < .05$; ↓<br>4.0 m/s: $p < .05$; ↓
Hong et al. [27]	Lateral heel peak pressure (kPa)	230.0 ± 47.9	Concrete: 248.8 ± 114.8<br>Natural grass: 207.0 ± 50.4	n.s.; ↔<br>n.s.; ↔
		**Medial arch peak pressure**		
Fu et al. [7]	Medial peak plantar pressure (kPa)*	Non-cushioned TM: 52.4 ± 8.7<br>Cushioned TM: 52.7 ± 8.4	Concrete: 54.2 ± 9.6<br>Synthetic track: 53.9 ± 9.6<br>Grass: 55.0 ± 9.3	All comparisons n.s.; ↔
Garcia-Perez et al. [57]	Medial arch peak pressure (kPa)*	3.33 m/s: 76.3 ± 12.1<br>4.0 m/s: 97.1 ± 13.9	3.33 m/s: 76.3 ± 12.1<br>4.0 m/s: 91.1 ± 13.9	3.33 m/s: n.s.; ↔<br>4.0 m/s: n.s.; ↔
Hong et al. [27]	Medial midfoot peak pressure (kPa)	111.6 ± 20	Concrete: 128.7 ± 29.1<br>Natural grass: 126.0 ± 28.9	n.s.; ↔<br>n.s.; ↔
		**Lateral arch peak pressure**		
Fu et al. [7]	Lateral peak plantar pressure (kPa)*	Non-cushioned TM: 49.5 ± 8.7<br>Cushioned TM: 48.3 ± 8.4	Concrete: 51.3 ± 8.7<br>Synthetic track: 50.1 ± 7.6<br>Grass: 48.3 ± 7.9	All comparisons n.s.; ↔
Garcia-Perez et al. [57]	Lateral arch peak pressure (kPa)*	3.33 m/s: 31.2 ± 8.7<br>4.0 m/s: 34.7 ± 8.7	3.33 m/s: 31.2 ± 8.7<br>4.0 m/s: 36.4 ± 6.9	3.33 m/s: n.s.; ↔<br>4.0 m/s: n.s.; ↔
Hong et al. [27]	Lateral midfoot peak pressure (kPa)	137.2 ± 38.8	Concrete: 160.5 ± 59.1<br>Natural grass: 143.3 ± 46.3	n.s.; ↔<br>n.s.; ↔
		**Greater toe peak pressure**		
Garcia-Perez et al. [57]	Hallux peak pressure (kPa)*	3.33 m/s: 121.4 ± 20.8<br>4.0 m/s: 126.6 ± 24.3	3.33 m/s: 189.0 ± 29.5<br>4.0 m/s: 202.9 ± 34.7	3.33 m/s: $p < .05$; ↓<br>4.0 m/s: $p < .05$; ↓

Email corresponding author: basvanhooren@hotmail.com. Department of Nutrition and Movement Sciences, NUTRIM School of Nutrition and Translational Research in Metabolism, Maastricht University Medical Centre+, Maastricht, The Netherlands

Hong et al. [27]	Greater toe peak pressure (kPa)	296.3 ± 63.9	Concrete: 384.0 ± 109.0<br>Natural grass: 362.0 ± 82.3	*p* < .0002; ↓<br>*p* < .0002; ↓
			**Lesser toes peak pressure**	
Garcia-Perez et al. [57]	Lesser toes peak pressure (kPa)*	3.33 m/s: 45.1 ± 6.9<br>4.0 m/s: 52.0 ± 8.7	3.33 m/s: 32.9 ± 10.4<br>4.0 m/s: 52.0 ± 8.7	3.33 m/s: n.s.; ↔<br>4.0 m/s: n.s.; ↔
Hong et al. [27]	Lesser toes peak pressure (kPa)	170.8 ± 30.2	Concrete: 234.6 ± 61.4<br>Natural grass: 231.8 ± 62.9	*p* < .0002; ↓<br>*p* < .0002; ↓
			**Other pressure variables**	
Fu et al. [7]	First peak pressure (kPa)	Non-cushioned TM: 42.0 ± 4.3<br>Cushioned TM: 37.5 ± 4.5	Concrete: 42.8 ± 3.9<br>Synthetic track: 37.1 ± 4.6<br>Grass: 40.8 ± 4.3	Cushioned TM vs concrete: *p* = 0.045; ↓<br>Other comparisons n.s.; ↔
Fu et al. [7]	Time to first peak pressure (ms)	Non-cushioned TM: 39.3 ± 3.5<br>Cushioned TM: 45.7 ± 4.4	Concrete: 36.7 ± 6.2<br>Synthetic track: 43.3 ± 5.3<br>Grass: 42.4 ± 4.8	All comparisons n.s.; ↔
Fu et al. [7]	Time to second peak pressure (ms)	Non-cushioned TM: 100.7 ± 3.9<br>Cushioned TM: 101.9 ± 4.2	Concrete: 100.2 ± 16.4<br>Synthetic track: 102.4 ± 14.2<br>Grass: 101.2 ± 7.2	All comparisons n.s.; ↔
Fu et al. [7]	Pressure-time integral for impact phase (kPa x ms)	Non-cushioned TM: 961.0 ± 121.9<br>Cushioned TM: 796.3 ± 133.4	Concrete: 1000.6 ± 334.9<br>Synthetic track: 922.3 ± 283.6<br>Grass: 911.8 ± 365.1	Cushioned TM vs concrete: *p* = 0.038; ↓<br>Other comparisons n.s.; ↔
Fu et al. [7]	Pressure-time integral for entire contact phase (kPa x ms)	Non-cushioned TM: 9116.3 ± 2755.6<br>Cushioned TM: 8776.4 ± 3537.6	Concrete: 9186.2 ± 2558.3<br>Synthetic track: 9049.3 ± 2807.9<br>Grass: 8787.5 ± 2049.9	All comparisons n.s.; ↔
Fu et al. [7]	Mid foot peak plantar pressure (kPa)*	Non-cushioned TM: 32.9 ± 6.7<br>Cushioned TM: 33.8 ± 7.6	Concrete: 35.8 ± 7.0<br>Synthetic track: 33.8 ± 7.9<br>Grass: 35.5 ± 7.6	All comparisons n.s.; ↔
Garcia-Perez et al. [57]	Central metatarsal peak pressure (kPa)*	3.33 m/s: 232.4 ± 31.2<br>4.0 m/s: 227.2 ± 26.0	3.33 m/s: 261.8 ± 29.5<br>4.0 m/s: 265.3 ± 27.7	3.33 m/s: n.s.; ↔<br>4.0 m/s: n.s.; ↔
			**Pressures as % body weight**	

Email corresponding author: basvanhooren@hotmail.com. Department of Nutrition and Movement Sciences, NUTRIM School of Nutrition and Translational Research in Metabolism, Maastricht University Medical Centre+, Maastricht, The Netherlands

Hong et al. [27]	Total foot peak plantar pressure (% body weight)	235.4 ± 36.9	Concrete: 267.3 ± 33.2<br>Natural grass: 263.1 ± 44.1	$p < .0002$; ↓<br>$p < .0002$; ↓
Hong et al. [27]	Media head peak pressure (% body weight)	57.3 ± 18.4	Concrete: 61.5 ± 25.9<br>Natural grass: 54.2 ± 16.6	n.s.; ↔<br>n.s.; ↔
Hong et al. [27]	Lateral head peak pressure (% body weight)	49.4 ± 15.4	Concrete: 53.0 ± 23.7<br>Natural grass: 44.8 ± 14.8	n.s.; ↔<br>n.s.; ↔
Hong et al. [27]	Medial midfoot peak pressure (% body weight)	27.7 ± 8.8	Concrete: 31.7 ± 9.4<br>Natural grass: 31.3 ± 10.0	n.s.; ↔<br>n.s.; ↔
Hong et al. [27]	Lateral midfoot peak pressure (% body weight)	38.9 ± 8.4	Concrete: 45 ± 11.0<br>Natural grass: 42 ± 11.4	n.s.; ↔<br>n.s.; ↔
Hong et al. [27]	Medial forefoot peak pressure (% body weight)	37.2 ± 8.1	Concrete: 46.6 ± 9.2<br>Natural grass: 46.6 ± 10.3	$p < .0002$; ↓<br>$p < .0002$; ↓
Hong et al. [27]	Central forefoot peak pressure (% body weight)	53.9 ± 11.7	Concrete: 59.1 ± 13.6<br>Natural grass: 56.0 ± 12.0	n.s.; ↔<br>n.s.; ↔
Hong et al. [27]	Lateral forefoot peak pressure (% body weight)	31.3 ± 9.7	Concrete: 38.2 ± 9.9<br>Natural grass: 35.2 ± 9.0	n.s.; ↔<br>n.s.; ↔
Hong et al. [27]	Greater toe peak pressure (% body weight)	26.2 ± 3.9	Concrete: 33.6 ± 6.2<br>Natural grass: 32.6 ± 6.2	$p < .0002$; ↓<br>$p < .0002$; ↓
Hong et al. [27]	Lesser toes peak pressure (% body weight)	29.6 ± 4.6	Concrete: 37.8 ± 6.6<br>Natural grass: 36.0 ± 3.4	$p < .0002$; ↓<br>$p < .0002$; ↓
		**Ground reaction forces**		

Email corresponding author: basvanhooren@hotmail.com. Department of Nutrition and Movement Sciences, NUTRIM School of Nutrition and Translational Research in Metabolism, Maastricht University Medical Centre+, Maastricht, The Netherlands

		**Peak vertical ground reaction forces normalized to body weight**		
Firminger et al. [56]	Peak active force (BW)	10 km/h: 2.34 ± 0.21<br>13 km/h: 2.55 ± 0.26	10 km/h: 2.42 ± 0.21<br>13 km/h: 2.61 ± 0.25	*p* = 0.47; ↔<br>*p* = 0.68; ↔
Kluitenberg et al. [40]	Fz2 (BW, max value in vertical GRF normalized to body weight)	HS 11 km/h: 2.53 ± 0.18<br>HS 12.3 km/h: 2.65 ± 0.25<br>HS 14 km/h: 2.70 ± 0.22<br>NHS 11 km/h: 2.55 ± 0.2<br>NHS 12.3 km/h: 2.58 ± 0.13<br>NHS 14 km/h: 2.78 ± 0.2	HS 11 km/h: 2.54 ± 0.20<br>HS 12.3 km/h: 2.70 ± 0.26<br>HS 14 km/h: 2.77 ± 0.24<br>NHS 11 km/h: 2.56 ± 0.17<br>NHS 12.3 km/h: 2.61 ± 0.15<br>NHS 14 km/h: 2.79 ± 0.15	-<br>-<br>-<br>-<br>-<br>-
Pink et al. [62]	Peak vertical force normalized to body weight (%)	2.95 m/s: not reported/provided<br>3.94 m/s: not reported/provided	2.95 m/s: not reported/provided<br>3.94 m/s: not reported/provided	n.s.; ↔<br>n.s.; ↔
Riley et al. [63]	Vertical GRF (BW) ‡	2.49 ± 0.22	2.63 ± 0.45	*p* = 0.021; ↔
		**Average vertical loading rate** (BW s^{-1})		
Chambon et al. [41]	Average vertical Loading rate (BW s^{-1})	Shoe 0 mm drop: 53 ± 23<br>Shoe 4 mm drop: 58 ± 22<br>Shoe 8 mm drop: 66 ± 15	Shoe 0 mm drop: 121 ± 44<br>Shoe 4 mm drop: 110 ± 36<br>Shoe 8 mm drop: 96 ± 28	-<br>-<br>-
Firminger et al. [56]	Average vertical Loading rate (BW s^{-1})	10 km/h: 55.05 ± 18.77<br>13 km/h: 72.66 ± 18.94	10 km/h: 52.94 ± 18.42<br>13 km/h: 70.67 ± 18.60	*p* = 0.82; ↔<br>*p* = 0.83; ↔
Kluitenberg et al. [40]	Average vertical loading rate (BW s^{-1})	HS 11 km/h: 73.92 ± 20.22<br>HS 12.3 km/h: 88.7 ± 20.75<br>HS 14 km/h: 100.77 ± 29.10<br>NHS 11 km/h: 31.21 ± 5.01<br>NHS 12.3 km/h: 33.78 ± 4.2<br>NHS 14 km/h: 36 ± 4.2	HS 11 km/h: 68.89 ± 20.26<br>HS 12.3 km/h: 82.14 ± 21.38<br>HS 14 km/h: 90.70 ± 23.66<br>NHS 11 km/h: 33.96 ± 6.07<br>NHS 12.3 km/h: 47.09 ± 22.92<br>NHS 14 km/h: 43.63 ± 13.89	-<br>-<br>-<br>-<br>-<br>-
		**Transient peak** (body weights [BW's])		
Chambon et al. [41]	Transient peak/vertical impact peak (BW's)	Shoe 0 mm drop: 1.23 ± 0.2<br>Shoe 4 mm drop: 1.30 ± 0.28<br>Shoe 8 mm drop: 1.33 ± 0.21	Shoe 0 mm drop: 1.47 ± 0.29<br>Shoe 4 mm drop: 1.47 ± 0.25<br>Shoe 8 mm drop: 1.42 ± 0.22	-<br>-<br>-

Email corresponding author: basvanhooren@hotmail.com. Department of Nutrition and Movement Sciences, NUTRIM School of Nutrition and Translational Research in Metabolism, Maastricht University Medical Centre+, Maastricht, The Netherlands

Kluitenberg et al. [40]	Transient peak/vertical impact peak (BW's)	HS 11 km/h: 1.7 ± 0.23<br>HS 12.3 km/h: 1.93 ± 0.3<br>HS 14 km/h: 2.06 ± 0.32	HS 11 km/h: 1.67 ± 0.26<br>HS 12.3 km/h: 1.94 ± 0.45<br>HS 14 km/h: 1.94 ± 0.25	-<br>-<br>-
		**Instantaneous vertical loading rate** (BW s^{-1})		
Firminger et al. [56]	Instantaneous vertical loading rate (BW s^{-1})	10 km/h: 91.48 ± 22.56<br>13 km/h: 114.91 ± 26.74	10 km/h: 73.76 ± 22.14<br>13 km/h: 95.66 ± 26.25	p = 0.13; ↔<br>p = 0.16; ↔
Kluitenberg et al. [40]	Instantaneous vertical loading rate (IVLR, the steepest part of the vertical RFD curve from stance to impact peak in BW s^{-1})	HS 11 km/h: 87.28 ± 23.39<br>HS 12.3 km/h: 105.33 ± 25.08<br>HS 14 km/h: 118.08 ± 33.73<br>NHS 11 km/h: 65.09 ± 13.74<br>NHS 12.3 km/h: 74.25 ± 16.47<br>NHS 14 km/h: 87.41 ± 18.74	HS 11 km/h: 81.11 ± 25.62<br>HS 12.3 km/h: 95.34 ± 25.67<br>HS 14 km/h: 104.4 ± 29.29<br>NHS 11 km/h: 70.03 ± 14.68<br>NHS 12.3 km/h: 77 ± 22.35<br>NHS 14 km/h: 95.81 ± 26.02	-<br>-<br>-<br>-<br>-<br>-
		**Peak propulsive force** (BW)		
Firminger et al. [56]	Peak propulsive force (BW)	10 km/h: 0.21 ± 0.03<br>13 km/h: 0.30 ± 0.04	10 km/h: 0.25 ± 0.03<br>13 km/h: 0.33 ± 0.04	p = 0.01; ↔<br>p = 0.14; ↔
Riley et al. [63]	Anterior GRF (BW) ‡	0.31 ± 0.05	0.37 ± 0.12	p = 0.001; ↓
		**Other ground reaction force variables**		
Firminger et al. [56]	Vertical impulse (BW·s)	10 km/h: 0.36 ± 0.03<br>13 km/h: 0.35 ± 0.03	10 km/h: 0.39 ± 0.03<br>13 km/h: 0.37 ± 0.03	p = 0.06; ↔<br>p = 0.29; ↔
Firminger et al. [56]	Propulsive impulse (BW·s)	10 km/h: 0.02 ± 0.003<br>13 km/h: 0.02 ± 0.003	10 km/h: 0.02 ± 0.003<br>13 km/h: 0.02 ± 0.003	p = 0.13; ↔<br>p = 0.41; ↔
Firminger et al. [56]	Peak braking force (BW)	10 km/h: -0.27 ± 0.08<br>13 km/h: -0.35 ± 0.10	10 km/h: -0.31 ± 0.09<br>13 km/h: -0.40 ± 0.10	p = 0.09; ↔<br>p = 0.10; ↔
Kluitenberg et al. [40]	tFz1 (time from footstrike to the vertical impact peak in ms)	HS 11 km/h: 35 ± 4.86<br>HS 12.3 km/h: 34 ± 3.35<br>HS 14 km/h: 33 ± 4.88	HS 11 km/h: 35 ± 4.08<br>HS 12.3 km/h: 34 ± 4.42<br>HS 14 km/h: 32 ± 5	-<br>-<br>-
Kluitenberg et al. [40]	tFz2 (time from heelstrike to Fz2 in ms)	HS 11 km/h: 109 ± 10.22<br>HS 12.3 km/h: 100 ± 12.19<br>HS 14 km/h: 96 ± 11.47	HS 11 km/h: 112 ± 13.55<br>HS 12.3 km/h: 102 ± 13.28<br>HS 14 km/h: 99 ± 10	-<br>-<br>-

Email corresponding author: basvanhooren@hotmail.com. Department of Nutrition and Movement Sciences, NUTRIM School of Nutrition and Translational Research in Metabolism, Maastricht University Medical Centre+, Maastricht, The Netherlands

		NHS 11 km/h: 103 ± 15<br>NHS 12.3 km/h: 98 ± 11<br>NHS 14 km/h: 91 ± 10	NHS 11 km/h: 102 ± 13<br>NHS 12.3 km/h: 99 ± 12<br>NHS 14 km/h: 92 ± 8	-<br>-<br>-
Riley et al. [63]	Medial GRF (BW) ‡	0.08 ± 0.03	0.11 ± 0.05	$p < 0.001$; ↓
		**Joint moments** (all expressed as internal moments)		
		**Ankle joint moments** (negative value corresponds to plantarflexion moment)		
Riley et al. [63]	Peak ankle plantarflex moment (Nm/kg)	-4.01 ± 0.56	-3.44 ± 0.7	$p < 0.001$; ↑
Willy et al. [16]	Peak ankle plantarflex moment (Nm/kg)#	-2.68 ± 0.53	-2.34 ± 0.27	$p < 0.001$; ↑
		**Knee joint moments in sagittal plane**		
Riley et al. [63]	Peak knee extension moment (Nm/kg)[1]	1.7 ± 0.51	2.33 ± 0.81	$p = 0.001$; ↓
Willy et al. [16]	Peak knee extension moment (Nm/kg)#	2.08 ± 0.38	2.01 ± 0.53	$p = 0.280$; ↔
		**Other joint moment variables**		
Riley et al. [63]	Peak hip extension moment (Nm/kg)	1.3 ± 0.88	1.74 ± 0.73	$p = 0.004$; ↔
Riley et al. [63]	Peak hip abduction moment (Nm/kg)	0.32 ± 0.2	0.41 ± 0.21	$p = 0.025$; ↔
Riley et al. [63]	Peak knee adduction mom (Nm/kg)	1.54 ± 0.53	1.9 ± 0.57	$p < 0.001$; ↓
		**Eccentric ankle power** (positive values correspond with concentric power)		
Riley et al. [63]	Eccentric Ankle power (W/kg)	-10.38 ± 3.24	-8.00 ± 3.23	$p < 0.001$; ↑
Willy et al. [16]	Eccentric ankle power (W/kg)#	-5.84 ± 1.29	-5.55 ± 1.55	$p = 0.250$; ↔

Email corresponding author: basvanhooren@hotmail.com. Department of Nutrition and Movement Sciences, NUTRIM School of Nutrition and Translational Research in Metabolism, Maastricht University Medical Centre+, Maastricht, The Netherlands

		**Other sagittal plane joint powers** (positive values correspond with concentric power)		
Riley et al. [63]	Eccentric hip power absorption (W/kg)	-4.46 ± 3.57	-6.93 ± 3.81	$p = 0.002$; ↔
Riley et al. [63]	Concentric knee power (W/kg)	7.63 ± 2.54	11.26 ± 4.39	$p < 0.001$; ↓
Riley et al. [63]	Eccentric knee power (W/kg)	-9.47 ± 3.40	-12.12 ± 4.28	$p = 0.002$; ↔
Willy et al. [16]	Concentric ankle power (W/kg.m)	6.19 ± 1.54	4.84 ± 0.75	$p < 0.001$; ↑
Willy et al. [16]	Concentric ankle power (W/kg)	10.89 ± 2.85	8.51 ± 1.48	-
		**Other kinetic variables**		
Meinert et al. [60]	Average dominant frequency of oscillations at the Achilles tendon (Hz)	n.a.	n.a.	-
Meinert et al. [60]	Normalized power spectrum of the distal accelerometer (vertical direction)	n.a.	n.a.	-
Meinert et al. [60]	Normalized power spectrum of the distal accelerometer (horizontal direction)	n.a.	n.a.	-
Willy et al. [16]	Peak patellofemoral joint reaction force (BW)	4.0 ± 1.0	4.0 ± 0.8	$p = 0.990$; ↔
Willy et al. [16]	Peak patellofemoral joint stress (mPA)	6.2 ± 1.4	6.1 ± 1.5	$p = 0.730$; ↔

Email corresponding author: basvanhooren@hotmail.com. Department of Nutrition and Movement Sciences, NUTRIM School of Nutrition and Translational Research in Metabolism, Maastricht University Medical Centre+, Maastricht, The Netherlands

Willy et al. [16]	Patellofemoral joint stress average loading rate (mPA/s)	131.5 ± 26.9	155.6 ± 61.3	$p = 0.110$; ↔
Willy et al. [16]	Patellofemoral joint stress impulse (mPA*s)	0.71 ± 0.22	0.71 ± 0.16	$p = 0.840$; ↔
Willy et al. [16]	Cumulative patellofemoral joint stress (mPA*s/km)	344.5 ± 118.5	324.7 ± 73.3	$p = 0.210$; ↔
Willy et al. [16]	Peak Achilles force (BW)	5.35 ± 0.782	4.68 ± 0.533	$p < 0.001$; ↑
Willy et al. [16]	Achilles loading rate (BW/s)	65.1 ± 10.8	54.7 ± 10.5	$p < 0.001$; ↑
Willy et al. [16]	Achilles impulse (BW.s)	0.66 ± 0.13	0.59 ± 0.08	$p = 0.020$; ↑
		**Accelerations**		
		**Peak tibial acceleration** (g)		
Fu et al. [7]	Peak tibial acceleration (g) with accelerometer on tibial tuberosity	Non-cushioned TM: 11.6 ± 3.0<br>Cushioned TM: 10.3 ± 3.1	Concrete: 12.4 ± 3.1<br>Synthetic track: 10.9 ± 3.5<br>Grass: 11.1 ± 3.4	n.s.; ↔
Garcia-Perez et al. [58]	Peak tibia impact acceleration (g) with accelerometer on the proximal tibia	15.3 ± 6.8	24.6 ± 10.8	$p < .05$; ↓
Montgomery et al. [61]	Peak tibia acceleration (g) with accelerometer on middle of the anterior tibia	2.88 m/s: 5.8 ± 1.4<br>4.28 m/s: 8.7 ± 2.0	2.88 m/s: 5.3 ± 1.5<br>4.28 m/s: 8.4 ± 1.6	$p = 0.053$; ↔<br>$p = 0.778$; ↔
Oliveira et al. [50]	Peak negative acceleration of tibia at	3.55 ± 0.3	3.56 ± 0.4	$p = 0.564$; ↔

Email corresponding author: basvanhooren@hotmail.com. Department of Nutrition and Movement Sciences, NUTRIM School of Nutrition and Translational Research in Metabolism, Maastricht University Medical Centre+, Maastricht, The Netherlands

	foot strike (g), with exact placement on tibia not specified			
		**Other acceleration variables**		
Bigelow et al. [53]	Peak vertical acceleration at L5 (g)*	4.19 ± 0.79	4.15 ± 0.77	0.52; ↔
Bigelow et al. [53]	Peak horizontal acceleration at L5 (g)*	1.50 ± 0.40	1.76 ± 0.46	*p* = 0.001; ↓
Garcia-Perez et al. [58]	Tibia impact rate (g/s) on the proximal tibia	405 ± 215	614 ± 245	*p* < .05; ↓
Garcia-Perez et al. [58]	Head impact acceleration (g)	2.8 ± 0.6	3.2 ± 0.7	*p* < .05; ↓
Garcia-Perez et al. [58]	Head impact rate (g/s)	41 ± 8	41 ± 10	n.s.; ↔
Garcia-Perez et al. [58]	Shock attenuation (%)	75.5 ± 20.8	82.1 ± 9.7	n.s.; ↔
Montgomery et al. [61]	Acceleration gradient (slope from point of ground contact to acceleration peak (m/s)	2.88 m/s: 1559 ± 518<br>4.28 m/s: 2975 ± 1088	2.88 m/s: 1444 ± 636<br>4.28 m/s: 2920 ± 706	*p* = 0.281; ↔<br>*p* = 0.683; ↔
Oliveira et al. [50]	Inter-trial variability of acceleration during stance (%)	25.1 ± 16.8	32.2 ± 20.5	*p* = 0.011 ; ↓
Oliveira et al. [50]	Inter-trial variability of acceleration during pre-landing (%)	50.3 ± 16.7	61.3 ± 28.3	*p* = 0.027 ; ↓

BW, body weight; HS, heel-strike runners; NHS, non-heel strike runners;
*Data extracted using WebPlotDigitizer; [†]SD computed from 95% confidence interval and t-distribution as described by Higgins, Deeks [102]; [‡]converted from % body weight to body weight.
[‡]Converted from N/cm^2 to kPa. [#] Data converted by the author from Nm/m.kg to Nm/kg or W/kg.m to W/kg to facilitate meta-analysis using mean differences rather than standardized mean differences.
[1]This was reported as flexion moment but likely represents an extension moment.

Van Hooren et al. (2019). Biomechanical comparison of treadmill and overground running. Sports Medicine.
Email corresponding author: basvanhooren@hotmail.com. Department of Nutrition and Movement Sciences, NUTRIM School of Nutrition and Translational Research in Metabolism, Maastricht University Medical Centre+, Maastricht, The Netherlands

Email corresponding author: basvanhooren@hotmail.com. Department of Nutrition and Movement Sciences, NUTRIM School of Nutrition and Translational Research in Metabolism, Maastricht University Medical Centre+, Maastricht, The Netherlands

Table SVI. Electromyographic outcome measures

Study	Outcome	Treadmill mean ± SD	Overground mean ± SD	P-value (when available); arrow indicating direction of effect compared to overground
		**Onset muscle activity (% of stride with respect to touchdown)*†**		
Baur et al. [51]	Peroneus longus	-35.9 ± 19.2	-20.6 ± 20.2	*p* = 0.01; ↓
Baur et al. [51]	Soleus	-1.5 ± 3.7	0.0 ± 3.7	n.s.; ↔
Baur et al. [51]	Tibialis anterior	-57.1 ± 11.2	-59.1 ± 8.4	n.s.; ↔
		**Time of max activation in gait cycle (% of stride with respect to touchdown)*†**		
Baur et al. [51]	Peroneus longus	12.7 ± 5.8	19.1 ± 4.6	*p* = 0.01
Baur et al. [51]	Soleus	13.9 ± 3.0	15.2 ± 3.6	n.s.; ↔
Baur et al. [51]	Tibialis anterior	-4.3 ± 4.3	-2.4 ± 3.2	n.s.; ↔
		**Total time of activation (% of stride)**		
Baur et al. [51]	Peroneus longus	Not reported/provided	Not reported/provided	n.s.; ↔
Baur et al. [51]	Soleus	Not reported/provided	Not reported/provided	n.s.; ↔
Baur et al. [51]	Tibialis anterior	Not reported/provided	Not reported/provided	n.s.; ↔
		**Normalized amplitude during pre-activation phase*†**		
Baur et al. [51]	Peroneus longus	1.10 ± 1.1	1.57 ± 1.3	n.s.; ↔
Baur et al. [51]	Soleus	1.26 ± 1.4	1.80 ± 1.6	n.s.; ↔
Baur et al. [51]	Tibialis anterior	0.31 ± 0.4	0.63 ± 0.4	n.s.; ↔
		**Normalized amplitude during weight acceptance phase*†**		
Baur et al. [51]	Peroneus longus	1.77 ± 0.8	1.25 ± 0.9	*p* = 0.001; ↑
Baur et al. [51]	Soleus	2.96 ± 1.3	1.86 ± 1.4	*p* = 0.001; ↑
Baur et al. [51]	Tibialis anterior	0.83 ± 0.3	1.07 ± 0.4	n.s.; ↔

Email corresponding author: basvanhooren@hotmail.com. Department of Nutrition and Movement Sciences, NUTRIM School of Nutrition and Translational Research in Metabolism, Maastricht University Medical Centre+, Maastricht, The Netherlands

		**Normalized amplitude during push-off phase*†**		
Baur et al. [51]	Peroneus longus	1.10 ± 1.1	1.71 ± 1.3	*p* = 0.001; ↓
Baur et al. [51]	Soleus	1.26 ± 1.0	2.71 ± 1.4	*p* = 0.001; ↓
Baur et al. [51]	Tibialis anterior	0.31 ± 0.4	0.65 ± 0.5	n.s.; ↔
		**Amplitude of max activity (%)**		
Montgomery et al. [61]	Rectus femoris	2.88 m/s: 40 ± 18<br>4.28 m/s: 69 ± 20	2.88 m/s: 38 ± 16<br>4.28 m/s: 70 ± 31	*p* = 0.63; ↔<br>*p* = 0.99; ↔
Montgomery et al. [61]	Semitendinosus	2.88 m/s: 66 ± 11<br>4.28 m/s: 89 ± 19	2.88 m/s: 65 ± 11<br>4.28 m/s: 96 ± 19	*p* = 0.97; ↔<br>*p* = 0.54; ↔
Montgomery et al. [61]	Tibialis anterior	2.88 m/s: 74 ± 20<br>4.28 m/s: 95 ± 28	2.88 m/s: 92 ± 1<br>4.28 m/s: 111 ± 31	*p* = 0.003; ↓<br>*p* = 0.39; ↔
Montgomery et al. [61]	Soleus	2.88 m/s: 73 ± 14<br>4.28 m/s: 77 ± 11	2.88 m/s: 81 ± 25<br>4.28 m/s: 91 ± 16	*p* = 0.001; ↓<br>*p* = 0.001; ↓
		**Amplitude of max activity (%)**		
Sedighi et al. [65]	Rectus Femoris	First 50% stance: 23.9 ± 8.7<br>Final 50% stance: 6.9 ± 4.1	First 50% stance: 39.2 ± 17.6<br>Final 50% stance: 18.4 ± 12.7	*p* = 0.001; ↓<br>*p* = 0.001; ↓
Sedighi et al. [65]	Vastus Medialis	First 50% stance: 40.0 ± 17.8<br>Final 50% stance: 20.8 ± 10.5	First 50% stance: 59.3 ± 32.8<br>Final 50% stance: 26.3 ± 31.3	*p* = 0.006; ↓<br>*p* = 0.01; ↔
Sedighi et al. [65]	Vastus lateralis	First 50% stance: 35.1 ± 10.0<br>Final 50% stance: 11.2 ± 7.5	First 50% stance: 56.1 ± 18.9<br>Final 50% stance: 28.7 ± 30.0	*p* = 0.001; ↓<br>*p* = 0.037; ↔
Sedighi et al. [65]	Biceps Femoris	First 50% stance: 22.3 ± 16.9<br>Final 50% stance: 35.8 ± 22.0	First 50% stance: 36.0 ± 19.0<br>Final 50% stance: 31.7 ± 17.2	*p* = 0.004; ↓<br>*p* = 0.232; ↔
Sedighi et al. [65]	Tibialis Anterior	First 50% stance: 9.4 ± 7.2<br>Final 50% stance: 9.4 ± 4.2	First 50% stance: 16.0 ± 7.0<br>Final 50% stance: 8.5 ± 3.6	*p* = 0.126; ↔<br>*p* = 0.702; ↔
Sedighi et al. [65]	Gastrocnemius Lateralis	First 50% stance: 52.1 ± 20.3<br>Final 50% stance: 38.8 ± 14.3	First 50% stance: 64.7 ± 29.7<br>Final 50% stance: 52.0 ± 20.8	*p* = 0.920; ↔<br>*p* = 0.001; ↓
		**Co-contraction variables**		

Email corresponding author: basvanhooren@hotmail.com. Department of Nutrition and Movement Sciences, NUTRIM School of Nutrition and Translational Research in Metabolism, Maastricht University Medical Centre+, Maastricht, The Netherlands

Montgomery et al. [61]	Rectus femoris - semitendinosus	2.88 m/s: 42.5 ± 23.5<br>4.28 m/s: 45.1 ± 20.1	2.88 m/s: 37.8 ± 15.3<br>4.28 m/s: 40.1 ± 10.8	$p = 1$; ↔<br>$p = 0.52$; ↔
Montgomery et al. [61]	Tibialis anterior - soleus	2.88 m/s: 188.6 ± 64.5<br>4.28 m/s: 225.1 ± 76.9	2.88 m/s: 191.4 ± 65.4<br>4.28 m/s: 207.2 ± 72.6	$p = 0.99$; ↔<br>$p = 0.83$; ↔
		**Normalized peak EMG**		
Oliveira et al. [50]	Tibialis anterior	Not reported/provided	Not reported/provided	$p = 0.005$; ↓
Oliveira et al. [50]	Peroneus longus	Not reported/provided	Not reported/provided	n.s.; ↔
Oliveira et al. [50]	Soleus	Not reported/provided	Not reported/provided	$p = 0.005$; ↓
Oliveira et al. [50]	Gastrocnemius lateralis	Not reported/provided	Not reported/provided	n.s.; ↔
Oliveira et al. [50]	Gastrocnemius medialis	Not reported/provided	Not reported/provided	n.s.; ↔
Oliveira et al. [50]	Vastus medialis	Not reported/provided	Not reported/provided	n.s.; ↔
Oliveira et al. [50]	Rectus femoris	Not reported/provided	Not reported/provided	n.s.; ↔
Oliveira et al. [50]	Biceps femoris	Not reported/provided	Not reported/provided	n.s.; ↔
Oliveira et al. [50]	Semitendinosus	Not reported/provided	Not reported/provided	n.s.; ↔
Oliveira et al. [50]	Gluteus maximus	Not reported/provided	Not reported/provided	n.s.; ↔
		**Magnitude normalized sEMG ratio**		
Wang et al. [68]	**Rectus femoris**<br>Phase 1 (stance phase)	0.037 ± 0.023	Concrete: 0.247 ± 0.13<br>Synthetic rubber: 0.213 ± 0.076<br>Natural grass: 0.154 ± 0.045	$p < 0.05$; ↓<br>$p < 0.05$; ↓<br>$p < 0.05$; ↓
Wang et al. [68]	Phase 2 (early swing phase)	0.038 ± 0.022	Concrete: 0.091 ± 0.039<br>Synthetic rubber: 0.07 ± 0.021<br>Natural grass: 0.042 ± 0.016	n.s.; ↔<br>n.s.; ↔<br>n.s.; ↔

Email corresponding author: basvanhooren@hotmail.com. Department of Nutrition and Movement Sciences, NUTRIM School of Nutrition and Translational Research in Metabolism, Maastricht University Medical Centre+, Maastricht, The Netherlands

Wang et al. [68]	Phase 3 (middle swing)	0.037 ± 0.019	Concrete: 0.051 ± 0.052<br>Synthetic rubber: 0.037 ± 0.02<br>Natural grass: 0.03 ± 0.021	n.s.; ↔<br>n.s.; ↔<br>n.s.; ↔
Wang et al. [68]	Phase 4 (late swing)	0.024 ± 0.005	Concrete: 0.05 ± 0.009<br>Synthetic rubber: 0.017 ± 0.008<br>Natural grass: 0.03 ± 0.006	n.s.; ↔<br>n.s.; ↔<br>n.s.; ↔
	**Tibialis anterior**			
Wang et al. [68]	Phase 1 (stance phase)	0.083 ± 0.031	Concrete: 0.144 ± 0.06<br>Synthetic rubber: 0.105 ± 0.042<br>Natural grass: 0.114 ± 0.061	n.s.; ↔<br>n.s.; ↔<br>n.s.; ↔
Wang et al. [68]	Phase 2 (early swing phase)	0.066 ± 0.011	Concrete: 0.14 ± 0.004<br>Synthetic rubber: 0.079 ± 0.024<br>Natural grass: 0.093 ± 0.006	n.s.; ↔<br>n.s.; ↔<br>n.s.; ↔
Wang et al. [68]	Phase 3 (middle swing)	0.092 ± 0.022	Concrete: 0.129 ± 0.022<br>Synthetic rubber: 0.102 ± 0.035<br>Natural grass: 0.1 ± 0.032	n.s.; ↔<br>n.s.; ↔<br>n.s.; ↔
Wang et al. [68]	Phase 4 (late swing)	0.122 ± 0.095	Concrete: 0.139 ± 0.105<br>Synthetic rubber: 0.113 ± 0.042<br>Natural grass: 0.164 ± 0.14	n.s.; ↔<br>n.s.; ↔<br>n.s.; ↔
	**Biceps femoris**			
Wang et al. [68]	Phase 1 (stance phase)	0.048 ± 0.028	Concrete: 0.128 ± 0.126<br>Synthetic rubber: 0.133 ± 0.072<br>Natural grass: 0.099 ± 0.062	$p < 0.05$; ↓<br>$p < 0.05$; ↓<br>$p < 0.05$; ↓
Wang et al. [68]	Phase 2 (early swing phase)	0.024 ± 0.012	Concrete: 0.083 ± 0.025<br>Synthetic rubber: 0.045 ± 0.011<br>Natural grass: 0.057 ± 0.011	n.s.; ↔<br>n.s.; ↔<br>n.s.; ↔
Wang et al. [68]	Phase 3 (middle swing)	0.064 ± 0.053	Concrete: 0.133 ± 0.086<br>Synthetic rubber: 0.098 ± 0.07<br>Natural grass: 0.102 ± 0.07	n.s.; ↔<br>n.s.; ↔<br>n.s.; ↔
Wang et al. [68]	Phase 4 (late swing)	0.124 ± 0.107	Concrete: 0.128 ± 0.08<br>Synthetic rubber: 0.16 ± 0.138<br>Natural grass: 0.102 ± 0.068	n.s.; ↔<br>n.s.; ↔<br>n.s.; ↔
	**Gastrocnemius**			

Email corresponding author: basvanhooren@hotmail.com. Department of Nutrition and Movement Sciences, NUTRIM School of Nutrition and Translational Research in Metabolism, Maastricht University Medical Centre+, Maastricht, The Netherlands

Wang et al. [68]	Phase 1 (stance phase)	0.474 ± 0.311	Concrete: 0.6 ± 0.405<br>Synthetic rubber: 0.622 ± 0.23<br>Natural grass: 0.609 ± 0.399	n.s.; ↔<br>n.s.; ↔<br>n.s.; ↔
Wang et al. [68]	Phase 2 (early swing phase)	0.144 ± 0.031	Concrete: 0.179 ± 0.045<br>Synthetic rubber: 0.236 ± 0.057<br>Natural grass: 0.174 ± 0.073	n.s.; ↔<br>n.s.; ↔<br>n.s.; ↔
Wang et al. [68]	Phase 3 (middle swing)	0.07 ± 0.043	Concrete: 0.074 ± 0.054<br>Synthetic rubber: 0.09 ± 0.078<br>Natural grass: 0.052 ± 0.05	n.s.; ↔<br>n.s.; ↔<br>n.s.; ↔
Wang et al. [68]	Phase 4 (late swing)	0.066 ± 0.018	Concrete: 0.179 ± 0.008<br>Synthetic rubber: 0.078 ± 0.004<br>Natural grass: 0.128 ± 0.007	n.s.; ↔<br>n.s.; ↔<br>n.s.; ↔
		**Integrated EMG**		
Oliveira et al. [50]	Tibialis anterior	Not reported/provided	Not reported/provided	$p = 0.005$; ↓
Oliveira et al. [50]	Peroneus longus	Not reported/provided	Not reported/provided	$p = 0.005$; ↓
Oliveira et al. [50]	Soleus	Not reported/provided	Not reported/provided	$p = 0.005$; ↓
Oliveira et al. [50]	Gastrocnemius lateralis	Not reported/provided	Not reported/provided	n.s.; ↔
Oliveira et al. [50]	Gastrocnemius medialis	Not reported/provided	Not reported/provided	n.s.; ↔
Oliveira et al. [50]	Vastus medialis	Not reported/provided	Not reported/provided	n.s.; ↔
Oliveira et al. [50]	Rectus femoris	Not reported/provided	Not reported/provided	n.s.; ↔
Oliveira et al. [50]	Biceps femoris	Not reported/provided	Not reported/provided	n.s.; ↔
Oliveira et al. [50]	Semitendinosus	Not reported/provided	Not reported/provided	n.s.; ↔
Oliveira et al. [50]	Gluteus maximus	Not reported/provided	Not reported/provided	n.s.; ↔
		**Mean integrated EMG values (mV/s)***		

Email corresponding author: basvanhooren@hotmail.com. Department of Nutrition and Movement Sciences, NUTRIM School of Nutrition and Translational Research in Metabolism, Maastricht University Medical Centre+, Maastricht, The Netherlands

Wank et al. [69]	Gluteus maximus	Pre-contact 4 m/s: 0.029 ± -<br>Pre-contact 6 m/s: 0.058 ± -<br>Ground contact 4 m/s: 0.035 ± -<br>Ground contact 6 m/s: 0.036 ± -<br>Swing 4 m/s: 0.017 ± -<br>Swing 6 m/s: 0.027 ± -	Pre-contact 4 m/s: 0.027 ± -<br>Pre-contact 6 m/s: 0.054 ± -<br>Ground contact 4 m/s: 0.036 ± -<br>Ground contact 6 m/s: 0.041 ± -<br>Swing 4 m/s: 0.017 ± -<br>Swing 6 m/s: 0.031 ± -	-<br>-<br>-<br>-<br>-<br>-
Wank et al. [69]	Biceps femoris long head	Pre-contact 4 m/s: 0.068 ± -<br>Pre-contact 6 m/s: 0.115 ± -<br>Ground contact 4 m/s: 0.087 ± -<br>Ground contact 6 m/s: 0.082 ± -<br>Swing 4 m/s: 0.024 ± -<br>Swing 6 m/s: 0.035 ± -	Pre-contact 4 m/s: 0.063 ± -<br>Pre-contact 6 m/s: 0.113 ± -<br>Ground contact 4 m/s: 0.068 ± -<br>Ground contact 6 m/s: 0.080 ± -<br>Swing 4 m/s: 0.017 ± -<br>Swing 6 m/s: 0.031 ± -	-<br>-<br>-<br>-<br>-<br>-
Wank et al. [69]	Rectus femoris	Pre-contact 4 m/s: 0.015 ± -<br>Pre-contact 6 m/s: 0.021 ± -<br>Ground contact 4 m/s: 0.024 ± -<br>Ground contact 6 m/s: 0.023 ± -<br>Swing 4 m/s: 0.030 ± -<br>Swing 6 m/s: 0.062 ± -	Pre-contact 4 m/s: 0.014 ± -<br>Pre-contact 6 m/s: 0.022 ± -<br>Ground contact 4 m/s: 0.015 ± -<br>Ground contact 6 m/s: 0.026 ± -<br>Swing 4 m/s: 0.024 ± -<br>Swing 6 m/s: 0.050 ± -	-<br>-<br>-<br>-<br>-<br>-
Wank et al. [69]	Vastus lateralis	Pre-contact 4 m/s: 0.035 ± -<br>Pre-contact 6 m/s: 0.055 ± -<br>Ground contact 4 m/s: 0.077 ± -<br>Ground contact 6 m/s: 0.070 ± -<br>Swing 4 m/s: 0.014 ± -<br>Swing 6 m/s: 0.025 ± -	Pre-contact 4 m/s: 0.038 ± -<br>Pre-contact 6 m/s: 0.065 ± -<br>Ground contact 4 m/s: 0.081 ± -<br>Ground contact 6 m/s: 0.078 ± -<br>Swing 4 m/s: 0.014 ± -<br>Swing 6 m/s: 0.025 ± -	-<br>-<br>-<br>-<br>-<br>-
Wank et al. [69]	Gastrocnemius lateralis	Pre-contact 4 m/s: 0.045 ± -<br>Pre-contact 6 m/s: 0.082 ± -<br>Ground contact 4 m/s: 0.111 ± -<br>Ground contact 6 m/s: 0.097 ± -<br>Swing 4 m/s: 0.021 ± -<br>Swing 6 m/s: 0.031 ± -	Pre-contact 4 m/s: 0.038 ± -<br>Pre-contact 6 m/s: 0.073 ± -<br>Ground contact 4 m/s: 0.110 ± -<br>Ground contact 6 m/s: 0.099 ± -<br>Swing 4 m/s: 0.017 ± -<br>Swing 6 m/s: 0.033 ± -	-<br>-<br>-<br>-<br>-<br>-
Wank et al. [69]	Soleus	Pre-contact 4 m/s: 0.090 ± -<br>Pre-contact 6 m/s: 0.083 ± -<br>Ground contact 4 m/s: 0.034 ± -<br>Ground contact 6 m/s: 0.044 ± -<br>Swing 4 m/s: 0.016 ± -	Pre-contact 4 m/s: 0.094 ± -<br>Pre-contact 6 m/s: 0.080 ± -<br>Ground contact 4 m/s: 0.039 ± -<br>Ground contact 6 m/s: 0.044 ± -<br>Swing 4 m/s: 0.017 ± -	-<br>-<br>-<br>-<br>-

Email corresponding author: basvanhooren@hotmail.com. Department of Nutrition and Movement Sciences, NUTRIM School of Nutrition and Translational Research in Metabolism, Maastricht University Medical Centre+, Maastricht, The Netherlands

		Swing 6 m/s: 0.026 ± -	Swing 6 m/s: 0.023 ± -	-
		**Mean EMG values (μV) over whole gait cycle**		
Roussos et al. [64]	Biceps femoris	66.15 ± 6.26	78.49 ± 8.41	$p < 0.01$; ↓
Roussos et al. [64]	Gastrocnemius	75.36 ± 6.93	92.84 ± 7.31	$p < 0.01$; ↓
Roussos et al. [64]	Rectus remoris	48.79 ± 6.59	62.56 ± 9.74	$p < 0.01$; ↓
Roussos et al. [64]	Vastus medialis	39.45 ± 6.65	49.95 ± 6.08	$p < 0.01$; ↓
Roussos et al. [64]	Average over all muscles	57.44 ± 1.1	70.96 ± 1.3	-
		**Inter-subject similarity for motor modules (a.u.)***		
Oliveira et al. [50]	Motor module M1	0.87 ± 0.06	0.90 ± 0.03	n.s.; ↔
Oliveira et al. [50]	Motor module M2	0.92 ± 0.03	0.90 ± 0.03	n.s.; ↔
Oliveira et al. [50]	Motor module M3	0.68 ± 0.13	0.71 ± 0.11	n.s.; ↔
Oliveira et al. [50]	Motor module M4	0.89 ± 0.05	0.87 ± 0.06	$p < 0.001$; ↑
		**Peak timing of motor modules (% gait cycle)***		
Oliveira et al. [50]	Peak timing of activation signals M1	5.0 ± 2.4	5.0 ± 2.1	n.s.; ↔
Oliveira et al. [50]	Peak timing of activation signals M2	14.8 ± 3.7	15.3 ± 3.7	n.s.; ↔
Oliveira et al. [50]	Peak timing of activation signals M3	54.8 ± 8.7	54.8 ± 9.0	n.s.; ↔

Email corresponding author: basvanhooren@hotmail.com. Department of Nutrition and Movement Sciences, NUTRIM School of Nutrition and Translational Research in Metabolism, Maastricht University Medical Centre+, Maastricht, The Netherlands

Oliveira et al. [50]	Peak timing of activation signals M4	84.7 ± 2.6	84.9 ± 4.5	n.s.; ↔
		**Magnitude of peak of motor modules (a.u.)***		
Oliveira et al. [50]	Magnitude of peak M1	3.2 ± 0.6	3.5 ± 0.5	$p < 0.01$; ↓
Oliveira et al. [50]	Magnitude of peak M2	3.1 ± 0.9	2.8 ± 0.6	n.s.; ↔
Oliveira et al. [50]	Magnitude of peak M3	1.5 ± 0.4	1.2 ± 0.4	$p < 0.01$; ↑
Oliveira et al. [50]	Magnitude of peak M4	2.8 ± 0.6	2.4 ± 0.4	$p < 0.01$; ↑

*Data extracted using WebPlotDigitizer; †SD computed from 95% confidence interval and t-distribution as described by Higgins, Deeks [102].

Email corresponding author: basvanhooren@hotmail.com. Department of Nutrition and Movement Sciences, NUTRIM School of Nutrition and Translational Research in Metabolism, Maastricht University Medical Centre+, Maastricht, The Netherlands

Table SVII. Muscle-tendon unit and bone outcome measures

Study	Outcome Measure	Treadmill mean ± SD	Overground mean ± SD	P-value (if available); arrow indicating direction of effect compared to overground
		**Fascicle length change (mm)***		
Cronin, Finni [54]	Gastrocnemius medialis	9.6 ± 1.7	9.3 ± 1.2	n.s.; ↔
Cronin, Finni [54]	Soleus	7.1 ± 1.8	6.8 ± 1.4	n.s.; ↔
		**Fascicle velocity (mm/s)***		
Cronin, Finni [54]	Gastrocnemius medialis	-39.4 ± 10.6	-39.4 ± 9.5	n.s.; ↔
Cronin, Finni [54]	Soleus	-11.4 ± 4.7	-13.0 ± 5.9	n.s.; ↔
		**Plantar fascia strain (%)**		
Sinclair et al. [67]	Plantar fascia	5.53 ± 2.25	8.23 ± 2.77	$p < .05$; ↓
		**Bone strain variables**		
Milgrom et al. [6]	Peak tibial axial compression strain (με)	664 ± 274.3	1957 ± 505.1	$p < .001$; ↓
Milgrom et al. [6]	Peak tibial axial tension strain (με)	860 ± 332.8	1273 ± 93.9	$p < .001$; ↓
Milgrom et al. [6]	Peak tibial axial compression strain rate (με/s)	3346 ± 777.6	12876 ± 3178.9	$p < .001$; ↓
Milgrom et al. [6]	Peak tibial axial tension strain rate (με/s)	6645 ± 3435.6	14160 ± 4287.1	$p < .001$; ↓

*Data extracted using WebPlotDigitizer.

Email corresponding author: basvanhooren@hotmail.com. Department of Nutrition and Movement Sciences, NUTRIM School of Nutrition and Translational Research in Metabolism, Maastricht University Medical Centre+, Maastricht, The Netherlands

References

See the manuscript for all references. Two references used here are not cited in the manuscript:

101. Hozo SP, Djulbegovic B, Hozo I. Estimating the mean and variance from the median, range, and the size of a sample. BMC Med Res Methodol. 2005;5(1):13. doi:10.1186/1471-2288-5-13.
102. Higgins JP, Deeks JJ. Chapter 7: Selecting studies and collecting data. In: Higgins JP, Green S, editors. Cochrane Handbook for Systematic Reviews of Interventions. Version 5.1.0 ed.: The Cochrane Collaboration; 2011.
