# Supplementary material for: Is Motorized Treadmill Running Biomechanically Comparable to Overground Running? A Systematic Review and Meta-Analysis of Cross-Over Studies
Source: Sports Med. 2019 Dec 4;50(4):785–813. doi: 10.1007/s40279-019-01237-z (PMC7069922; doi:10.1007/s40279-019-01237-z)
Supplement: Supplementary file 4 — Supplementary material 4 (PDF 410 kb) [file 40279_2019_1237_MOESM4_ESM.pdf]

Email corresponding author: basvanhooren@hotmail.com. Department of Nutrition and Movement Sciences, NUTRIM School of Nutrition and Translational Research in Metabolism, Maastricht University Medical Centre+, Maastricht, The Netherlands

Appendix file IV Extended meta-analysis and meta-regression results

1.1.1 Contact time

Pooled results from six studies indicated that treadmill contact time did not significantly differ from track contact time (mean difference 4.34 ms; 95% CI: -3.44 to 12.11; *n* = 79; *k* = 10; I^2 = 86.7%). Pooled results from three studies showed that treadmill contact time did not significantly differ from concrete contact time (mean difference 6.72 ms; -7.25 to 20.70; *n* = 25; *k* = 3; I^2 = 72.9%). Pooled results from six studies showed that treadmill contact time did not significantly differ from lab runway contact time (mean difference 5.78 ms; -2.73 to 14.29; *n* = 90; *k* = 10; I^2 = 82.2%). Pooled results from two studies indicated that treadmill contact time did not significantly differ from grass contact time (mean difference 2.41 ms; -7.61 to 12.43; *n* = 12; *k* = 2; I^2 = 47.6%). When the effects were combined across all overground surfaces, treadmill running however resulted in a significant longer ground contact time by 5 ms (95% CI: 0.48 to 9.51; *n* = 106; *k* = 25; I^2 = 81%). Treadmill running speed, motor power, belt length and belt width were no statistically significant moderator of the mean difference in ground contact time (β= 0.01 ms; -1.39 to 1.40; β= 1.22 ms; -0.58 to 3.03; β= 5.43 ms; -6.0 to 16.9; β= 17.38 ms; -12.6 to 47.3, respectively).

1.1.2 Stride time

Pooled results from two studies indicated that treadmill stride time did not significantly differ from track running stride time (mean difference -19.4 ms; -46.6 to 7.8; *n* = 38; *k* = 5; I^2 = 89.3%). Pooled results from five studies indicated that treadmill stride time was also not significantly different from lab runway stride time (mean difference -7.4 ms; -22.6 to 7.8; *n* = 71; *k* = 8; I^2 = 97.2%). When pooled across all overground surfaces, treadmill running stride time was not significantly different from overground (mean difference -12.0; -25.8 to 1.8; *n* = 109; *k* = 13; I^2 = 97.1%). Treadmill running speed was no statistically significant moderator of the mean difference in stride time (β= -0.23 ms; -4.05 to 4.52). Treadmill motor power, belt length and belt width were statistically significant moderators of the mean difference in stride time (β= 2.88 ms; -5.69 to -0.06, β= -22.0; -30.7 to -13.3; β= -70.2 ms; -124.13 to 16.2, respectively).

1.1.3 Stride length

Pooled results from three studies indicated that treadmill stride length was not significantly different from track stride length (mean difference -9.6 cm; -20.6 to 1.5; *n* = 61; *k* = 8; I^2 = 97.0%). Pooled results from five other studies showed that treadmill stride length was not significantly different from lab runway stride length (mean difference -1.4 cm; -8.2 to 5.4; *n* = 84; *k* = 7; I^2 = 59.0%). Pooled results across all surfaces showed no significant difference in stride length (mean difference –5.0 cm; -11.5 to 1.6; *n* = 145; *k* = 15; I^2 = 92.8%). Treadmill running speed, motor power, belt length and belt width were no statistically significant moderator of the mean difference in stride length (β= -0.49 cm; -2.2 to 1.21; β= -0.65 cm; -2.81 to 1.51; β= -8.29 cm; -21.0 to 4.43; β= -8.96 cm; -47.7 to 29.7, respectively).

1.1.4 Stride frequency

Pooled results from three studies indicated that treadmill stride frequency was not significantly different from track stride frequency (mean difference 0.05 strides/s; -0.02 to 0.12; *n* = 61; *k* = 8; I^2 = 97.1%). Pooled results from another study indicated that treadmill stride frequency was not significantly different from concrete stride frequency (mean difference 0.04 strides/s; -0.07 to 0.14; *n* = 4; *k* = 2; I^2 = 0%). Pooled results from four studies indicated that treadmill stride frequency was not significantly different from lab runway stride frequency (mean difference -0.02 strides/s; -0.07 to 0.03; *n* = 68; *k* = 6; I^2 = 71.5%). Pooled results across all surfaces showed no significant difference between treadmill and overground stride frequency (mean difference 0.02 strides/s; -0.03 to 0.06; *n* = 133; *k* = 16; I^2 = 92.2%). Treadmill running speed, motor power, belt length and belt width were no statistically significant moderator of the mean difference in stride length (β= 0.004 strides/s; -0.006 to 0.01; β= -0.002 strides/s; -0.04 to 0.04; β= 0.07 strides/s; -0.04 to 0.17; β= 0.08 strides/s; -0.2 to 0.4, respectively).

1.2 Ankle and foot kinematic outcome measures

1.2.1 Ankle angle at footstrike

Email corresponding author: basvanhooren@hotmail.com. Department of Nutrition and Movement Sciences, NUTRIM School of Nutrition and Translational Research in Metabolism, Maastricht University Medical Centre+, Maastricht, The Netherlands

Results from one study indicated that treadmill ankle angle at footstrike was significantly lower (i.e., less dorsiflexed relative to neutral) compared to concrete by -6.1 degrees (-9.6 to -2.6; n = 12; k = 1). Pooled results from three studies indicated that treadmill ankle angle at footstrike was not significantly different from lab runway ankle angle (mean difference -1.9 degrees; -7.0 to 3.3; n = 43; k = 4; I^2 = 74.4%). When pooled across all surfaces, treadmill ankle angle at footstrike did not significantly differ from overground (mean difference -2.3 degrees; -7.2 to 1.4; n = 55; k = 5; I^2 = 79.1%).

1.2.2 Foot angle at footstrike

Pooled results from one study indicated that treadmill foot angle at footstrike was significantly lower (i.e., less inclined) than track foot angle by -7.8 degrees (-14.4 to -1.2; n = 10; k = 2; I^2 = 0%). Results from one study indicated that treadmill foot angle at footstrike was significantly lower (i.e., less inclined) compared to concrete by -10.5 degrees (-14.3 to -6.7; n = 12; k = 1). When pooled across all surfaces, treadmill foot angle at footstrike was significantly lower (less inclined) compared to overground foot angle by -9.8 degrees (-13.1 to -6.6; n = 22; k = 3; I^2 = 0%).

1.2.3 Peak ankle angle during stance

Pooled results from four studies showed that treadmill peak ankle angle during stance was not significantly different from lab runway peak ankle angle (mean difference -0.6 degrees; -1.3 to 0.2; n = 70; k = 4; I^2 = 0%).

1.2.4 Ankle dorsiflexion range of motion from footstrike to peak dorsiflexion

Results from one study indicated that treadmill ankle dorsiflexion range of motion was significantly higher (i.e., more dorsiflexion range of motion) when compared to concrete ankle dorsiflexion range of motion by 5.3 degrees (2.5 to 8.1; n = 12; k = 1). Results from one other study however indicated that treadmill ankle dorsiflexion range of motion was significantly lower when compared to a lab runway by -6.5 degrees (-10.4 to -2.6; n = 12; k = 1). Pooled results across all surfaces showed no significant difference between treadmill and overground ankle dorsiflexion range of motion (mean difference -0.52 degrees; -12.1 to 11.0; n = 24; k = 2; I^2 = 95.8%).

1.2.5 Ankle in- and eversion at footstrike

Pooled results from two studies showed that treadmill ankle in- and eversion at footstrike was not significantly different from lab runway ankle in- and eversion (mean difference -3.3 degrees; -8.4 to 1.8; n = 32; k = 2; I^2 = 74.4%).

1.2.6 Peak ankle in- and eversion during stance

Pooled results from two studies showed that treadmill peak ankle in- and eversion during stance was not significantly different from lab runway peak ankle in- and eversion (mean difference -2.5 degrees; -9.1 to 4.0; n = 32; k = 2; I^2 = 85.7%).

1.2.7 Ankle add- and abduction at footstrike

Pooled results from two studies showed that treadmill ankle add- and abduction at footstrike was not significantly different from lab runway ankle add- and abduction (mean difference 1.0 degrees; -4.5 to 6.5; n = 32; k = 2; I^2 = 89.8%).

1.2.8 Peak ankle add- and abduction during stance

Pooled results from two studies showed that treadmill peak ankle add- and abduction during stance was not significantly different from lab runway peak ankle add- and abduction (mean difference 0.45 degrees; -1.7 to 2.6; n = 32; k = 2; I^2 = 54.5%).

1.3 Knee kinematic outcome measures

1.3.1 Knee flexion at footstrike

Pooled results from one study show that treadmill knee flexion at footstrike was significantly higher than track knee flexion by -2.7 degrees (-4.9 to -0.5; n = 10; k = 2; I^2 = 0%). Results from one study show that treadmill knee flexion at footstrike was significantly higher than concrete knee flexion by -2.8 degrees (-5.1 to -0.5; n = 12; k = 1). Pooled results from three studies show that treadmill knee flexion at footstrike was not significantly different from lab runway

Email corresponding author: basvanhooren@hotmail.com. Department of Nutrition and Movement Sciences, NUTRIM School of Nutrition and Translational Research in Metabolism, Maastricht University Medical Centre+, Maastricht, The Netherlands

knee flexion (mean difference -1.7 degrees; -3.7 to 0.4; n = 43; k = 4; I^2 = 0%). When pooled across all surfaces, treadmill knee flexion at footstrike was significantly higher (i.e., more flexed) than overground knee flexion by -2.3 degrees (-3.6 to -1.1; n = 65; k = 7; I^2 = 0%). As heterogeneity was very low, meta-regression was not performed.

1.3.2 Peak knee flexion during swing

Pooled results from one study show that treadmill peak knee flexion during swing was not significantly different from track peak knee flexion (mean difference -2.2 degrees; -6.9 to 2.6; n = 10; k = 2; I^2 = 0%). Pooled results from two studies show that treadmill peak knee flexion during swing was not significantly different from lab runway knee flexion (mean difference 3.4 degrees; -0.8 to 7.5; n = 40; k = 2; I^2 = 16.4%). When pooled across all surfaces, treadmill peak knee flexion during swing was not significantly different from overground peak knee flexion (mean difference 1.2 degrees; -2.2 to 4.5; n = 50; k = 4; I^2 = 21.7%).

1.3.3 Peak knee flexion angle during stance

Pooled results from one study show that treadmill peak knee flexion during stance was not significantly different from track peak knee flexion (mean difference -1.7 degrees; -3.6 to 0.1; n = 10; k = 2; I^2 = 0%). Pooled results from three studies show that treadmill peak knee flexion during stance was not significantly different from lab runway peak knee flexion (mean difference 1.9 degrees; -0.8 to 4.5; n = 50; k = 3; I^2 = 79.3%). When pooled across all surfaces, treadmill peak knee flexion during stance was not significantly different from overground peak knee flexion (mean difference 0.5 degrees; -1.8 to 2.8; n = 60; k = 5; I^2 = 80.1%).

1.3.4 Minimum knee flexion angle during entire gait cycle

Pooled results from two studies show that treadmill minimum knee flexion during the entire gait cycle was not significantly different from lab runway minimum knee flexion (mean difference -0.8 degrees; -2.7 to 1.2; n = 40; k = 2; I^2 = 30%).

1.3.5 Knee flexion range of motion from footstrike to peak during stance

Results from one study show that treadmill knee flexion range of motion from footstrike to peak during stance was significantly lower compared to concrete knee flexion range of motion by 6.4 degrees (4.3 to 8.5; n = 12; k = 1). Results from one other study also indicate that treadmill knee flexion range of motion from footstrike to peak during stance was significantly lower compared to a lab runway knee flexion range of motion by 6.1 degrees (2.2 to 10.0 ; n = 12; k = 1). When pooled across all surfaces, treadmill knee flexion range of motion from footstrike to peak during stance was significantly lower (i.e., smaller range of motion) compared to overground by 6.3 degrees (4.5 to 8.2; n = 24; k = 2; I^2 = 0%).

1.3.6 Knee add- and abduction at footstrike

Pooled results from two studies show that treadmill knee add- and abduction angle at footstrike was not significantly different from lab runway knee add- and abduction (mean difference 0.6 degrees; -1.7 to 2.9; n = 32; k = 2; I^2 = 56.6%).

1.3.7 Knee angle at toe-off

Pooled results from one study indicated that treadmill knee angle at toe-off was not significantly different from track (mean difference -0.7 degrees; -2.6 to 1.2; n = 10; k = 2; I^2 = 0%). Pooled results from one other study showed that treadmill knee angle at toe-off was not significantly different from lab runway (mean difference -0.6 degrees; -6.2 to 5.0; n = 11; k = 2; I^2 = 0%). When pooled across all surfaces, treadmill knee angle at toe-off was not significantly different from overground (mean difference -0.7 degrees; -2.5 to 1.1; n = 21; k = 4; I^2 = 0%). As heterogeneity was very low, meta-regression was not performed.

1.3.8 Peak knee add- and abduction during stance

Pooled results from two studies show that treadmill peak knee add- and abduction angle during stance was not significantly different from lab runway knee add- and abduction (mean difference 0.3 degrees; -3.2 to 3.8; n = 32; k = 2; I^2 = 55.4%).

1.3.9 Knee in- and external rotation at footstrike

Email corresponding author: basvanhooren@hotmail.com. Department of Nutrition and Movement Sciences, NUTRIM School of Nutrition and Translational Research in Metabolism, Maastricht University Medical Centre+, Maastricht, The Netherlands

Pooled results from two studies show that treadmill knee in- and external rotation at footstrike was not significantly different from lab runway knee in- and external rotation (mean difference 1.0 degrees; -1.7 to 3.6; n = 32; k = 2; I^2 = 0%).

1.3.10 Peak knee in- and external rotation angle during stance

Pooled results from two studies show that treadmill peak knee in- and external rotation during stance was not significantly different from lab runway knee in- and external rotation (mean difference -0.9 degrees; -3.1 to 1.2; n = 32; k = 2; I^2 = 0%).

1.4 Hip and pelvic kinematic outcome measures

1.4.1 Hip flexion at footstrike

Results from one study show that treadmill hip flexion at footstrike was significantly lower compared to track by -4.1 degrees (-6.4 to -1.8; n = 10; k = 1). Pooled results from three studies show that treadmill hip flexion at footstrike was not significantly different from lab runway hip flexion (mean difference -2.5 degrees; -7.4 to 2.4; n = 43; k = 4; I^2 = 77.3%). When pooled across all surfaces, treadmill hip flexion at footstrike was not significantly different from overground hip flexion (mean difference -2.7 degrees; -6.2 to 0.8; n = 53; k = 5; I^2 = 75.3%).

1.4.2 Peak hip flexion during stance

Results from one study show that treadmill peak hip flexion during stance was significantly lower compared to track by -4.2 degrees (-6.3 to -2.1; n = 10; k = 1). Pooled results from two studies show that treadmill peak hip flexion during stance was not significantly different from lab runway hip flexion (mean difference -6.5 degrees; -18.3 to 5.2; n = 32; k = 2; I^2 = 95.3%). When pooled across all surfaces, treadmill peak hip flexion during stance was not significantly different from overground hip flexion (mean difference -5.6 degrees; -12.3 to 1.1; n = 42; k = 3; I^2 = 95.1%).

1.4.3 Peak hip flexion angle during entire gait cycle

Pooled results from two studies indicate that treadmill peak hip flexion angle during the entire gait cycle was not significantly different from track (mean difference -3.2 degrees; -7.1 to 0.8; n = 20; k = 3; I^2 = 55.8%). Results from one study indicate that treadmill peak hip flexion angle during the entire gait cycle is not significantly different from lab runway (mean difference -1.3 degrees; -4.3 to 1.7; n = 20; k = 1). When pooled across all surfaces, treadmill peak hip flexion angle during the entire gait cycle was not significantly different from overground (mean difference -2.2 degrees; -4.6 to 0.2; n = 40; k = 4; I^2 = 33.8%).

1.4.4 Hip range of motion during stance

Pooled results from two studies show that treadmill hip range of motion during stance was not significantly different from lab runway hip range of motion (mean difference -9.0 degrees; -24.2 to 6.1; n = 32; k = 2; I^2 = 95.2%).

1.4.5 Peak hip extension during gait cycle

Pooled results from two studies indicate that treadmill peak hip extension during the entire gait cycle was not significantly different from track hip extension (mean difference 3.6 degrees; -2.7 to 10.0; n = 20; k = 3; I^2 = 92.4%). Results from one study indicate that treadmill peak hip extension during the entire gait cycle is not significantly different from lab runway hip extension (mean difference 0.3 degrees; -2.2 to 2.8; n = 20; k = 1). When pooled across all surfaces, treadmill peak hip extension during the entire gait cycle was not significantly different from overground hip extension (mean difference 2.8 degrees; -2.0 to 7.5; n = 40; k = 4; I^2 = 90.9%).

1.4.6 Hip angle at toe-off

Results from one study indicated that treadmill hip angle at toe-off was significantly higher than track by -6.1 degrees (-9.4 to -2.8; n = 10; k = 1). Pooled results from one other study showed that treadmill hip angle at toe-off was not significantly different from lab runway (mean difference 1.2 degrees; -2.1 to 4.5; n = 11; k = 2; I^2 = 0%). When pooled across all surfaces, treadmill knee angle at toe-off was not significantly different from overground (mean difference -1.5 degrees; -6.5 to 3.6; n = 21; k = 3; I^2 = 77.0%).

Email corresponding author: basvanhooren@hotmail.com. Department of Nutrition and Movement Sciences, NUTRIM School of Nutrition and Translational Research in Metabolism, Maastricht University Medical Centre+, Maastricht, The Netherlands

1.4.7 Hip add- and abduction at footstrike

Results from one study show that treadmill hip add- and abduction at footstrike did not significantly differ from track hip add- and abduction (mean difference 0.5 degrees; -0.7 to 1.7; n = 10; k = 1). Pooled results from two studies show that treadmill hip add- and abduction at footstrike was not significantly different from lab runway hip add- and abduction (mean difference 0.75 degrees; -0.7 to 2.2; n = 32; k = 2; I^2 = 0%). When pooled across all surfaces, treadmill hip add- and abduction at footstrike was not significantly different from overground (mean difference 0.6 degrees; -0.4 to 1.6; n = 42; k = 3; I^2 = 0%).

1.4.8 Peak hip add- and abduction during stance

Results from one study show that treadmill peak hip add- and abduction during stance did not significantly differ from track hip add- and abduction (mean difference 0.7 degrees; -1.4 to 2.8; n = 10; k = 1). Pooled results from three studies show that treadmill peak hip add- and abduction during stance was not significantly different from lab runway hip add- and abduction (mean difference 0.6 degrees; -0.5 to 1.8; n = 52; k = 3; I^2 = 0%). When pooled across all surfaces, treadmill peak hip add- and abduction during stance was not significantly different from overground hip add- and abduction (mean difference 0.6 degrees; -0.4 to 1.7; n = 62; k = 4; I^2 = 0%).

1.4.9 Vertical displacement

Pooled results from one study indicate that treadmill vertical displacement is significantly lower than track vertical displacement by -1.1 cm (-2.0 to -0.1; n = 10; k = 2; I^2 = 0%). Pooled results from one other study indicate that treadmill vertical displacement was not significantly different from lab runway vertical displacement (-1.84 cm; -3.99 to 0.03; n = 25; k = 3; I^2 = 88.6%). When pooled across all surfaces, treadmill vertical displacement was significantly lower than overground vertical displacement by -1.47 cm (-2.72 to -0.23; n = 35 k = 5; I^2 = 83.8%).

1.5 Kinetic outcome measures

1.5.1 Total foot peak pressure

Pooled results from two studies showed that treadmill total foot peak pressure was significantly lower compared to track peak pressure by -1.25 (Hedge's g); -2.13 to -037; n = 18; k = 2; I^2 = 50.6%). Results from one study show that treadmill total foot peak pressure did not significantly differ from concrete peak pressure (-0.4; -1.02 to 0.22; n = 5; k = 1). Pooled results from one study indicate that treadmill total foot peak pressure did not significantly differ from lab runway peak pressure (0.30; -0.06 to 0.66; n = 11; k = 3; I^2 = 0%). Results from one study show that treadmill total foot peak pressure did not significantly differ from grass peak pressure (-0.80; -1.87 to 0.27; n = 4; k = 1). When pooled across all surfaces, treadmill total foot peak pressure did not significantly differ from overground (-0.34; -0.89 to 0.21; n = 38; k = 7; I^2 = 75.3%).

1.5.2 Peak vertical ground reaction force

Pooled results from three studies showed that treadmill peak vertical ground reaction force was not significantly different from lab runway peak vertical ground reaction force (mean difference -0.05 BWs; -0.11 to 0.01; n = 55; k = 6); I^2 = 0%). Treadmill running speed, motor power and belt length and width were no statistically significant moderators of the mean difference in peak vertical ground reaction force (β= -0.10; -0.05 to 0.03; β= -0.04; -0.1 to 0.02; β= -0.09; -0.25 to 0.06; β= -0.24; -1.31 to 0.83, respectively).

1.5.3 Average vertical loading rate

Results from one study show that treadmill average vertical loading rate is significantly lower compared to concrete by -50 BW/s (-64.5 to -35.5; n = 12; k = 1). Pooled results from two studies indicated that average vertical loading rate did not significantly differ from lab runway (mean difference 0.56 BW/s; -4.7 to 5.8; n = 35; k = 5; I^2 = 0%). When pooled across all surfaces, treadmill average vertical loading rate did not significantly differ from overground (mean difference -7.7 BW/s; -24.0 to 8.6; n = 47; k = 6; I^2 = 90.2%).

1.5.4 Instantaneous vertical loading rate

Email corresponding author: basvanhooren@hotmail.com. Department of Nutrition and Movement Sciences, NUTRIM School of Nutrition and Translational Research in Metabolism, Maastricht University Medical Centre+, Maastricht, The Netherlands

Pooled results from two studies indicated that instantaneous vertical loading rate did not significantly differ from lab runway (mean difference 5.8 BW/s; -1.1 to 12.7; *n* = 35; *k* = 5; I^2 = 0%).

1.5.5 Transient peak

Results from one study indicated that treadmill transient peak was significantly lower than concrete transient peak by -0.17 BW/s (-0.28 to -0.05; *n* = 12; *k* = 1; I^2 = 0). Pooled results from one study indicate that transient peak did not significantly differ from lab runway (mean difference 0.05 BW/s; -0.06 to 0.17; *n* = 24; *k* = 3; I^2 = 0%). When pooled across all surfaces, treadmill transient peak did not significantly differ from overground transient peak (mean difference -0.02 BW/s; -0.16 to 0.12; *n* = 36; *k* = 4; I^2 = 58.4%).

1.5.6 Peak propulsive force

Pooled results from two studies indicated that treadmill peak propulsive force was significantly lower than lab runway by -0.04 BW (-0.06 to -0.02; *n* = 31; *k* = 3; I^2 = 0%). As heterogeneity was very low, meta-regression was not performed.

1.5.7 Ankle sagittal plane joint moment

Pooled results from two studies indicated that treadmill ankle sagittal plane joint moment was significantly higher than lab runway ankle sagittal plane joint moment by -0.4 Nm/kg (-0.7 to -0.2; *n* = 38; *k* = 2; I^2 = 38.4%).

1.5.8 Knee sagittal plane joint moment

Pooled results from two studies indicated that treadmill knee sagittal plane joint moment was not significantly different from lab runway knee sagittal plane joint moment (mean difference -0.3 Nm/kg; -1.0 to 0.4; *n* = 38; *k* = 2; I^2 = 92.3%).

1.5.9 Eccentric ankle power

Pooled results from two studies indicated that treadmill eccentric ankle power was not significantly different from lab runway eccentric ankle power (mean difference -2.3 W/kg; -3.3 to 0.8; *n* = 38; *k* = 2; I^2 = 86.4%).

1.5.10 Peak tibial acceleration

Pooled results from two studies indicated that treadmill peak tibial acceleration was not significantly different from track tibial acceleration (mean difference -4.2 g; -14.0 to 5.6; *n* = 24; *k* = 2; I^2 = 92.8%). When one outlier study was removed from this subgroup analysis, the results of one study showed that treadmill peak tibial acceleration was not significantly different from track tibial acceleration (mean difference 0.7 g; -2.5 to 3.9; *n* = 4; *k* = 1). Results from one study showed that treadmill peak tibial acceleration was not significantly different from concrete peak tibial acceleration (mean difference -0.8 g; -3.5 to 1.9; *n* = 5; *k* = 1). Pooled results from two studies indicated that treadmill peak tibial acceleration was not significantly different from lab runway tibial acceleration (mean difference 0.01 g; -0.02 to 0.2; *n* = 27; *k* = 2; I^2 = 0%). Results from one study showed that treadmill peak tibial acceleration was not significantly different from grass peak tibial acceleration (mean difference 0.5 g; -2.7 to 3.6; *n* = 4; *k* = 1). When pooled across all surfaces, treadmill peak tibial acceleration was not significantly different from overground peak tibial acceleration (mean difference -0.8 g; -2.8 to 1.3; *n* = 60; *k* = 7; I^2 = 93.3%). When one outlier was removed from this analysis, treadmill peak tibial acceleration was still not significantly different from overground peak tibial acceleration (mean difference 0.01 g; -0.18 to 0.21; *n* = 40; *k* = 6; I^2 = 0%). Treadmill running speed, motor power and belt length and width were no statistically significant moderators of the mean difference in peak tibial acceleration when the outlier was included (β= -0.68 g; -1.96 to 0.59; β= -0.91 g; -2.36 to 0.54; β= -1.44 g; -6.34 to 3.46; β= -9.20 g; -20.8 to 2.36, respectively) or when it was excluded (β= 0.48 g; -0.23 to 0.32; β= 0.19 g; -0.19 to 0.65; β= 0.56 g; -0.60 to 1.71; β= -0.01 g; -5.79 to 5.77, respectively).
